# Aberrant N-glycolylneuraminic acid in breast MCF-7 cancer cells and cancer stem cells

**DOI:** 10.3389/fmolb.2022.1047672

**Published:** 2022-11-07

**Authors:** Wenqian Yang, Yuan Jiang, Qulian Guo, Zhixin Tian, Zhigang Cheng

**Affiliations:** ^1^ Department of Anesthesiology, Xiangya Hospital, Central South University, Changsha, China; ^2^ School of Chemical Science and Engineering, Tongji University, Shanghai, China

**Keywords:** N-glycolylneuraminic acid, N-glycoproteomics, breast cancer, MCF-7, MCF-7 cancer stem cells

## Abstract

N-Glycolylneuraminic acid (Neu5Gc) is not normally detected in humans because humans lack the hydroxylase enzyme that converts cytidine-5′-monophosphate-N-acetylneuraminic acid (CMP-Neu5Ac) to CMP-Neu5Gc; thus, any Neu5Gc appearing in the human body is aberrant. Neu5Gc has been observed in human cancer cells and tissues. Moreover, antibodies against Neu5Gc have been detected in healthy humans, which are obstacles to clinical xenotransplantation and stem cell therapies. Thus, the study of Neu5Gc in humans has important pathological and clinical relevance. Here, we report the N-glycoproteomics characterization of aberrant Neu5Gc in breast MCF-7 cancer cells and cancer stem cells (CSCs) at the molecular level of intact N-glycopeptides, including comprehensive information (peptide backbones, N-glycosites, N-glycan monosaccharide compositions, and linkage structures) based on a target-decoy theoretical database search strategy and a spectrum-level false discovery rate (FDR) control ≤1%. The existence of Neu5Gc on N-glycan moieties was further confirmed according to its characteristic oxonium fragment ions in the MS/MS spectra of either *m/z* 308.09816 (Neu5Gc) or 290.08759 (Neu5Gc-H_2_O). The results are an important addition to previously reported Neu5Ac data and can be further validated with targeted MS methods such as multiple and parallel reaction monitoring and biochemical methods such as immunoassays. This MS-based N-glycoproteomics method can be extended to the discovery and characterization of putative aberrant Neu5Gc in other biological and clinical systems.

## Graphical Abstract

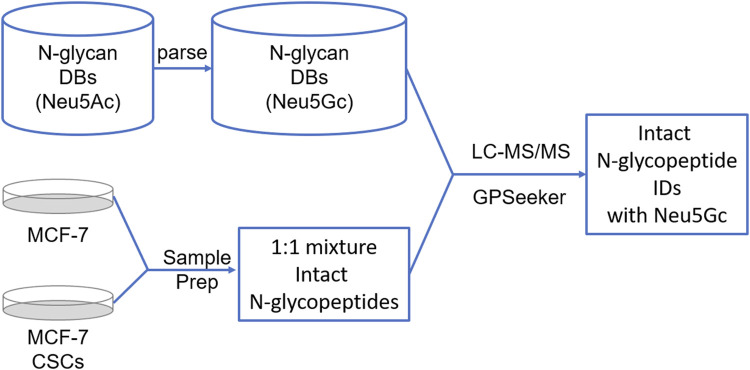

## Introduction

Due to a single-exon deletion mutation in the enzyme cytidine monophosphate-N-acetylneuraminic acid hydroxylase (cmah or CMAH) that encodes the hydroxylase enzyme that converts CMP-Neu5Ac to CMP-Neu5Gc, Neu5Gc (N-glycolylneuraminic acid) is not synthesized in humans. However, food-source Neu5Gc may be metabolically incorporated and eventually present on the surface or secreted as glycans, glycoproteins, and glycolipids in humans; this external Neu5Gc, as a “xeno-autoantigen,” interacts with circulating anti-Neu5Gc “xeno-autoantibodies” and may not only lead to resistance to Neu5Gc-containing biotherapeutics, bio-devices, or xenografts but also cause chronic inflammation (“xenosialitis”) that contributes to malignancies such as cardiovascular diseases, autoimmunity, and cancers. Cmah^−/−^Ldlr^−/−^ mice, immunized with Neu5Gc-bearing antigens producing human-like anti-Neu5Gc antibodies, showed an approximately 2.4-fold increased rate of atherosclerosis on a Neu5Gc-rich high-fat diet (HFD) compared to mice fed Neu5Ac-rich or Sias-free HFD. Cmah^−/−^Ldlr^−/−^ mice are low-density lipoprotein receptor-deficient (Ldlr^−/−^) with human-like cmah deficiency (Kawanishi et al., 2019). The underlying mechanism of these findings was the elevated expression of macrophage cytokines. Increased cancer risk due to the higher consumption of red meat has been consistently reported, as well as aberrant Neu5Gc observed in cancer cells and tissues. Extensive enrichment of Neu5Gc in extracellular sialoglycoproteins of human cancer cells has been detected by fluorometric HPLC (Inoue et al., 2010). In JHOC-5 (clear cell adenocarcinoma of the ovary) human cell lines, Krukenberg tumors metastasized from the stomach to the ovaries, while Neu5Gc levels of 20%, 4%, 3%, and 6% were reported in HeLa (human cervical carcinoma) and PA-1 (ovarian teratocarcinoma of human) cells, respectively. With differential centrifugation of the homogenate of JHOC-5 cells and separation of soluble (cytosol) and insoluble (pellets, P1–P5 five fractions) glycoconjugates in the subcellular compartments, 10–20% intracellular Neu5Gc was also enriched in the P2 (600 g) and P4 (20,000 g) fractions (Inoue et al., 2010). Using an engineered Neu5Gc-specific lectin SubB2M, seral Neu5Gc biomarkers were also detected in both ovarian (Shewell et al., 2018) and breast (Shewell et al., 2022) cancer.

Mass spectrometry-based N-glycoproteomics with isotopic labeling is a state-of-the-art instrumental platform for the comprehensive qualitative and quantitative site- and structure-specific characterization of protein N-glycosylation at the intact N-glycopeptide level, and has also been applied in studying aberrant N-glycosylation in liver cancer (Xiao and Tian, 2019), breast cancer (Wang et al., 2020; Xu et al., 2020; Xue et al., 2020; Yang et al., 2021), pancreatic cancer (Lu et al., 2021), ovarian cancer (Hu et al., 2020; Pan et al., 2020), and pancreatic ductal adenocarcinoma (Cao et al., 2021).

Herein, we report the results of our exploration of N-glycoproteomics for the high-throughput characterization of aberrant Neu5Gc using breast MCF-7 cancer cells and cancer stem cells (CSCs) as the benchmark system. We identified differentially expressed N-glycoproteins in MCF-7 CSCs relative to MCF-7 cells, which we propose to be the characteristics of MCF-7 CSCs. CSCs, as a small population of stem-like cells with the abilities to self-renew, differentiate, and induce tumorigenesis, are responsible for tumor expansion, mutation accumulation, therapeutic resistance, metastasis, and recurrence.

## Experimental

Data on N-acetylneuraminic acid (Neu5Ac) from C18-RPLC-MS/MS raw datasets (including three technical replicates) were obtained from a previous study of differential sialylation in MCF-7 CSCs (relative to MCF-7 cells) (Wang et al., 2019). The MCF-7 cells were cultured in DMEM with 10% fetal bovine serum, 100 U/mL penicillin, and 100 μg/mL streptomycin at 37°C and 5% CO_2_. The MCF-7 CSCs were cultured using a MammoCult™ Human Medium Kit according to the manufacturer’s protocol. CD44 and CD24 were used as cell-surface markers to isolate MCF-7 CSCs by flow cytometry. Higher energy collisional dissociation (HCD) with stepped normalized collisional energies (NCEs) of 20%, 30%, and 40% was applied, in which under the low NCE condition (20%), the N-glycan moiety dissociates while the peptide backbone remains intact (no dissociation); under the high NCE conditions (30% and 40%), the peptide backbone dissociates while the N-glycan moiety is lost except for the N-acetylglucosamine adjacent to the peptide backbone. The datasets were downloaded from the ProteomeXchange Consortium *via* the PRIDE (Perez-Riverol et al., 2019) partner repository (dataset identifier PXD013836).

### Construction of a theoretical N-glycan DB with Neu5Gc

To identify aberrant Neu5Gc in MCF-7 cancer cells and CSCs, a theoretical N-glycan database containing 37,416 entries was first built from the previous human N-glycan DB with only Neu5Ac and 75,888 entries. Equal probability was given to Neu5Ac and Neu5Gc because they are not distinguished by their transferases (Supplementary Figure S1). Among these, 26,000 entries contained only Neu5Gc, while 11,416 entries had both Neu5Gc and Neu5Ac. Evaluation of the distribution of the number of Neu5Gc per entry showed that 31,240, 5,688, 468, and 20 entries contained one, two, three, and four Neu5Gc molecules, respectively. Complex and hybrid N-glycans accounted for 97.9% and 2.1% of the entries, while di-, tri-, and tetra-sialylation accounted for 3.3%, 36.2%, 3.4%, and 60.6% of the entries, respectively.

### DB search for intact N-glycopeptides with Neu5Gc in both MCF-7 cancer cells and CSCs

Target and decoy theoretical customized human intact N-glycopeptides databases were first created using the entire human protein database (20,376 entries downloaded from UniProt at www.uniprot.org with the criteria of “homo sapiens” and “Reviewed”) and the aforementioned theoretical N-glycan DB-containing Neu5Gc. Each dataset was then searched against the two databases independently. The search parameters for both the precursor and fragment ions were isotopic abundance cutoff (IPACO), 40%; isotopic peak *m/z* deviation (IPMD), 20 ppm; and isotopic abundance deviation (IPAD), 50%. Initial intact N-glycopeptide spectrum matches (GPSMs) were obtained using the following refinement criteria: Y1 ions, Top 10; minimal percentage of matched fragment ions of N-glycosite-containing peptides, ≥10%; minimal matched product ions of N-glycan, no less than one; and TopN hits (Top 1 hits have the lowest P score), two. The characteristic oxonium ions of Neu5Ac, Neu5Ac-H_2_O, Neu5Gc, and Neu5Gc-H_2_O with exact theoretical *m/z* values of 292.10324, 274.09268, 308.09816, and 290.08759, respectively, were also simultaneously searched, with a mass tolerance of 20 ppm.

For each dataset, the target and decoy GPSMs from each technical RPLC-MS/MS dataset were filtered with the observation of at least one oxonium ion for both Neu5Ac and Neu5Gc, combined and ranked with increasing P score, and grouped with the criteria of “peptide sequence, N-glycosite, and N-glycan linkage” to remove duplicates and generate the final list of intact N-glycopeptide IDs.

### Annotation analysis

Annotation analyses of the N-glycoproteins corresponding to the intact N-glycopeptides with Neu5Gc identified from MCF-7 cancer cells and CSCs were performed independently. Gene ontology analyses of molecular functions and biological processes were carried out using the PANTHER Classification System (pantherdb.org). Subcellular localization was obtained from CELLO (http://cello.life.nctu.edu.tw/). KEGG pathway and domain annotations were performed using the DAVID functional annotation tool (http://david.ncifcrf.gov). The protein–protein interaction (PPI) information was retrieved using STRING software (http://string-db.org/).

## Results and discussion

RPLC-MS/MS (HCD with stepped normalized collision energies) analysis of 1:1 (w/w) intact N-glycopeptide mixtures of MCF-7 cancer cells and CSCs was previously carried out with three technical replicates, which revealed intact N-glycopeptide IDs with Neu5Ac. In the present study, we report our re-search of the three datasets for possible aberrant Neu5Gc using a customized theoretical N-glycan database containing 37,416 monosaccharide sequence entries, in which each sequence had at least one Neu5Gc with or without additional co-occurring Neu5Ac. Based on the criteria of observation of at least one characteristic oxonium ion and spectrum-level FDR control of ≤1%, 143 and 221 intact N-glycopeptides with Neu5Gc were identified from MCF-7 cancer cells and CSCs, respectively.

**FIGURE 1:**
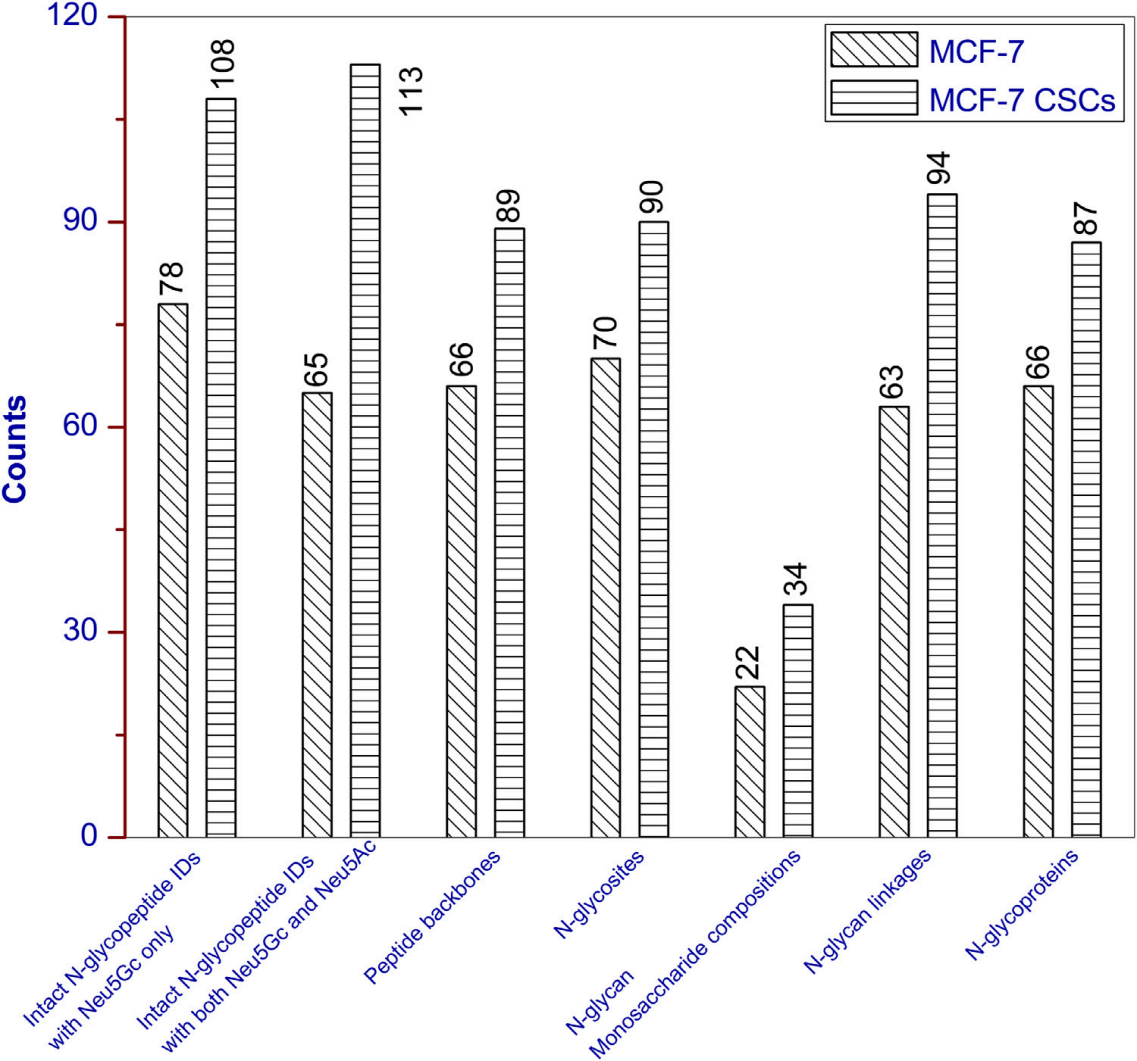
Unique intact N-glycopeptides with Neu5Gc and corresponding unique peptides, N-glycosites, N-glycan monosaccharide compositions, N-glycan linkages, and N-glycoproteins identified from MCF-7 and MCF-7 CSCs.

The detailed information for each intact N-glycopeptide ID (including accession number, N-glycosite, peptide backbone, monosaccharide composition, and linkages) are provided in Supplementary Tables S1, S2. The extracted ion chromatograms (EICs) for the characteristic oxonium ions of Neu5Ac-H_2_O, Neu5Ac, NeuGAc-H_2_O, and Neu5Gc were queries with mass ranges of 274.09–274.10, 292.10–292.11, 290.08–290.09, and 308.09–308.10, respectively, and the resulting EICs from the three RPLC-MS/MS technical replicates are presented in Supplementary Figures S2–S4. For MCF-7 cancer cells, the 143 intact N-glycopeptide IDs resulted from the combination of 66 unique peptide backbones and 63 monosaccharide linkages (22 monosaccharide compositions). N-Glycosylation occurred on 70 N-glycosites of 66 N-glycoproteins (Figure 1). More than half of the IDs (78) contained Neu5Gc only; the remaining 65 IDs contained both Neu5Gc and Neu5Ac. For MCF-7 CSCs, the 221 intact N-glycopeptide IDs resulted from the combination of 89 unique peptide backbones and 94 monosaccharide linkages (34 monosaccharide compositions). N-glycosylation occurred on 90 N-glycosites of 87 N-glycoproteins (Figure 1). About half of the IDs (108) contained Neu5Gc only; the remaining 113 IDs contained both Neu5Gc and Neu5Ac.

**FIGURE 2:**
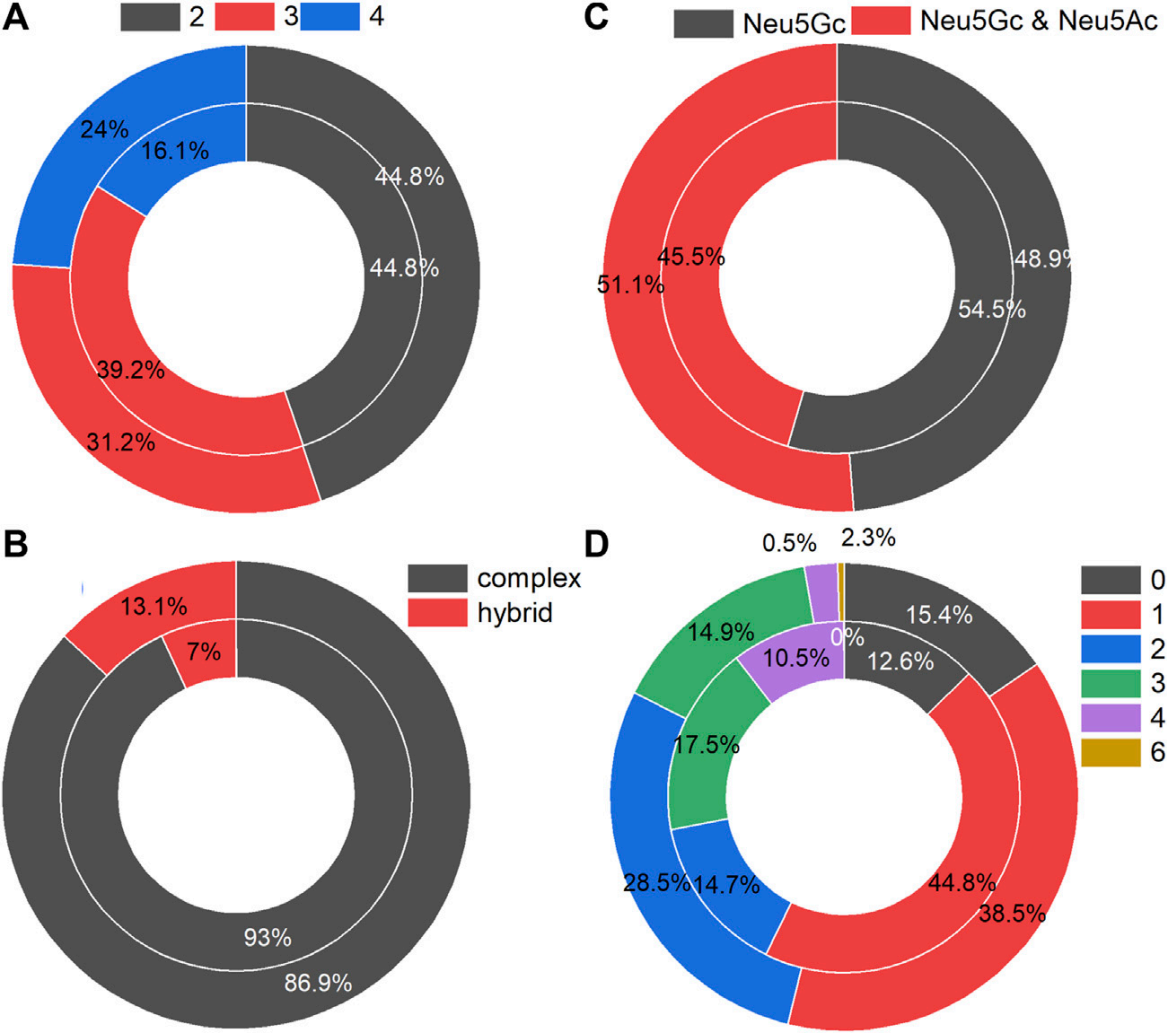
Statistics of N-glycans in intact N-glycopeptides with Neu5Gc identified from MCF-7 (inner circle) and MCF-7 CSCs (outer circle): **(A)** number of branches per N-glycan, **(B)** with Neu5Gc only or additional Neu5Ac, **(C)** N-glycan-type, **(D)** number of fucoses per N-glycan.

The N-glycans on the identified intact N-glycopeptides from MCF-7 and MCF-CSCs showed similar distribution patterns in terms of branches (Figure 2A), type (Figure 2B), with or without Neu5Ac (Figure 2C), and number of fucoses (Figure 2D). Approximately 45% of N-glycans had two branches, the N-glycans were predominantly the complex type, approximately half of the N-glycans contained only Neu5Gc, and most of the N-glycans contained one fucose.

**FIGURE 3:**
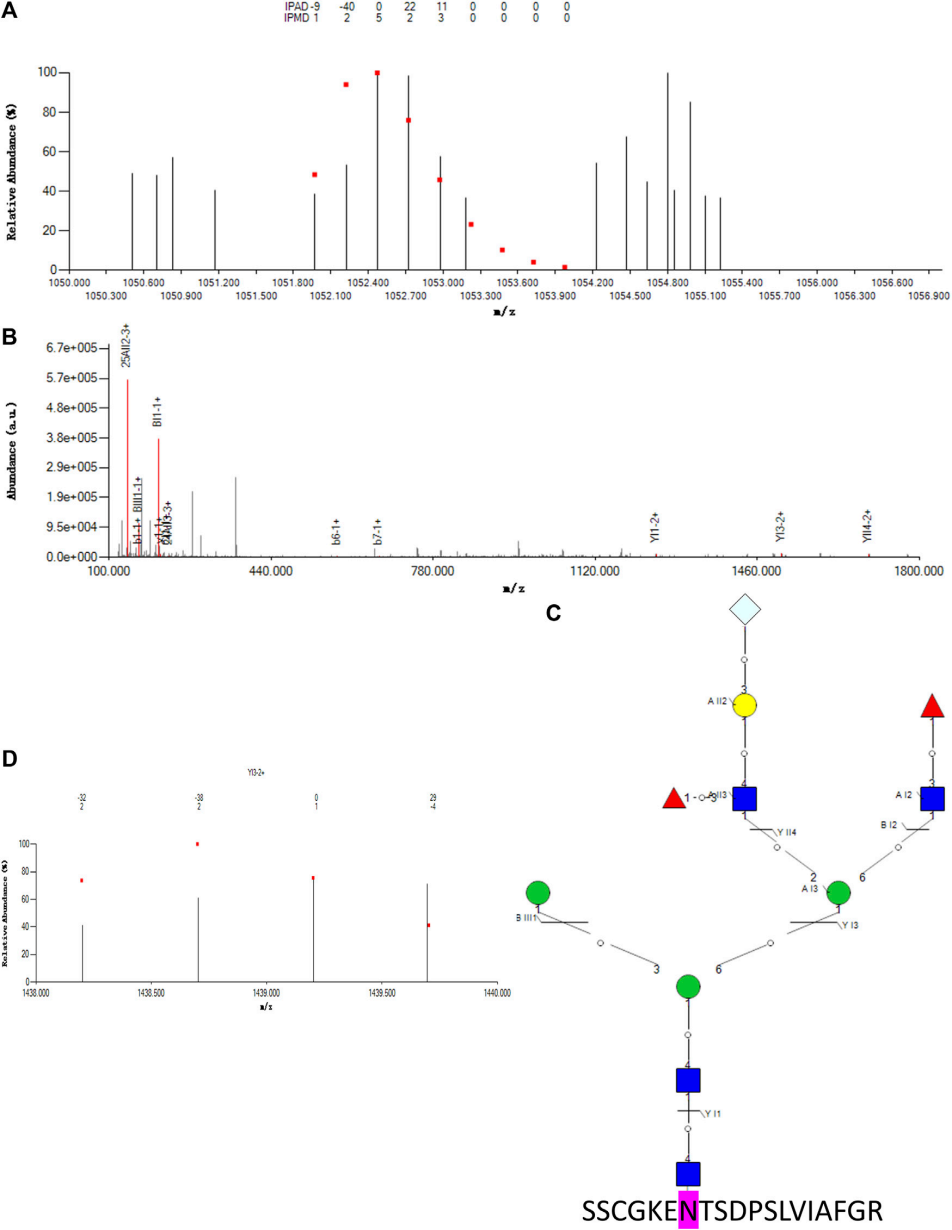
Example intact N-glycopeptide SSCGKENTSDPSLVIAFGR with a complex N-glycan 01Y41Y41M(31M)61M(21Y(31F)41L32T)61Y31F containing Neu5Gc identified from N-glycosite N84 of lysosome-associated membrane glycoprotein 1 (LAMP1_HUMAN, P11279). **(A)** Theoretical (dots) and experimental isotopic envelope fingerprinting map of the precursor ion. **(B)** Annotated MS/MS spectrum with the matched fragment ions from both the peptide backbone and N-glycan moiety. **(C)** Graphical fragmentation map of the N-glycan moiety. **(D)** Theoretical (dots) and experimental isotopic envelope fingerprinting map of the structure-diagnostic fragment ion YI3. 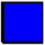
= N-acetylglucosamine (Y), 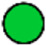
= mannose (M), 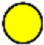
= galactose (L), 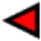
= fucose (F), 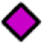
= N-acetylneuraminic acid (S), 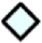
= N-glycolylneuraminic acid (T); IPAD = isotopic peak abundance deviation, IPMD = isotopic peak M/z deviation; a.u. = arbitrary unit.

As an example of an intact N-glycopeptide ID with a complex N-glycan containing Neu5Gc, SSCGKENTSDPSLVIAFGR was identified (01Y41Y41M(31M)61M(21Y(31F)41L32T)61Y-31F) from MCF-7 CSCs (Figure 3) and showed a good match between the experimental and theoretical isotopic envelopes of the precursor ion (Figure 3A). The matched fragment ions from both the peptide backbone and the N-glycan moiety were annotated in the MS/MS spectrum (Figure 3B). Nine matched fragment ions were observed for the N-glycan moiety (Figure 3C), while one structure-diagnostic fragment ion (YI3) was observed for the N-glycan sequence structure (Figure 3D). The monosaccharide composition was N4H4F2S0T1. The six additional six sequence structures in the aforementioned theoretical N-glycan DB included (01Y(61F)41Y41M(31M41Y31F)61M61Y41L32S,01Y(61F)- 41Y41M(31M41Y)61M21Y(31F)41L32S, 01Y(61F)41Y41M- (31M)61M(21Y41L32S)61Y31F, 01Y(61F)41Y41M(31M)- (41Y)61M21Y(31F)41L32S, 01Y41Y41M(31M41Y31F)61M- 21Y(31F)41L32S, and 01Y(61F)41Y41M(31M)61M(21Y- (31F)41L32S)61Y). The N-glycosite was N84 of lysosome-associated membrane glycoprotein 1 (LAMP1_HUMAN, P11279).

**FIGURE 4:**
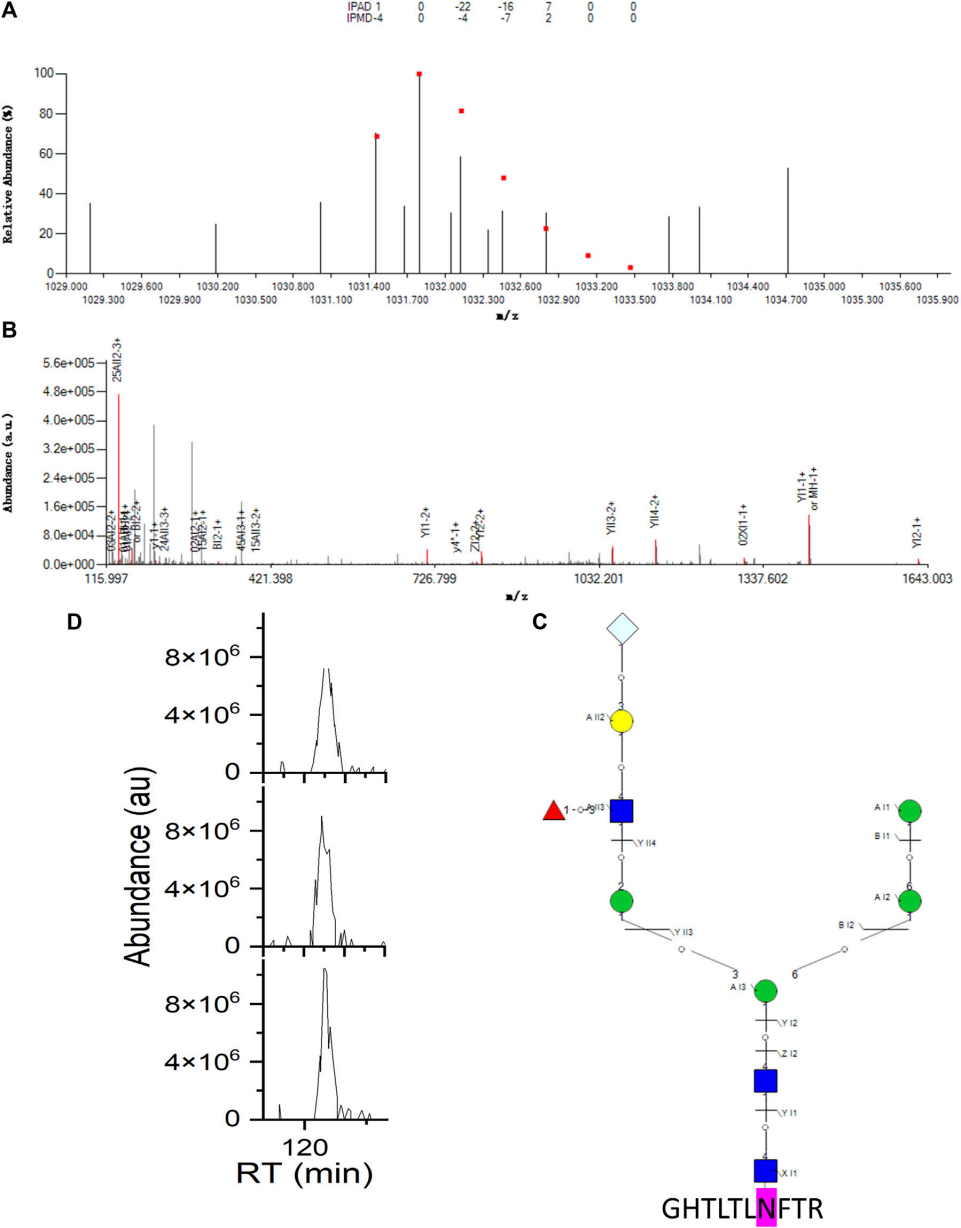
Example intact N-glycopeptide GHTLTLNFTR with a hybrid N-glycan 01Y41Y41M(31M21Y(31F)41L32T)61M-61M containing Neu5Gc identified from N-glycosite N103 of lysosome-associated membrane glycoprotein 1 (LAMP1_HUMAN, P11279). **(A)** Theoretical (dots) and experimental isotopic envelope fingerprinting map of the precursor ion. **(B)** Annotated MS/MS spectrum with the matched fragment ions from both the peptide backbone and N-glycan moiety. **(C)** Graphical fragmentation map of the N-glycan moiety. **(D)** Extracted ion chromatograms (EICs) of the precursor ion among three technical replicates of the RPLC-MS/MS analysis. 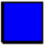
= N-acetylglucosamine (Y), 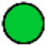
= mannose (M), 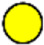
= galactose (L), 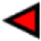
= fucose (F), 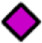
= N-acetylneuraminic acid (S), 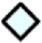
= N-glycolylneuraminic acid (T); IPAD = isotopic peak abundance deviation, IPMD = isotopic peak M/z deviation, a.u. = arbitrary unit, RT = retention time.

As an example of an intact N-glycopeptide ID with hybrid N-glycan-containing Neu5Gc, on another N-glycosite N103 of LAMP1, hybrid N-glycan 01Y41Y41M(31M21Y(31F)41L32T) -61M61M containing Neu5Gc was identified with the peptide backbone GHTLTLNFTR (Figure 4). The fucose was on the branch, and the corresponding core isomer 01Y(61F)41Y41M(31M41Y41L32T)61M61M was co-identified (Supplementary Figure S5). These two fucose position isomers were distinguished by 11 (^2,4^AII3, ^1,5^AII3, ZI1, YI1, ZI2, YI2, YII3, YII4, YI1, YI2, YII4) and one (^0,1^AII4) structure-diagnostic fragment ion, respectively. The two sequence structures were the only two sequence structures in the aforementioned theoretical N-glycan DB sharing the same monosaccharide composition of N3H5F1S0T1. Regarding the nomenclature of the N-glycan fragment ions, the two numbers on the upper left corners represent the bond cleavage position of the intra-ring fragmentation; the capital English letters represent the types of fragment ions; the middle Roman numbers represent the branch positions; and the Arabic numbers represent how many monosaccharides are contained in each fragment ion.

LAMP1 has 18 putative N-glycosites with the N-X-S/T/C (X≠P) motif, all of which have been annotated in UniProt. Heavy glycosylation protects against intracellular proteolysis. In addition to the aforementioned monosaccharide compositions of N4H4F2S0T1 on N84 and N3H5F1S0T1 on N103, N5H5F2S1T1, N4H4F2S0T1, and N6H6F2S1T1 were also identified on N84 in MCF-7 CSCs (Supplementary Table S2). N-glycosylation with Neu5Gc was not observed in MCF-7 in this study. In the previous search of theoretical N-glycan DB with Neu5Ac only (Wang et al., 2019), five compositions (N2H3F1S0, N5H6F3S1, N4H5F1S2, N5H6F1S2, and N5H6F1S3) were observed for N84; seven compositions (N2H6F0S0, N2H8F0S0, N3H6F0S0, N2H7F0S0, N3H6F0S1, N3H5F2S0, and N3H5F0S1) were observed for N103; and three compositions (N2H7F0S0, N2H8F0S0, and N2H9F0S0) were observed for N322. Previous reverse transcription-quantitative polymerase chain reaction (RT-qPCR) characterization of 20 pairs of fresh-frozen breast cancer and corresponding non-cancerous tissues together with tissue microarray immunohistochemistry (TMA-IHC) of 143 paired tissues showed the significant upregulation of LAMP1 in cancer tissue (Wang et al., 2017). LAMP1 expression, molecular classification, and TNM stage were independent prognostic factors for overall survival; thus, LAMP1 was proposed as a novel prognostic factor in patients with breast cancer.

**FIGURE 5:**
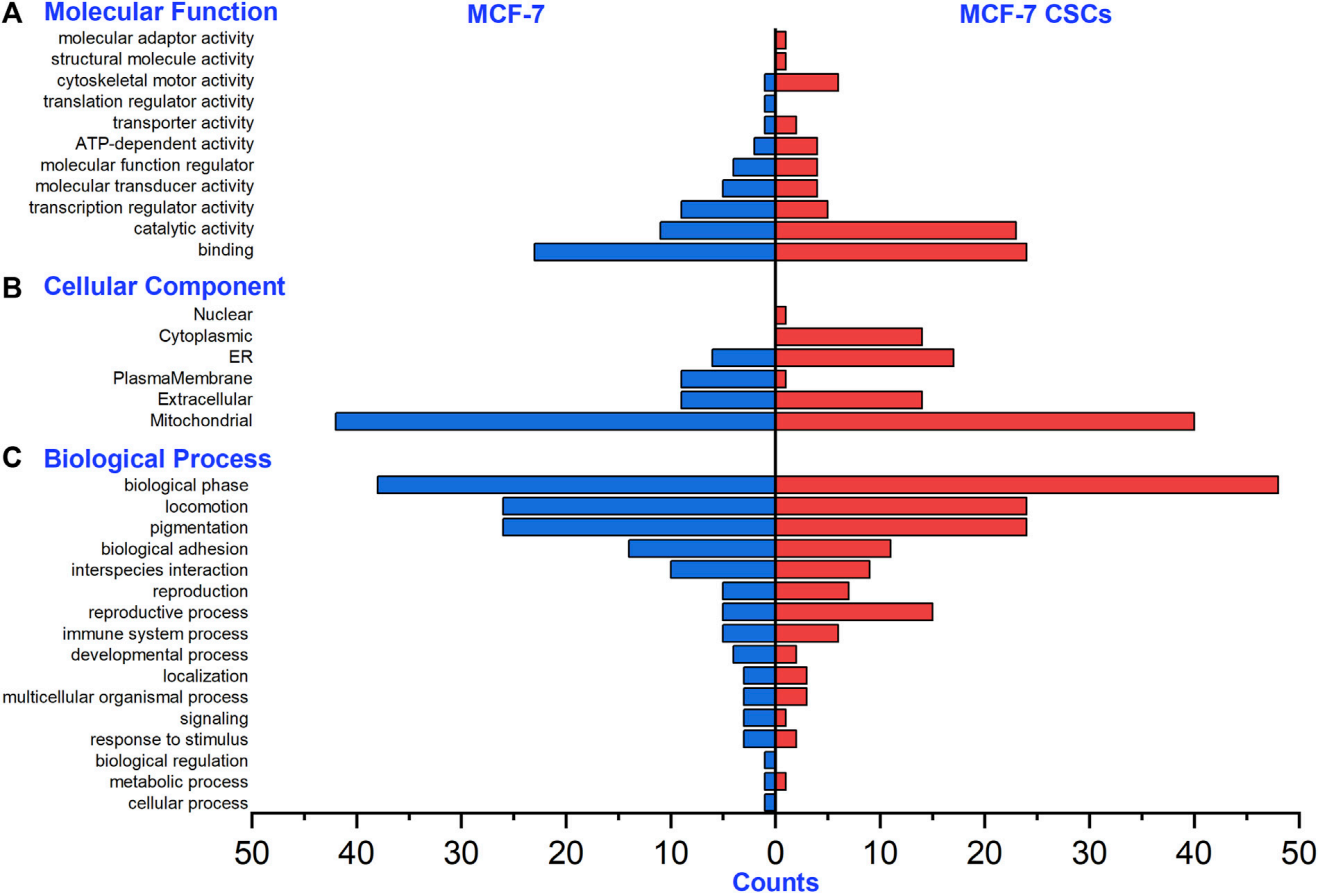
Gene ontology analysis of N-glycoproteins with Neu5Gc identified from MCF-7 and MCF-7 CSCs. **(A)** Molecular functions, **(B)** cellular components, **(C)** biological processes.

Regarding the N-glycoproteins corresponding to the intact N-glycopeptides with Neu5Gc identified from MCF-7 and MCF-7 CSCs, the molecular functions showed enrichment mainly in binding and catalytic activity (Figure 5A); subcellular localization mainly showed mitochondrial enrichment (Figure 5B); and the biological processes mainly showed enrichment of the biological phase, locomotion, and pigmentation (Figure 5C). For example, LAMP1 is a lysosome-specific integral membrane protein and belongs to the protein class of membrane-trafficking regulatory proteins, which regulate secretory vesicle release.

**FIGURE 6:**
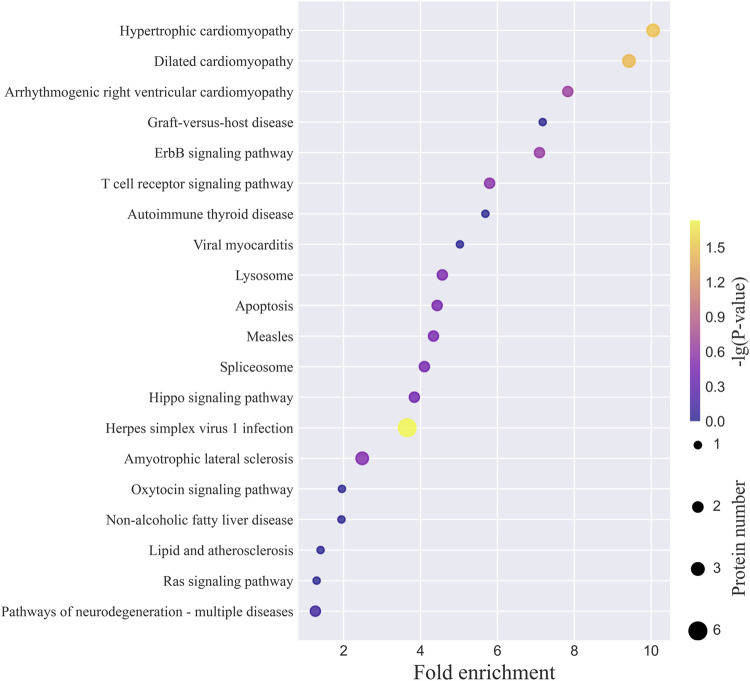
Top 20 KEGG pathways of N-glycoproteins corresponding to intact N-glycopeptides with Neu5Gc identified from MCF-7 cancer cells.

The top 20 KEGG pathways for the N-glycoproteins corresponding to the intact N-glycopeptides with Neu5Gc identified from MCF-7 and MCF-7 CSCs are presented in Figure 6 and Supplementary Figure S6. The complete lists are provided in Supplementary Tables S3, S4. Herpes simplex virus 1 infection, hypertrophic cardiomyopathy, dilated cardiomyopathy, metabolic pathways, amyotrophic lateral sclerosis, and thyroid hormone synthesis were among the top 3 pathways. LAMP1 and CD14, both identified in this study, are involved in the phagosome pathway (Supplementary Figure S7). One of the major functions of monocyte differentiation antigen CD14 is to act *via* MyD88, TIRAP, and TRAF6, leading to NF-kappa-B activation, cytokine secretion, and the inflammatory response (Haziot et al., 1996). In this study of MCF-7 CSCs, we observed hybrid N-glycan 01Y41Y41M(31M21Y(31F)41L32T)61M61M with one Neu5Gc residue on N-glycosite N151. In addition to CD14, five other cytokine-related N-glycoproteins (CD55, CD44, CSET, domain-containing protein 1B, and histone acetyltransferase KAT2A) showed aberrant Neu5Gc in MCF-7 CSCs (Supplementary Table S2). Through its ectodomain, CD44 engages extracellular matrix components such as hyaluronan/HA, collagen, growth factors, cytokines, or proteases and serves as a platform for signal transduction by assembling protein complexes containing receptor kinases and membrane proteases *via* its cytoplasmic domain (Casalino-Matsuda et al., 2009). CD55 (also known as complement decay-accelerating factor) is involved in the positive regulation of T-cell cytokine production. In MCF-7 cells, two cytokine-related N-glycoproteins (CD80 and interleukin-16) showed aberrant Neu5Gc (Supplementary Table S1). CD80 is involved in the co-stimulatory signal essential for T-lymphocyte activation as well as T-cell proliferation; moreover, cytokine production is induced by the binding of CD28 (Vandenborre et al., 1999). In this study of MCF-7 cells, complex N-glycan with monosaccharide composition N6H6F0S1T1 and one Neu5Gc residue was observed. With Neu5Gc-containing N-glycan N5H5F2S1T1 identified on N-glycosite N131 in MCF-7 cells in this study, leukocyte C-terminal Src kinase (LCK_HUMAN, P06239) was enriched in the PD-L1 expression and PD-1 checkpoint pathway in cancer (Supplementary Figure S8). Constitutively associated with the cytoplasmic portions of the CD4 and CD8 surface receptors, CLK plays a key role in T-cell antigen receptor (TCR)-linked signal transduction pathways. With Neu5Gc-containing N-glycan N6H5F3S1T1 identified on N-glycosite N4783 in MCF-7 CSCs in this study, homeobox protein Hox-B1 (HXB1_HUMAN, P14653) was enriched in the signaling pathways regulating stem cell pluripotency (Supplementary Figure S9). As a sequence-specific transcription factor, HXB1 is involved in the developmental regulatory system that provides cells with specific positional identities on the anterior–posterior axis.

**FIGURE 7:**
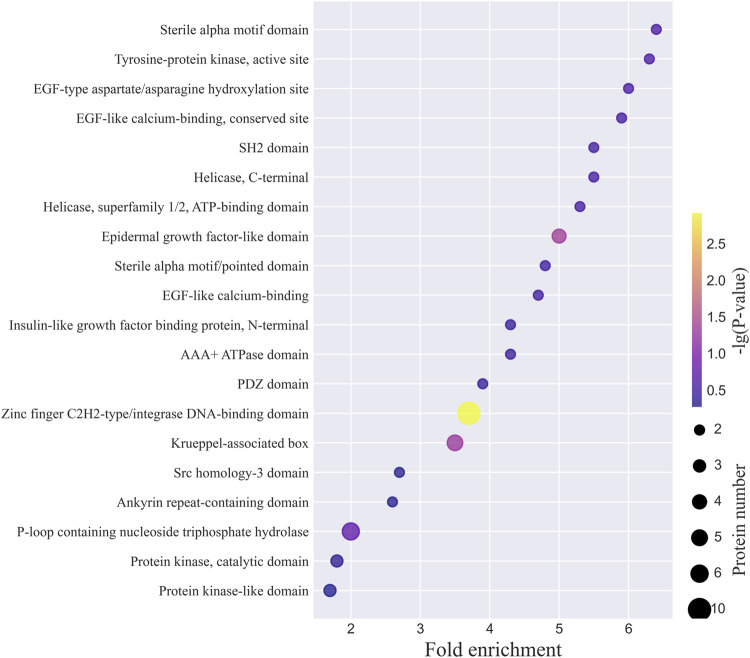
Top 20 INTERPRO domains of N-glycoproteins corresponding to intact N-glycopeptides with Neu5Gc identified from MCF-7 cancer cells.

The top 20 INTERPRO domains for the N-glycoproteins corresponding to the intact N-glycopeptides with Neu5Gc identified from MCF-7 and MCF-7 CSCs are shown in Figure 7 and Supplementary Figure S10. The complete lists are provided in Supplementary Tables S5, S6. Zinc finger C2H2-type/integrase DNA-binding domain, P-loop containing nucleoside triphosphate hydrolase, and Krueppel-associated box were among the top 3 domains in both MCF-7 cancer cells and MCF-7 CSCs. For example, LAMP1 was classified as the lysosome-associated membrane glycoprotein, conserved site (LAMP_CS) entry, where one signature is centered on the first conserved cysteine of the duplicated domains and the other is a complex region including the extremity of the second domain, the totality of the transmembrane region, and the cytoplasmic tail. Lamp proteins have two internally homologous lysosome-luminal domains separated by a proline-rich hinge region and one C-terminal transmembrane region followed by a very short cytoplasmic tail. In mammals, LAMP1 and LAMP2 are the major components of the lysosome membrane.

The PPI networks of the N-glycoproteins corresponding to the intact N-glycopeptides with Neu5Gc identified from MCF-7 cancer cells and CSCs are shown in Supplementary Figures S11, S12. K-means clustering with three clusters and medium confidence was performed, and only connected nodes were kept and shown. LAMP1 showed direct interactions with discoidin domain receptor tyrosine kinase 1 (DDR1, Q13637) and CD44 (P16070). DDR1 plays a role in the maturation of phagosomes that engulf pathogens. CD44, with an affinity for hyaluronic acid (HA), mediates cell–cell and cell–matrix interactions and plays important roles in cell migration and tumor growth and progression.

With the characteristics of low stoichiometry, micro- and macro-heterogeneity of N-glycosites, as well as complex N-glycan sequence structures, N-glycosylation in complex N-glycoproteome systems has mainly been qualitatively and quantitatively characterized by MS-based N-glycoproteomics pipelines at the molecular level of intact N-glycopeptides. Using this method, lowly abundant intact N-glycopeptides can be efficiently enriched by hydrophilic materials, N-glycosites can be efficiently characterized with site-determining fragment ions in the MS/MS spectra from selective fragmentation of the peptide backbones, N-glycan sequence structures can be efficiently characterized with structure-diagnostic fragment ions in the MS/MS spectra from selective fragmentation of the N-glycan moieties, and overall identification confidence is achieved with common target-decoy database search strategy and spectrum-level false discovery rate control. The limitation of this MS-based N-glycoproteomics discovery method is that only partial N-glycosites and N-glycan sequence structures are confirmed due to their limited abundance, fragmentation efficiency, or isomeric structure differences; in addition, although the most comprehensive N-glycosylation site and structure information is achieved at the molecular level of intact N-glycopeptides, information on cross-talk between N-glycosylation of different N-glycosites as well as other post-translational modifications (PTMs, such as common methylation, acetylation, and phosphorylation) across the entire intact protein amino acid sequence is missing. Nevertheless, MS-based N-glycoproteomics is currently the state-of-the-art instrumental analytical pipeline for the comprehensive characterization of N-glycosylation of complex N-glycoproteome systems including those with aberrant Neu5Gc from metabolic intake or pathological origin, especially in this so-called discovery phase. Downstream verification and validation of individual interesting proteins such as disease biomarkers using either targeted MS methods (such as multiple reaction monitoring, MRM) or biochemical methods (such as immunoassays) are currently mature and widely available.

## Conclusion

Aberrant N-glycolylneuraminic acid (Neu5Gc) in breast MCF-7 cancer cells and cancer stem cells (CSCs) was efficiently characterized at the proteome scale by MS-based N-glycoproteomics, in which intact N-glycopeptides were sensitively enriched with a hydrophilic material, separated by high-resolution RPLC, detected by highly efficient tandem MS with selective fragmentation of the peptide backbone and the N-glycan moiety, and analyzed using a site- and structure-specific database search engine. The confidence of this discovery study includes a spectrum-level false discovery rate control using the common target-decoy theoretical database search strategy widely adopted in the proteomics field as well as further confirmation using characteristic oxonium ions of Neu5Gc. Downstream validation can be carried out using targeted MS methods such as multiple and parallel reaction monitoring and biochemical methods such as immunoassays. The results are an important addition to previously reported Neu5Ac data, and this method can be extended to the characterization of other biological and clinical systems with Neu5Gc.

## Data Availability

The original contributions presented in the study are included in the article/Supplementary Material. Further inquiries can be directed to the corresponding author.
