# Supplementary material for: Aberrant N-glycolylneuraminic acid in breast MCF-7 cancer cells and cancer stem cells
Source: Front Mol Biosci. 2022 Nov 7;9:1047672. doi: 10.3389/fmolb.2022.1047672 (PMC9676485; doi:10.3389/fmolb.2022.1047672)
Supplement: Supplementary file 1 [file DataSheet1.docx]

**Supporting Information**

**Aberrant N-glycolylneuraminic acid in breast MCF-7 cancer cells and cancer stem cells**

Wenqian Yang^1^, Yuan Jiang^1^, Qulian Guo^1^, Zhixin Tian^2^, Zhigang Cheng^1^*

^1^ Department of Anesthesiology, Xiangya Hospital, Central South University, Changsha 410008, China

^2^School of Chemical Science and Engineering, Tongji University, Shanghai 200092, China

*Corresponding author, [chengzg2004@hotmail.com](mailto:chengzg2004@hotmail.com).

**Contents**

**Supplemental Figure 1.** Statistics of the theoretical N-glycan database with Neu5Gc. (A) Distribution of N-glycans with Neu5Gc only and N-glycans with both sialic acids of Neu5Gc and Neu5Ac; (B) distribution of number of Neu5Gcs per N-glycan; (C) distribution of N-glycan types of complex and hybrid; (D) distribution of antennas per N-glycan.

**Supplemental Figure 2.** Extract ion chromatograms of the characteristic oxonium ions of Neu5Ac, Neu5Gc-H2O, Neu5Ac, Neu5Gc-H2O with a mass tolerance of 20 ppm in the first technical replicate RPLC-MS/MS analysis of the intact N-glycopeptide mixture of MCF-7 and MCF-7 CSCs.

**Supplemental Figure 3.** Extract ion chromatograms of the characteristic oxonium ions of Neu5Ac, Neu5Gc-H2O, Neu5Ac, Neu5Gc-H2O with a mass tolerance of 20 ppm in the second technical replicate RPLC-MS/MS analysis of the intact N-glycopeptide mixture of MCF-7 and MCF-7 CSCs.

**Supplemental Figure 4.** Extract ion chromatograms of the characteristic oxonium ions of Neu5Ac, Neu5Gc-H2O, Neu5Ac, Neu5Gc-H2O with a mass tolerance of 20 ppm in the third technical replicate RPLC-MS/MS analysis of the intact N-glycopeptide mixture of MCF-7 and MCF-7 CSCs.

**Supplemental Figure 5.** Example intact N-glycopeptide GHTLTLNFTR with a hybrid N-glycan 01Y(61F)41Y41M(31M41Y41L32T)61M61M containing Neu5Gc identified from N-glycosite N103 of Lysosome-associated membrane glycoprotein 1 (LAMP1_HUMAN, P11279);
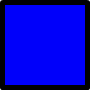
=N-acetylglucosamine (Y),
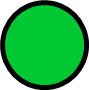
=mannose (M),
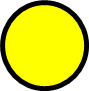
 =galactose (L),
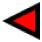
=fucose (F),
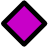
=N-acetylneuraminic acid(S),
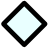
=N-glycolylneuraminic acid (T).

**Supplemental Figure 6.** Top20 KEGG pathways of N-glycoproteins corresponding to intact N-glycopeptides with Neu5Gc identified from MCF-7 CSCs.

**Supplemental Figure 7.** N-glycoproteins LAMP1 and CD14, identified with Neu5Gc in this study, are involved in the phagosome pathway.

**Supplemental Figure 8.** INTERPRO domain of N-glycoproteins corresponding to intact N-glycopeptides with Neu5Gc identified from MCF-7 CSCs.

**Supplemental Figure 9.** PPI networks of the N-glycoproteins corresponding to the intact N-glycopeptides with Neu5Gc identified from MCF-7 cancer cells.

**Supplemental Figure 10.** PPI networks of the N-glycoproteins corresponding to the intact N-glycopeptides with Neu5Gc identified from MCF-7 CSCs.

**Supplemental Figure 11.** PPI networks of the N-glycoproteins corresponding to the intact N-glycopeptides with Neu5Gc identified from MCF-7 cancer cells.

**Supplemental Figure 12.** PPI networks of the N-glycoproteins corresponding to the intact N-glycopeptides with Neu5Gc identified from MCF-7 CSCs.

**Supplemental Table 1.** The detailed information for the intact N-glycopeptides with Neu5Gc identified from breast MCF-7 cancer stem cells; AC=accession number; in N-glycan composition, N=N-acetylglucosamine, H=Hexose, F=fucose, S= N-acetylneuraminic acid, T=N-glycolylneuraminic acid; in N-glycan linkage, Y=N-acetylglucosamine, M=mannose, F=fucose, L=galactose, S= N-acetylneuraminic acid, T=N-glycolylneuraminic acid.

**Supplemental Table 2.** The detailed information for the intact N-glycopeptides with Neu5Gc identified from breast MCF-7 cancer stem cells; AC=accession number; in N-glycan composition, N=N-acetylglucosamine, H=Hexose, F=fucose, S= N-acetylneuraminic acid, T=N-glycolylneuraminic acid; in N-glycan linkage, Y=N-acetylglucosamine, M=mannose, F=fucose, L=galactose, S= N-acetylneuraminic acid, T=N-glycolylneuraminic acid

**Supplemental Table 3.** The complete KEGG pathways of N-glycoproteins corresponding to intact N-glycopeptides with Neu5Gc identified form MCF-7 cancer cells.

**Supplemental Table 4.** The complete KEGG pathways of N-glycoproteins corresponding to intact N-glycopeptides with Neu5Gc identified form MCF-7 cancer stem cells.

**Supplemental Table 5.** The complete INTERPRO domains of N-glycoproteins corresponding to intact N-glycopeptides with Neu5Gc identified form MCF-7 cancer cells.

**Supplemental Table 6.** The complete INTERPRO domains of N-glycoproteins corresponding to intact N-glycopeptides with Neu5Gc identified form MCF-7 cancer stem cells.


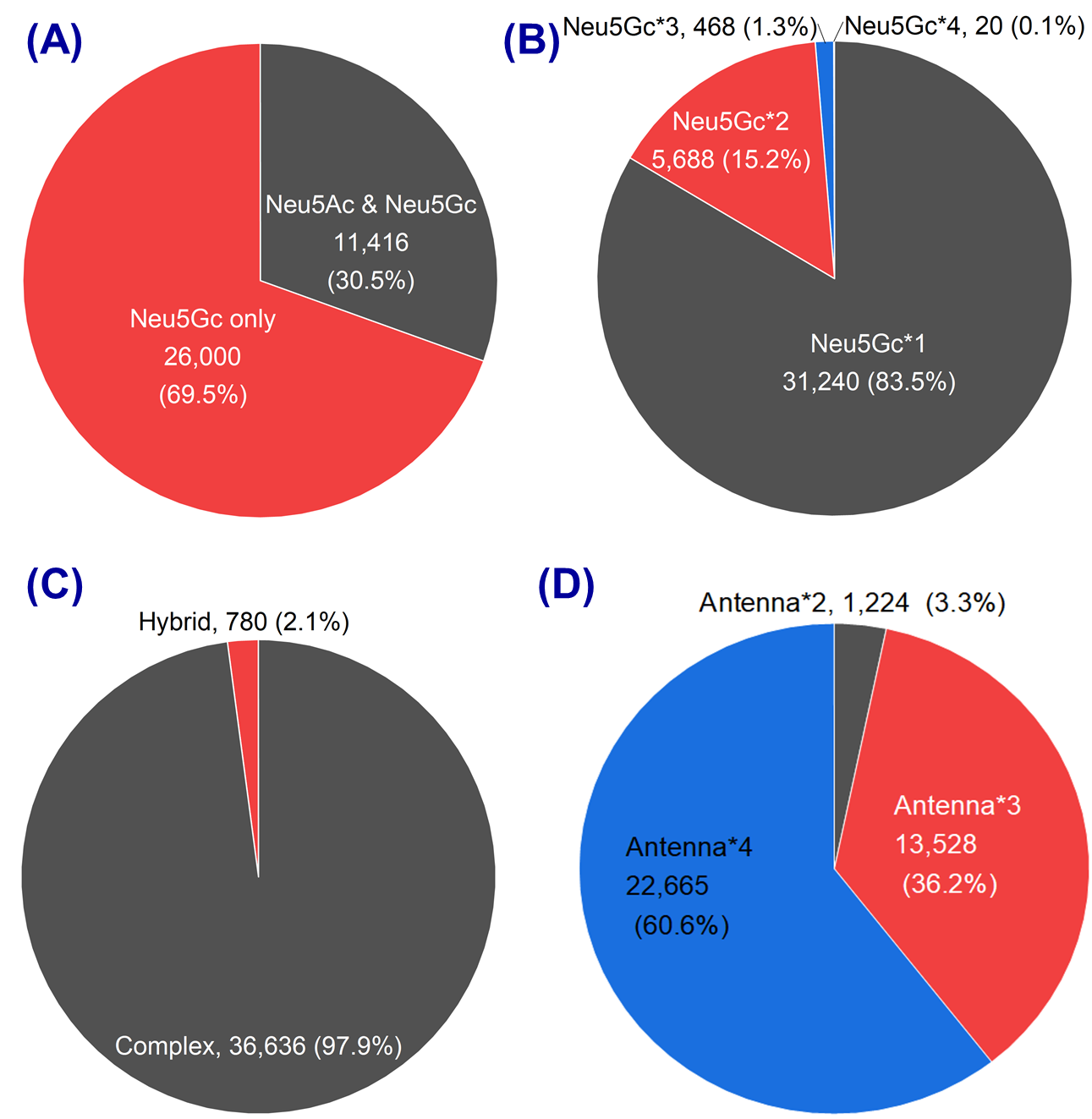


**Supplemental Figure 1.** Statistics of the theoretical N-glycan database with Neu5Gc. (A) Distribution of N-glycans with Neu5Gc only and N-glycans with both sialic acids of Neu5Gc and Neu5Ac; (B) distribution of number of Neu5Gcs per N-glycan; (C) distribution of N-glycan types of complex and hybrid; (D) distribution of antennas per N-glycan.


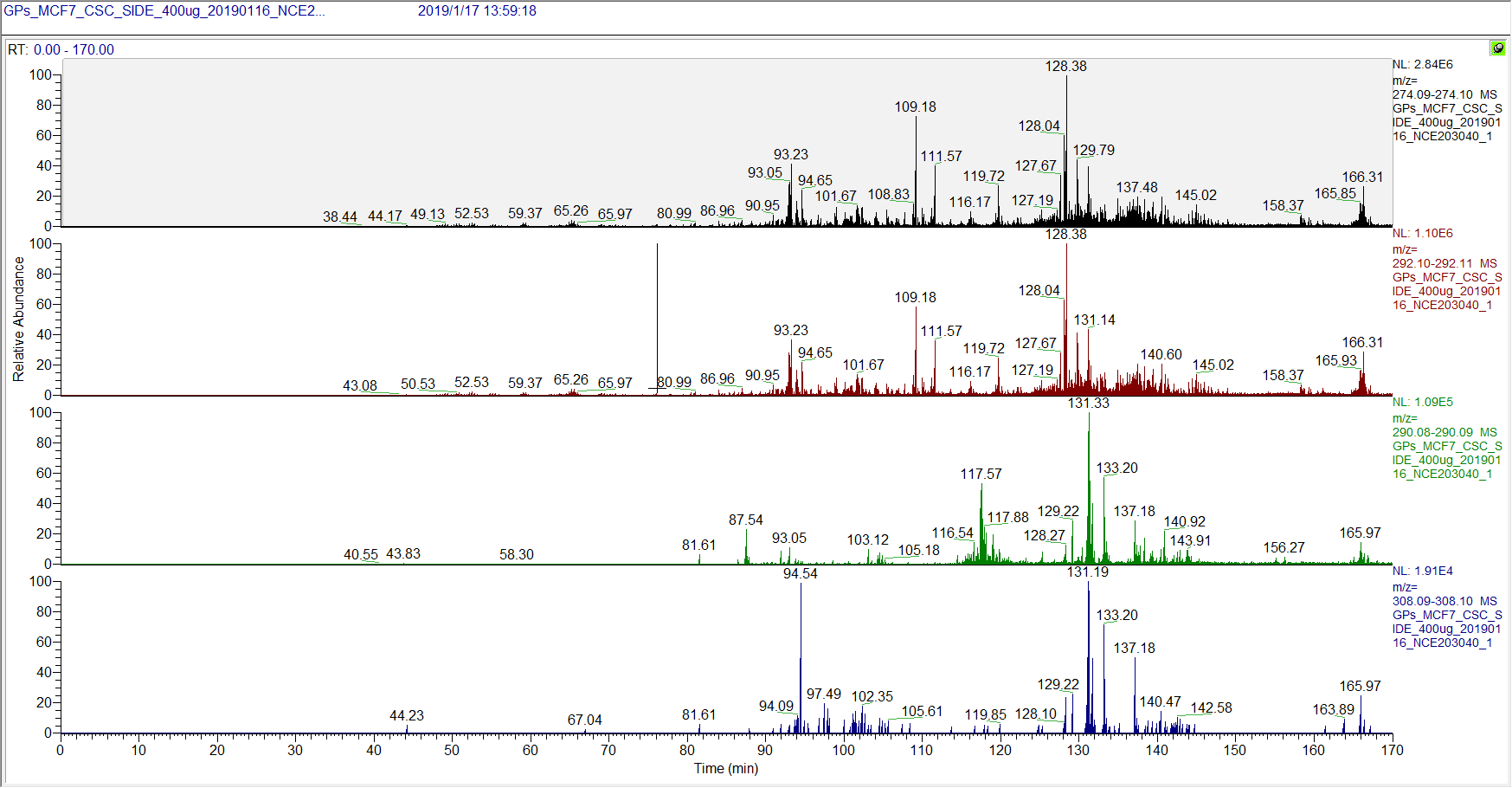


**Supplemental Figure 2.** Extract ion chromatograms (from top to bottom) of the characteristic oxonium ions of Neu5Ac-H_2_O, Neu5Ac, Neu5Gc-H_2_O, Neu5Gc with a mass tolerance of 20 ppm in the first technical replicate RPLC-MS/MS analysis of the intact N-glycopeptide mixture of MCF-7 and MCF-7 CSCs.


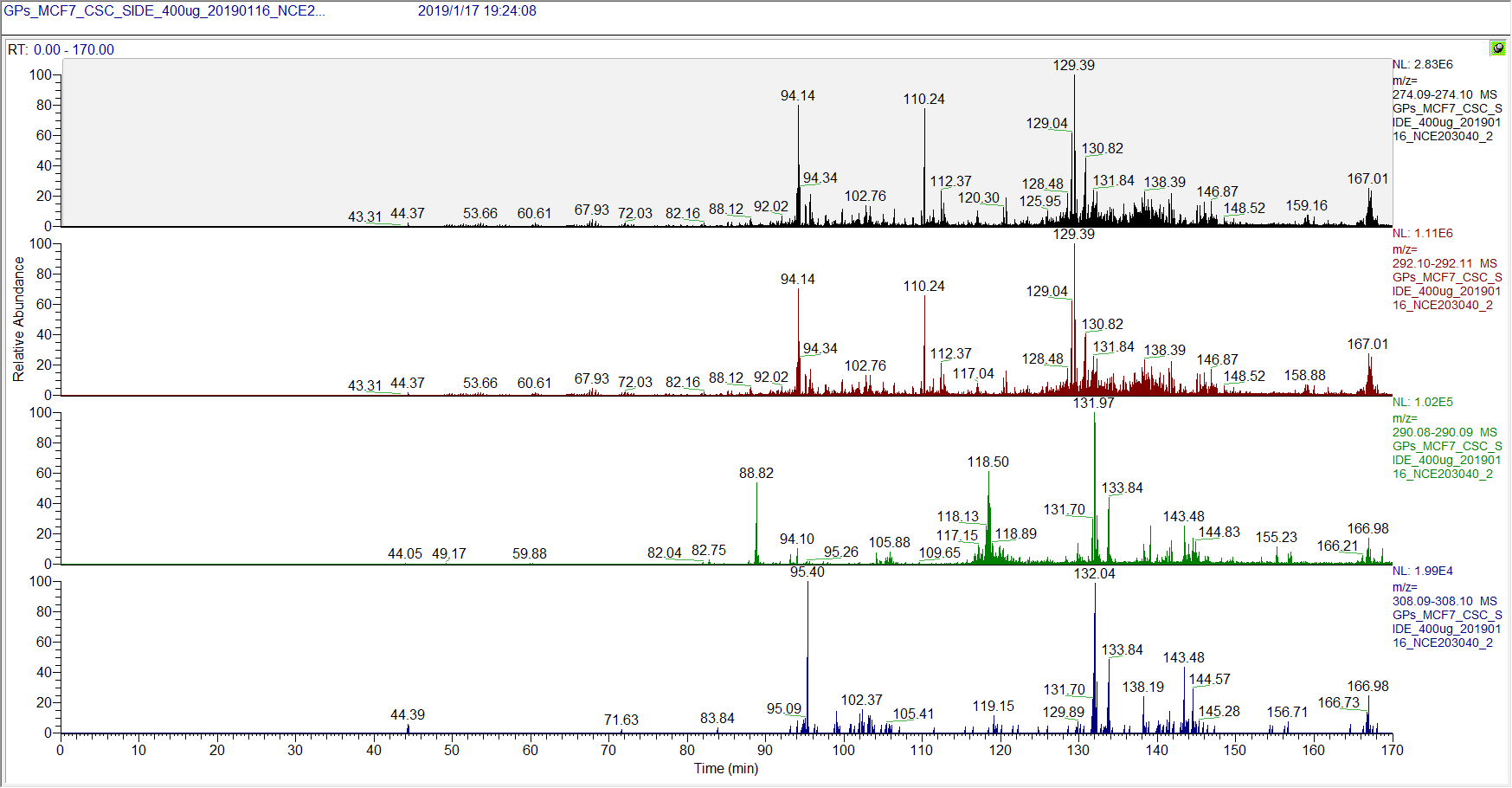


**Supplemental Figure 3.** Extract ion chromatograms of the characteristic oxonium ions of Neu5Ac-H2O, Neu5Ac, Neu5Gc-H2O, Neu5Gc with a mass tolerance of 20 ppm in the second technical replicate RPLC-MS/MS analysis of the intact N-glycopeptide mixture of MCF-7 and MCF-7 CSCs.


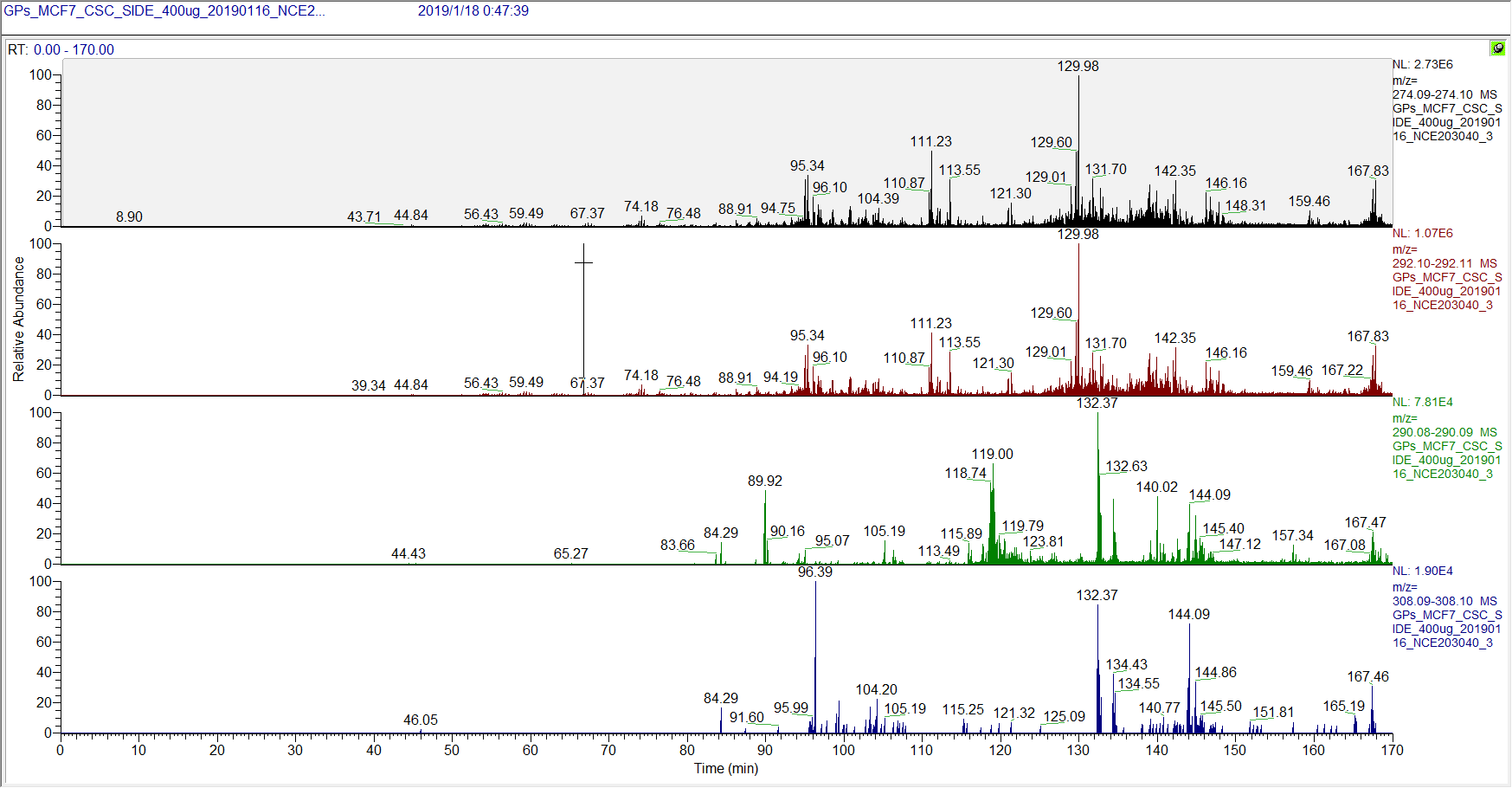


**Supplemental Figure 4.** Extract ion chromatograms of the characteristic oxonium ions of Neu5Ac-H2O, Neu5Ac, Neu5Gc-H2O, Neu5Gc with a mass tolerance of 20 ppm in the third technical replicate RPLC-MS/MS analysis of the intact N-glycopeptide mixture of MCF-7 and MCF-7 CSCs.


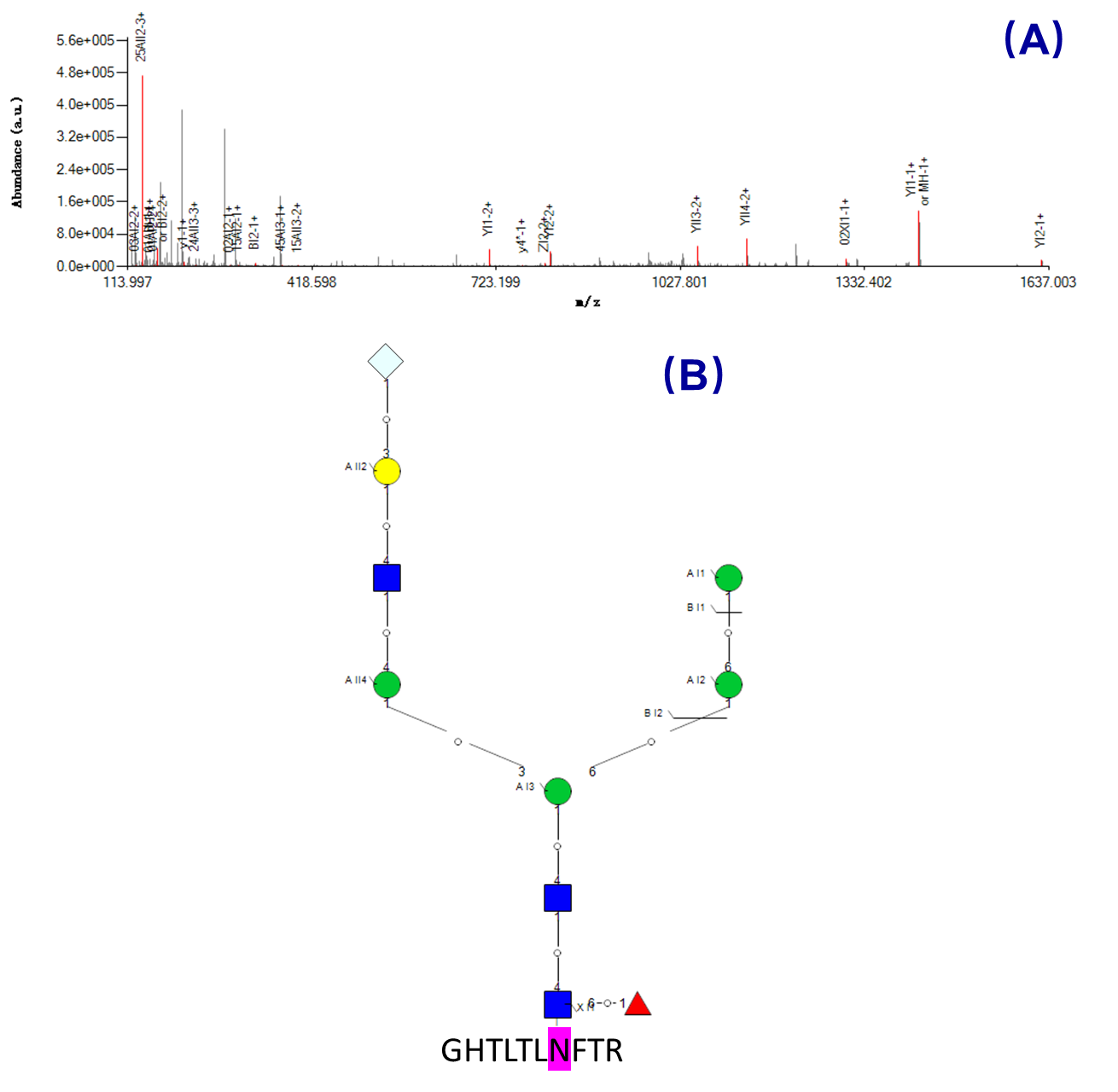


**Supplemental Figure 5.** Example intact N-glycopeptide GHTLTLNFTR with a hybrid N-glycan 01Y(61F)41Y41M(31M41Y41L32T)61M61M containing Neu5Gc identified from N-glycosite N103 of Lysosome-associated membrane glycoprotein 1 (LAMP1_HUMAN, P11279);
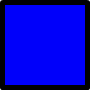
=N-acetylglucosamine (Y),
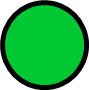
=mannose (M),
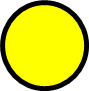
 =galactose (L),
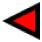
=fucose (F),
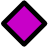
=N-acetylneuraminic acid(S),
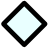
=N-glycolylneuraminic acid (T); a.u.=arbitrary unit.


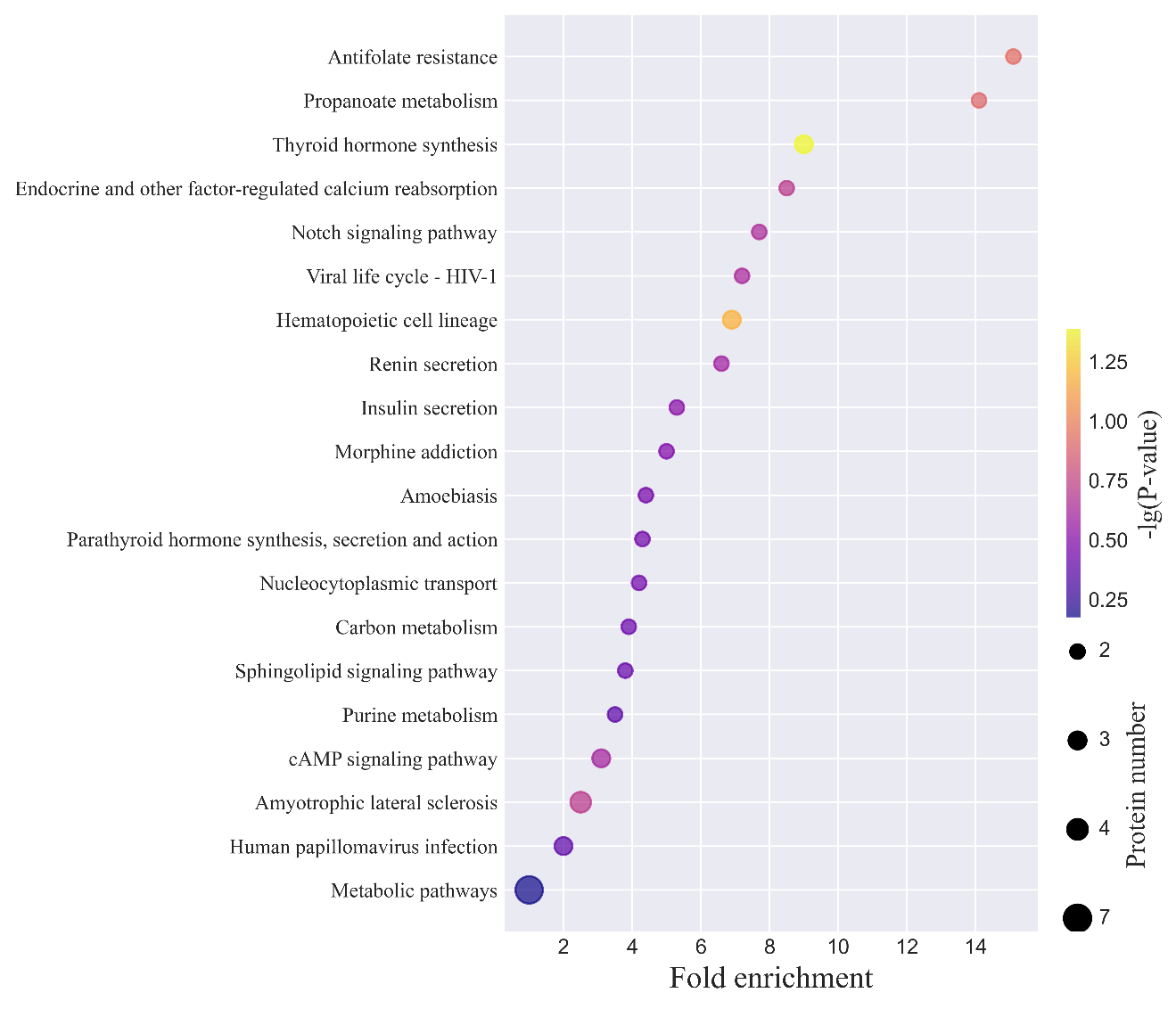


**Supplemental Figure 6.** Top20 KEGG pathways of N-glycoproteins corresponding to intact N-glycopeptides with Neu5Gc identified from MCF-7 CSCs.


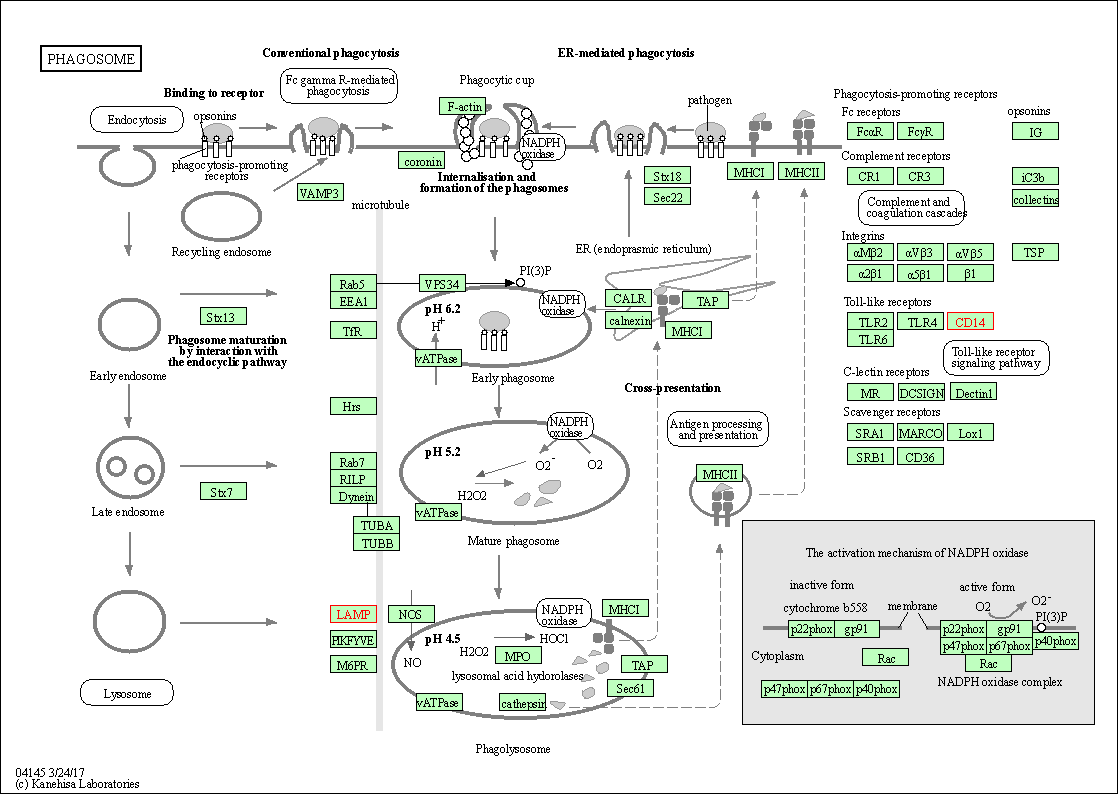


**Supplemental Figure 7.** N-glycoproteins LAMP1 and CD14, identified with Neu5Gc in this study, are involved in the phagosome pathway.


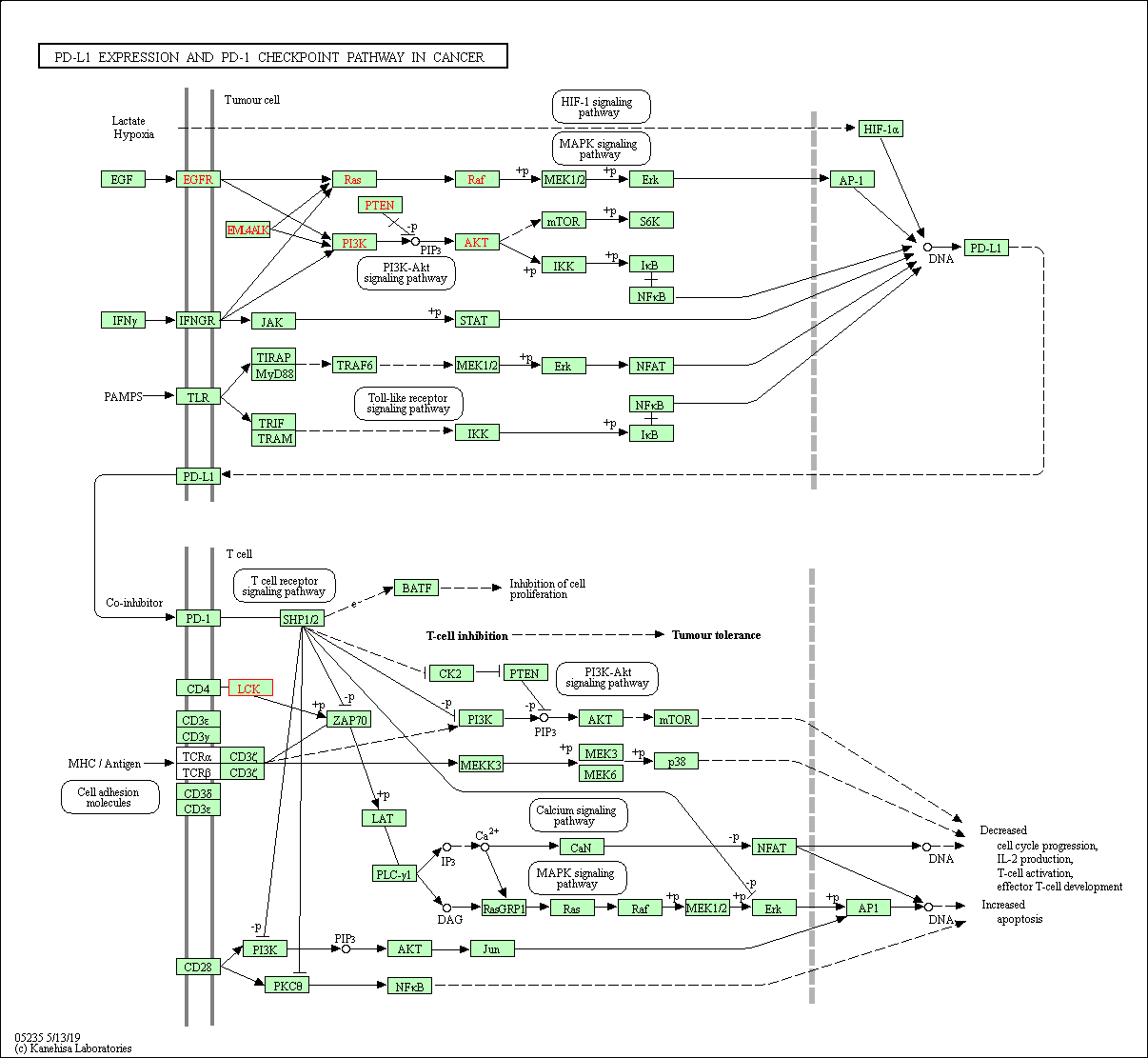


**Supplemental Figure 8.** N-glycoprotein LCK, identified with Neu5Gc in this study, are involved in the PD-L1 expression and PD-1 checkpoint pathway in cancer.


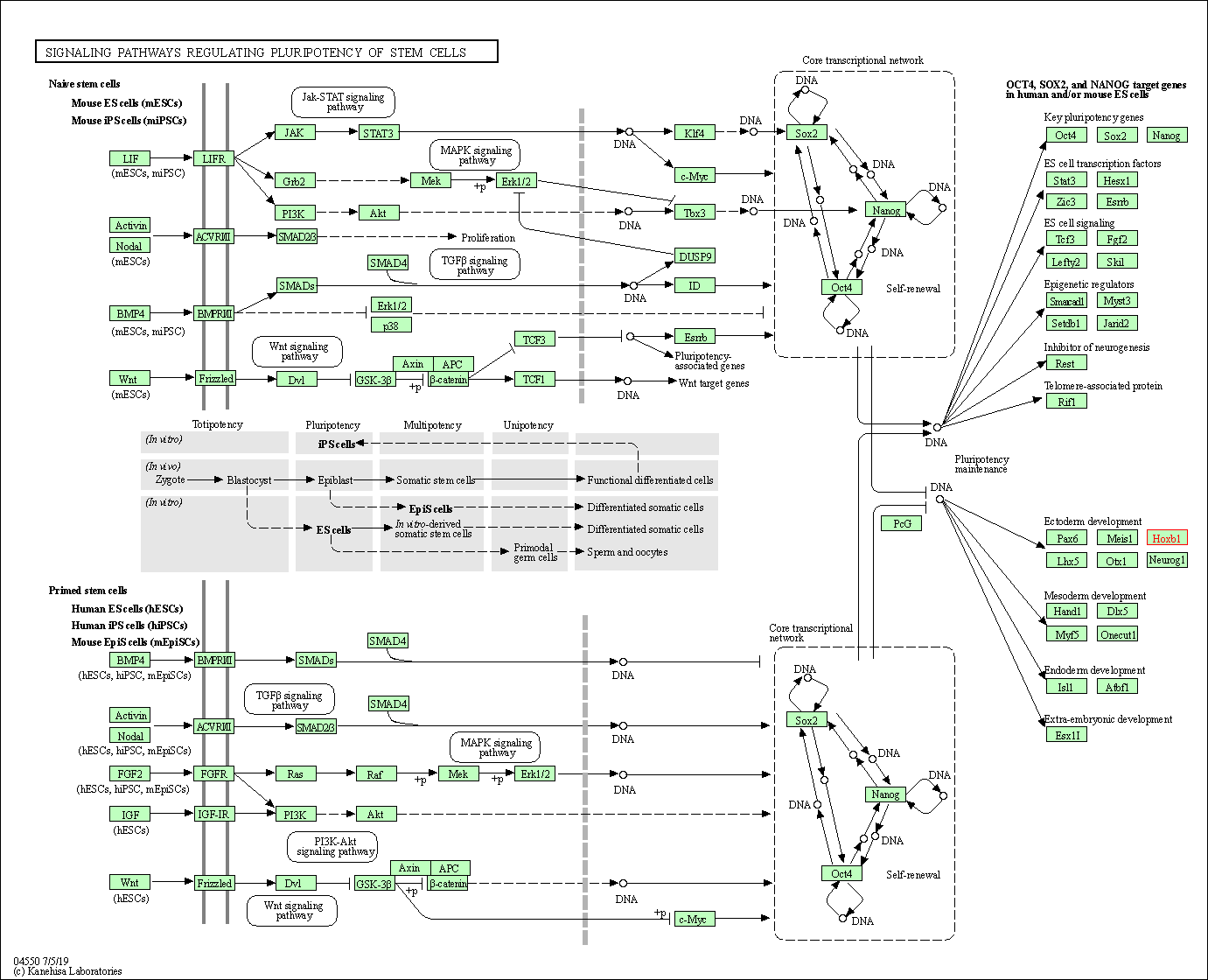


**Supplemental Figure 9.** N-glycoprotein HXB1, identified with Neu5Gc in this study, are involved in the signaling pathways regulating pluripotency of stem cells.


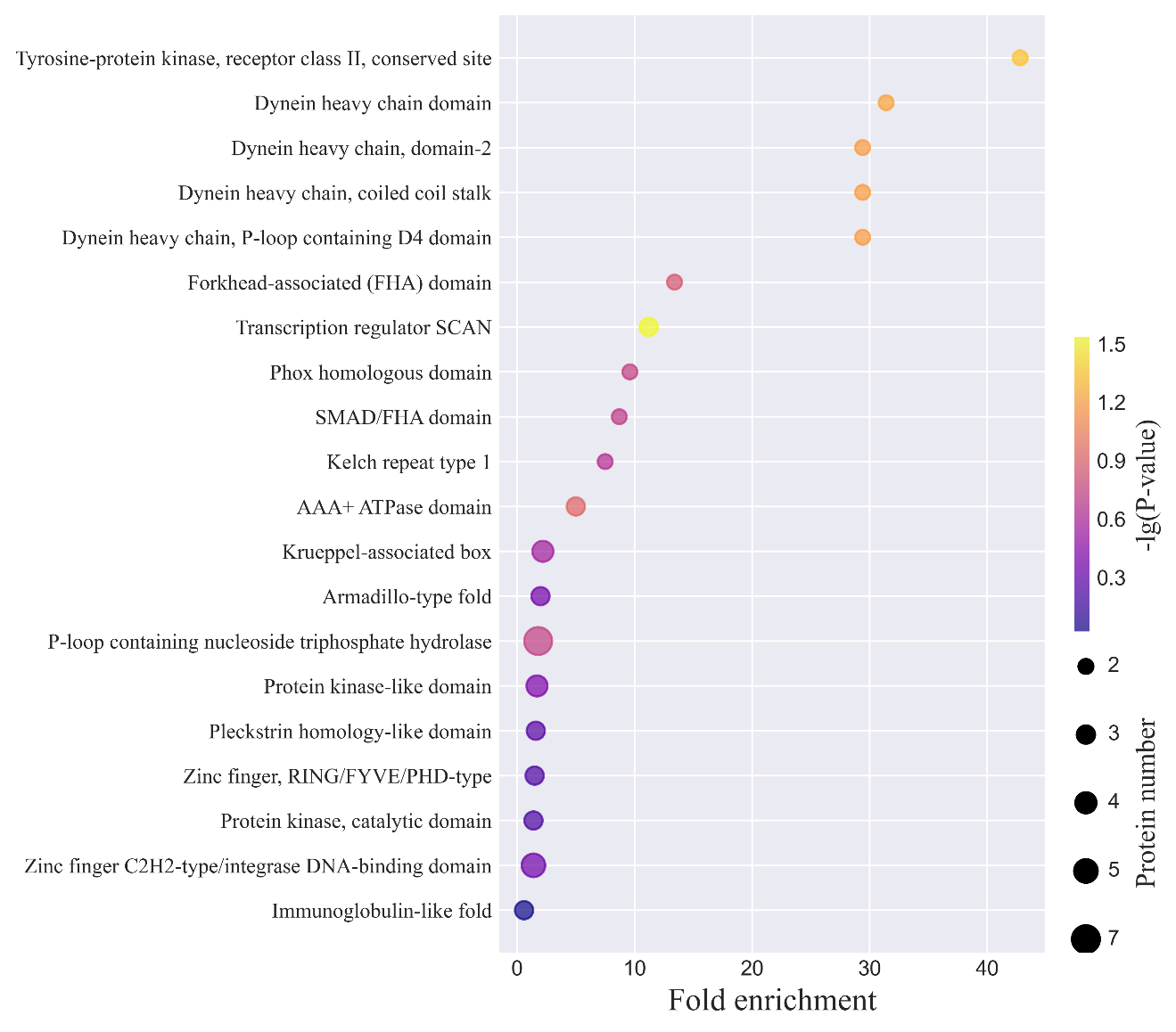


**Supplemental Figure 10.** Top20 INTERPRO domains of N-glycoproteins corresponding to intact N-glycopeptides with Neu5Gc identified from MCF-7 CSCs.


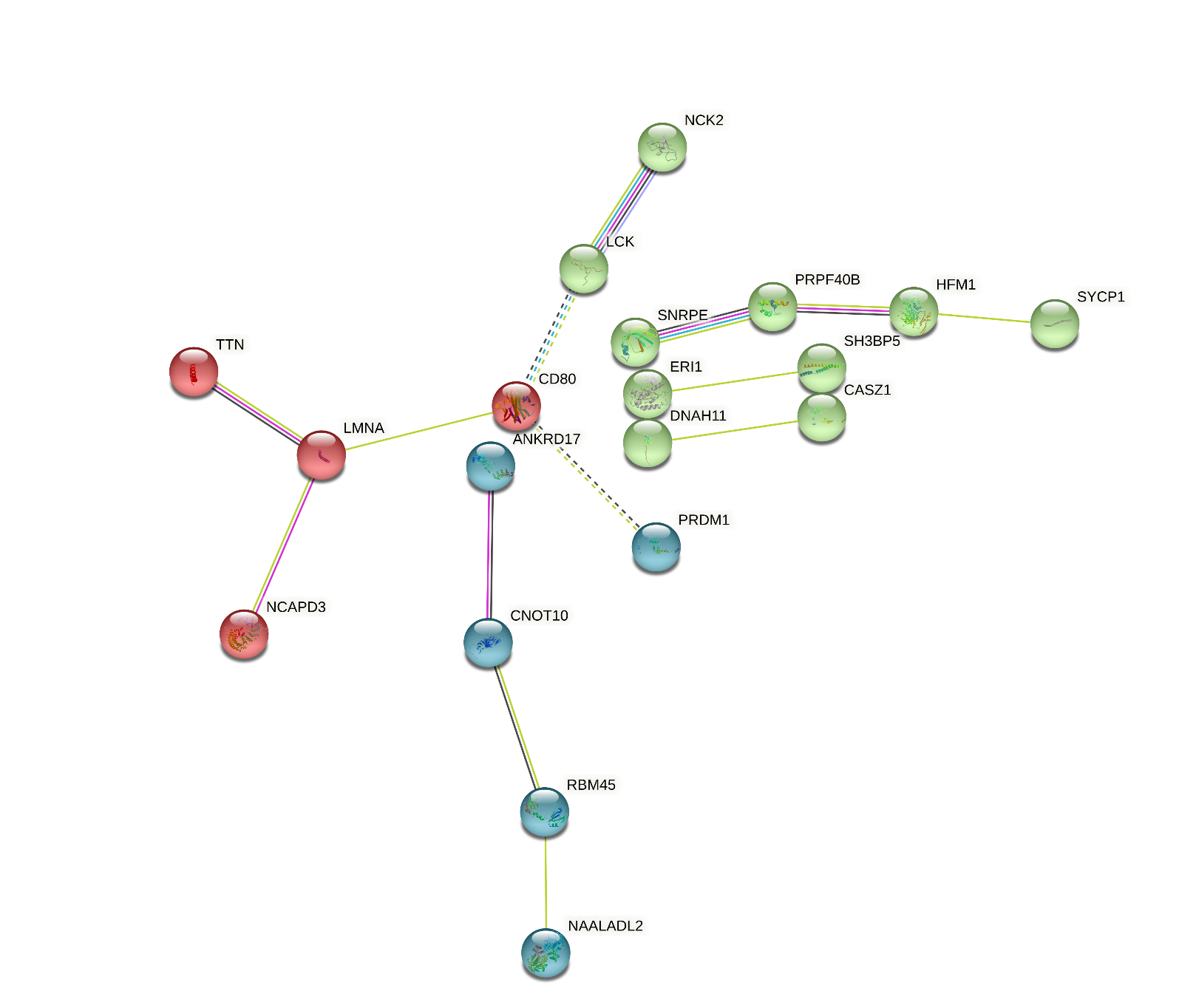


**Supplemental Figure 11.** PPI networks of the N-glycoproteins corresponding to the intact N-glycopeptides with Neu5Gc identified from MCF-7 cancer cells.


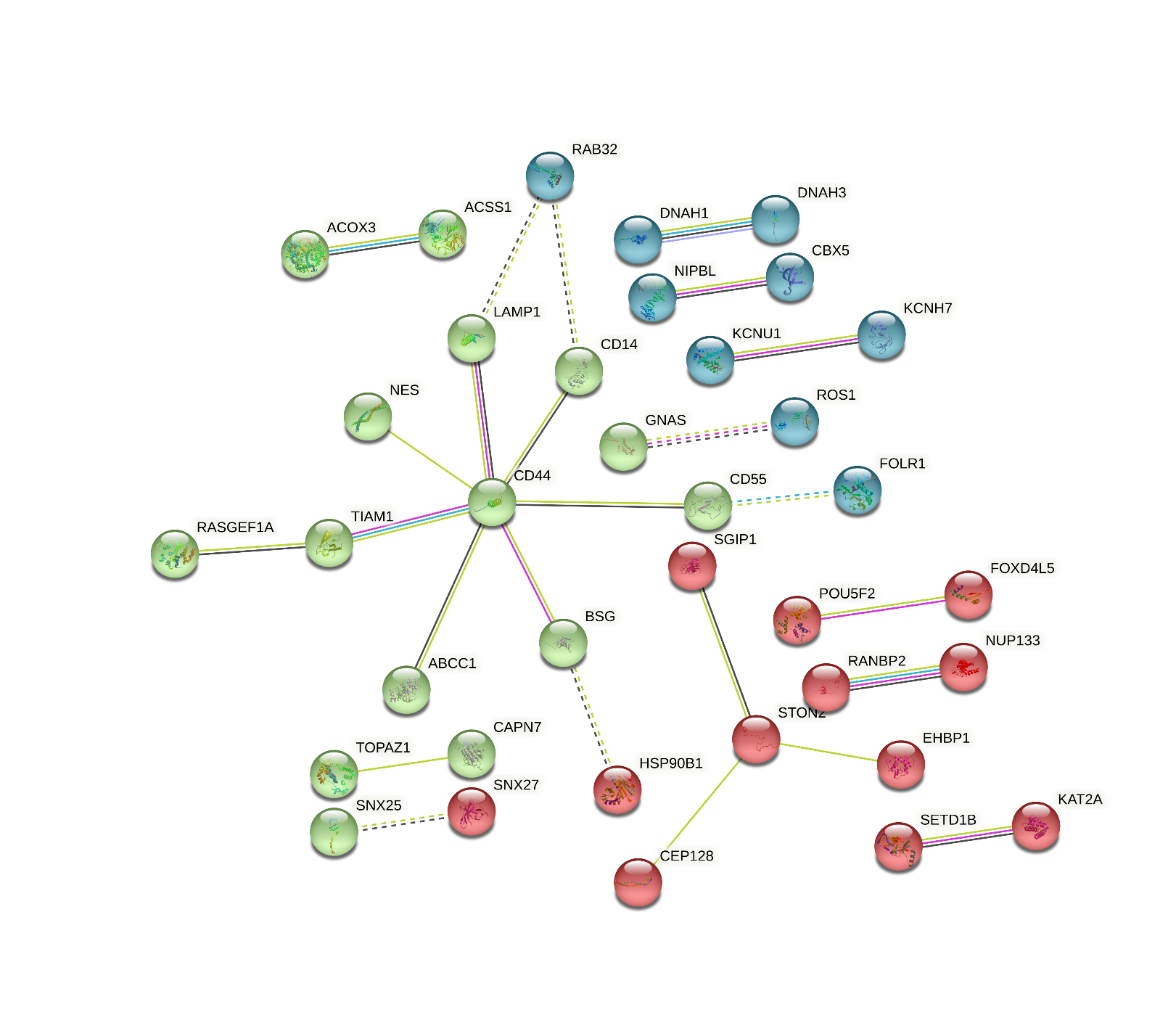


**Supplemental Figure 12.** PPI networks of the N-glycoproteins corresponding to the intact N-glycopeptides with Neu5Gc identified from MCF-7 CSCs.

**Supplemental Table 1.** The detailed information for the intact N-glycopeptides with Neu5Gc identified from breast MCF-7 cancer cells; AC=accession number; in N-glycan composition, N=N-acetylglucosamine, H=Hexose, F=fucose, S= N-acetylneuraminic acid, T=N-glycolylneuraminic acid; in N-glycan linkage, Y=N-acetylglucosamine, M=mannose, F=fucose, L=galactose, S= N-acetylneuraminic acid, T=N-glycolylneuraminic acid.

| AC | Site | peptide | Composition | N-glycan Linkage |
| --- | --- | --- | --- | --- |
| A2PYH4 | 1390 | NPNSSNYKK | N3H5F1S0T1 | 01Y41Y41M(31M21Y(31F)41L32T)61M61M |
| A6NNF4 | 211 | TFNWSSTLTNHK | N5H5F1S1T1 | 01Y41Y41M(31M41Y)61M(21Y(31F)41L32T)61Y41L32S |
| A6NNF4 | 211 | TFNWSSTLTNHK | N5H5F1S1T1 | 01Y41Y41M(31M41Y41L32T)61M(21Y41L32S)61Y31F |
| A6NNF4 | 211 | TFNWSSTLTNHK | N5H5F1S1T1 | 01Y(61F)41Y41M(31M41Y41L32S)(41Y)61M61Y41L32T |
| A6NNF4 | 211 | TFNWSSTLTNHK | N5H5F1S1T1 | 01Y(61F)41Y41M(31M41Y41L32T)(41Y)61M61Y41L32S |
| A8MT19 | 523 | TYSMICLAIDDDNK | N4H5F1S1T1 | 01Y41Y41M(31M)61M(21Y(31F)41L32T)61Y41L32S |
| A8MT19 | 523 | TYSMICLAIDDDNK | N4H5F1S1T1 | 01Y41Y41M(31M)61M(21Y(31F)41L32S)61Y41L32T |
| O14718 | 8 | MLRNNLGNSSDSK | N5H5F1S1T1 | 01Y41Y41M(31M41Y)61M(21Y(31F)41L32T)61Y41L32S |
| O14718 | 8 | MLRNNLGNSSDSK | N5H5F1S1T1 | 01Y41Y41M(31M41Y41L32T)61M(21Y41L32S)61Y31F |
| O15350 | 316 | EQQALNESSAK | N4H4F1S0T1 | 01Y41Y41M(31M)61M(21Y(31F)41L32T)61Y |
| O15350 | 316 | EQQALNESSAK | N4H4F1S0T1 | 01Y41Y41M(31M41Y)61M21Y(31F)41L32T |
| O43639 | 46 | NAANRTGYVPSNYVER | N7H7F1S1T1 | 01Y41Y41M(31M(21Y41L31Y41L)41Y31F)61M(21Y41L32T)61Y41L32S |
| O43639 | 46 | NAANRTGYVPSNYVER | N7H7F1S1T1 | 01Y41Y41M(31M(21Y41L31Y41L)41Y31F)61M(21Y41L32S)61Y41L32T |
| O43639 | 46 | NAANRTGYVPSNYVER | N7H7F1S1T1 | 01Y(61F)41Y41M(31M41Y41L31Y41L)(41Y)61M(21Y41L32T)61Y41L32S |
| O43639 | 46 | NAANRTGYVPSNYVER | N7H7F1S1T1 | 01Y(61F)41Y41M(31M41Y41L31Y41L)(41Y)61M(21Y41L32S)61Y41L32T |
| O60239 | 392 | AEGAENKTSDK | N4H4F1S0T1 | 01Y41Y41M(31M41Y)61M21Y(31F)41L32T |
| O60359 | 293 | DHAFLQFHNSTPK | N5H5F4S1T1 | 01Y(61F)41Y41M(31M41Y31F)61M(21Y(31F)41L32T)61Y(31F)41L32S |
| O60359 | 293 | DHAFLQFHNSTPK | N5H5F4S1T1 | 01Y(61F)41Y41M(31M41Y31F)61M(21Y(31F)41L32S)61Y(31F)41L32T |
| O75179 | 657 | GANVNR | N5H6F4S0T1 | 01Y(61F)41Y41M(31M21Y(31F)41L21F)61M(21Y(31F)41L32T)61Y41L |
| O75179 | 657 | GANVNR | N5H6F4S0T1 | 01Y(61F)41Y41M(31M(21Y(31F)41L21F)41Y41L)61M21Y(31F)41L32T |
| O75626 | 20 | CNSSTVRFQGLAEGTK | N7H7F1S1T1 | 01Y41Y41M(31M(21Y41L31Y41L)41Y31F)61M(21Y41L32T)61Y41L32S |
| O75626 | 20 | CNSSTVRFQGLAEGTK | N7H7F1S1T1 | 01Y41Y41M(31M(21Y41L31Y41L)41Y31F)61M(21Y41L32S)61Y41L32T |
| O75626 | 20 | CNSSTVRFQGLAEGTK | N7H7F1S1T1 | 01Y(61F)41Y41M(31M41Y41L31Y41L)(41Y)61M(21Y41L32T)61Y41L32S |
| O75626 | 20 | CNSSTVRFQGLAEGTK | N7H7F1S1T1 | 01Y(61F)41Y41M(31M41Y41L31Y41L)(41Y)61M(21Y41L32S)61Y41L32T |
| O95780 | 323 | AFNHCSLLTIHER | N5H5F4S1T1 | 01Y(61F)41Y41M(31M41Y31F)61M(21Y(31F)41L32T)61Y(31F)41L32S |
| O95780 | 323 | AFNHCSLLTIHER | N5H5F4S1T1 | 01Y(61F)41Y41M(31M41Y31F)61M(21Y(31F)41L32S)61Y(31F)41L32T |
| P02545 | 532 | TALINSTGEEVAMR | N5H5F1S1T1 | 01Y(61F)41Y41M(31M41Y41L32S)(41Y)61M61Y41L32T |
| P02545 | 532 | TALINSTGEEVAMR | N5H5F1S1T1 | 01Y(61F)41Y41M(31M41Y41L32T)(41Y)61M61Y41L32S |
| P05198 | 217 | AGLNCSTENMPIK | N5H5F3S0T1 | 01Y41Y41M(31M)61M(21Y(31F)41L31Y(31F)41L32T)61Y31F |
| P05198 | 217 | AGLNCSTENMPIK | N5H5F3S0T1 | 01Y41Y41M(31M(21Y(31F)41L21F)41Y31F)61M61Y41L32T |
| P06239 | 131 | ANSLEPEPWFFKNLSR | N5H5F2S1T1 | 01Y(61F)41Y41M(31M41Y)61M(21Y(31F)41L32T)61Y41L32S |
| P0C6C1 | 409 | LNSSHLSLFHGSR | N9H6F4S0T1 | 01Y(61F)41Y41M(31M(21Y(31F)41L31Y41V)41Y(31F)41L31Y)61M(21Y41L32T)61Y31F |
| P0C6C1 | 409 | LNSSHLSLFHGSR | N9H6F4S0T1 | 01Y(61F)41Y41M(31M(21Y(31F)41L31Y)41Y(31F)41L31Y)(41Y)61M(21Y41L32T)61Y31F |
| P28039 | 336 | NHCNHRDYQNISR | N4H5F1S1T1 | 01Y41Y41M(31M)61M(21Y(31F)41L32T)61Y41L32S |
| P28039 | 336 | NHCNHRDYQNISR | N4H5F1S1T1 | 01Y41Y41M(31M21Y(31F)41L32S)61M61Y41L32T |
| P28039 | 336 | NHCNHRDYQNISR | N4H5F3S0T1 | 01Y(61F)41Y41M(31M(21Y(31F)41L32T)41Y31F)61M61M |
| P28039 | 336 | NHCNHRDYQNISR | N4H5F3S0T1 | 01Y(61F)41Y41M(31M)61M(21Y(31F)41L32T)61Y(31F)41L |
| P28698 | 667 | QHANLTQHRR | N4H4F1S0T1 | 01Y41Y41M(31M)61M(21Y(31F)41L32T)61Y |
| P28698 | 667 | QHANLTQHRR | N4H4F1S0T1 | 01Y41Y41M(31M41Y)61M21Y(31F)41L32T |
| P33681 | 232 | VNQTFNWNTTK | N6H6F0S1T1 | 01Y41Y41M(31M41Y41L)(41Y)61M(21Y41L32T)61Y41L32S |
| P33681 | 232 | VNQTFNWNTTK | N6H6F0S1T1 | 01Y41Y41M(31M41Y41L)(41Y)61M(21Y41L32S)61Y41L32T |
| P33681 | 226 | VNQTFNWNTTK | N6H6F0S1T1 | 01Y41Y41M(31M41Y41L)(41Y)61M(21Y41L32T)61Y41L32S |
| P33681 | 226 | VNQTFNWNTTK | N6H6F0S1T1 | 01Y41Y41M(31M41Y41L)(41Y)61M(21Y41L32S)61Y41L32T |
| P33681 | 232 | VNQTFNWNTTK | N6H6F0S1T1 | 01Y41Y41M(31M41Y41L31Y)61M(21Y41L32T)61Y41L32S |
| P33681 | 232 | VNQTFNWNTTK | N6H6F0S1T1 | 01Y41Y41M(31M41Y41L31Y)61M(21Y41L32S)61Y41L32T |
| P33681 | 226 | VNQTFNWNTTK | N6H6F0S1T1 | 01Y41Y41M(31M41Y41L31Y)61M(21Y41L32T)61Y41L32S |
| P33681 | 226 | VNQTFNWNTTK | N6H6F0S1T1 | 01Y41Y41M(31M41Y41L31Y)61M(21Y41L32S)61Y41L32T |
| P35367 | 228 | QHCQHRELINR | N5H5F3S0T1 | 01Y41Y41M(31M)61M(21Y(31F)41L31Y(31F)41L32T)61Y31F |
| P35367 | 228 | QHCQHRELINR | N5H5F3S0T1 | 01Y41Y41M(31M(21Y(31F)41L21F)41Y31F)61M61Y41L32T |
| P41225 | 7 | ENSSGARSPR | N4H4F1S0T1 | 01Y41Y41M(31M)61M(21Y(31F)41L32T)61Y |
| P42695 | 506 | NSSAFSYQR | N4H4F1S0T1 | 01Y41Y41M(31M)61M(21Y(31F)41L32T)61Y |
| P42695 | 506 | NSSAFSYQR | N4H4F1S0T1 | 01Y41Y41M(31M41Y)61M21Y(31F)41L32T |
| P51522 | 160 | VFHNMSHLAQHRR | N5H5F4S1T1 | 01Y(61F)41Y41M(31M41Y31F)61M(21Y(31F)41L32T)61Y(31F)41L32S |
| P51522 | 160 | VFHNMSHLAQHRR | N5H5F4S1T1 | 01Y(61F)41Y41M(31M41Y31F)61M(21Y(31F)41L32S)61Y(31F)41L32T |
| P51522 | 160 | VFHNMSHLAQHRR | N5H5F4S1T1 | 01Y(61F)41Y41M(31M21Y(31F)41L32T)61M(21Y(31F)41L32S)61Y31F |
| P51522 | 160 | VFHNMSHLAQHRR | N5H5F4S1T1 | 01Y(61F)41Y41M(31M21Y(31F)41L32S)61M(21Y(31F)41L32T)61Y31F |
| P56975 | 563 | NTSINMQLPSR | N4H4F1S0T1 | 01Y41Y41M(31M)61M(21Y(31F)41L32T)61Y |
| P56975 | 563 | NTSINMQLPSR | N4H4F1S0T1 | 01Y41Y41M(31M41Y)61M21Y(31F)41L32T |
| P62304 | 83 | GDNITLLQSVSN | N4H4F1S0T1 | 01Y41Y41M(31M)61M(21Y(31F)41L32T)61Y |
| P62304 | 83 | GDNITLLQSVSN | N4H4F1S0T1 | 01Y41Y41M(31M41Y)61M21Y(31F)41L32T |
| P81877 | 324 | NSPNNMSLSNQPGTPR | N4H5F1S1T1 | 01Y41Y41M(31M)61M(21Y(31F)41L32T)61Y41L32S |
| P81877 | 324 | NSPNNMSLSNQPGTPR | N4H5F1S1T1 | 01Y41Y41M(31M)61M(21Y(31F)41L32S)61Y41L32T |
| P82279 | 37 | CLSNSCQNNSTCK | N7H7F0S0T1 | 01Y41Y41M(31M(21Y41L31Y41L)41Y41L31Y)61M61Y41L32T |
| P82279 | 41 | CLSNSCQNNSTCK | N7H7F0S0T1 | 01Y41Y41M(31M(21Y41L31Y41L)41Y41L31Y)61M61Y41L32T |
| P82279 | 42 | CLSNSCQNNSTCK | N7H7F0S0T1 | 01Y41Y41M(31M(21Y41L31Y41L)41Y41L31Y)61M61Y41L32T |
| P82279 | 37 | CLSNSCQNNSTCK | N7H7F0S0T1 | 01Y41Y41M(31M41Y41L31Y41L)(41Y)61M(21Y41L32T)61Y41L |
| P82279 | 41 | CLSNSCQNNSTCK | N7H7F0S0T1 | 01Y41Y41M(31M41Y41L31Y41L)(41Y)61M(21Y41L32T)61Y41L |
| P82279 | 42 | CLSNSCQNNSTCK | N7H7F0S0T1 | 01Y41Y41M(31M41Y41L31Y41L)(41Y)61M(21Y41L32T)61Y41L |
| Q03924 | 347 | AFNQLSNLTR | N3H5F1S0T1 | 01Y41Y41M(31M21Y(31F)41L32T)61M61M |
| Q13166 | 60 | NLCVSQSR | N6H5F2S0T1 | 01Y(61F)41Y41M(31M(21Y41L)41Y)61M(21Y41L32T)61Y31F |
| Q13166 | 60 | NLCVSQSR | N6H5F2S0T1 | 01Y41Y41M(31M(21Y41L)41Y)61M(21Y(31F)41L32T)61Y31F |
| Q13166 | 60 | NLCVSQSR | N6H5F2S0T1 | 01Y(61F)41Y41M(31M41Y41L31Y)61M(21Y41L32T)61Y31F |
| Q13166 | 60 | NLCVSQSR | N6H5F2S0T1 | 01Y41Y41M(31M41Y41L31Y)61M(21Y(31F)41L32T)61Y31F |
| Q14005 | 765 | LDTANGTPKVYK | N4H7F2S1T1 | 01Y(61F)41Y41M(31M(21Y(31F)41L32S)41Y41L32T)61M(31M)61M |
| Q14DG7 | 986 | NFLLNGSSQK | N3H5F1S0T1 | 01Y41Y41M(31M21Y(31F)41L32T)61M61M |
| Q15154 | 975 | TRQQNISMQR | N4H4F1S0T1 | 01Y41Y41M(31M41Y)61M21Y(31F)41L32T |
| Q15431 | 823 | NFTSVDHGISK | N4H4F1S0T1 | 01Y41Y41M(31M41Y)61M21Y(31F)41L32T |
| Q15431 | 823 | NFTSVDHGISK | N4H4F1S0T1 | 01Y41Y41M(31M)(41Y)61M21Y(31F)41L32T |
| Q3KNS6 | 394 | AFNKGSNLTR | N3H5F1S0T1 | 01Y41Y41M(31M21Y(31F)41L32T)61M61M |
| Q3L8U1 | 1644 | QEVIGNECQK | N4H4F1S0T1 | 01Y41Y41M(31M)61M(21Y(31F)41L32T)61Y |
| Q3L8U1 | 1644 | QEVIGNECQK | N4H4F1S0T1 | 01Y41Y41M(31M41Y)61M21Y(31F)41L32T |
| Q3L8U1 | 1644 | QEVIGNECQK | N4H4F1S0T1 | 01Y41Y41M(31M)61M(21Y41L32T)61Y31F |
| Q58DX5 | 121 | SNRCNFCHVLK | N5H5F3S0T1 | 01Y41Y41M(31M)61M(21Y(31F)41L31Y(31F)41L32T)61Y31F |
| Q58DX5 | 118 | SNRCNFCHVLK | N5H5F3S0T1 | 01Y41Y41M(31M)61M(21Y(31F)41L31Y(31F)41L32T)61Y31F |
| Q58DX5 | 121 | SNRCNFCHVLK | N5H5F3S0T1 | 01Y41Y41M(31M(21Y(31F)41L21F)41Y31F)61M61Y41L32T |
| Q58DX5 | 118 | SNRCNFCHVLK | N5H5F3S0T1 | 01Y41Y41M(31M(21Y(31F)41L21F)41Y31F)61M61Y41L32T |
| Q5T7B8 | 210 | QNTSEKQNPWTEMEK | N5H5F0S1T1 | 01Y41Y41M(31M41Y)61M(21Y41L32S)61Y41L32T |
| Q5T7B8 | 210 | QNTSEKQNPWTEMEK | N5H5F0S1T1 | 01Y41Y41M(31M41Y)61M(21Y41L32T)61Y41L32S |
| Q6NW40 | 413 | ERWHIFPSSGNGTPR | N5H5F3S1T1 | 01Y(61F)41Y41M(31M41Y)61M(21Y(31F)41L32T)61Y(31F)41L32S |
| Q6NW40 | 413 | ERWHIFPSSGNGTPR | N5H5F3S1T1 | 01Y(61F)41Y41M(31M41Y)61M(21Y(31F)41L32S)61Y(31F)41L32T |
| Q6NW40 | 413 | ERWHIFPSSGNGTPR | N5H5F3S1T1 | 01Y(61F)41Y41M(31M41Y31F)61M(21Y(31F)41L32T)61Y41L32S |
| Q6NWY9 | 154 | SDTGKPYYYNNQSKESR | N4H4F1S0T1 | 01Y41Y41M(31M41Y)61M21Y(31F)41L32T |
| Q6NWY9 | 154 | SDTGKPYYYNNQSKESR | N4H4F1S0T1 | 01Y(61F)41Y41M(31M41Y)61M61Y41L32T |
| Q6YI46 | 358 | GNQPNTSGSSFYNKR | N5H5F3S1T1 | 01Y41Y41M(31M41Y31F)61M(21Y(31F)41L32S)61Y(31F)41L32T |
| Q6YI46 | 358 | GNQPNTSGSSFYNKR | N5H5F3S1T1 | 01Y41Y41M(31M41Y31F)61M(21Y(31F)41L32T)61Y(31F)41L32S |
| Q6YI46 | 358 | GNQPNTSGSSFYNKR | N5H5F3S1T1 | 01Y(61F)41Y41M(31M41Y31F)61M(21Y(31F)41L32T)61Y41L32S |
| Q6ZMW2 | 537 | CNQCGKAFGQK | N8H8F2S1T1 | 01Y(61F)41Y41M(31M(21Y41L31Y41L)41Y(31F)41L31Y)61M(21Y41L32S)61Y41L32T |
| Q6ZMW2 | 537 | CNQCGKAFGQK | N8H8F2S1T1 | 01Y(61F)41Y41M(31M(21Y41L31Y41L)41Y(31F)41L31Y)61M(21Y41L32T)61Y41L32S |
| Q6ZMW2 | 537 | CNQCGKAFGQK | N8H8F2S1T1 | 01Y(61F)41Y41M(31M(21Y41L31Y41L)41Y41L31Y)61M(21Y(31F)41L32T)61Y41L32S |
| Q6ZMW2 | 537 | CNQCGKAFGQK | N8H8F2S1T1 | 01Y41Y41M(31M(21Y41L31Y41L)41Y(31F)41L31Y)61M(21Y(31F)41L32T)61Y41L32S |
| Q6ZRG5 | 5 | WNFSPEDLSSIFR | N5H5F4S1T1 | 01Y(61F)41Y41M(31M41Y31F)61M(21Y(31F)41L32T)61Y(31F)41L32S |
| Q6ZRG5 | 5 | WNFSPEDLSSIFR | N5H5F4S1T1 | 01Y(61F)41Y41M(31M41Y31F)61M(21Y(31F)41L32S)61Y(31F)41L32T |
| Q7Z569 | 494 | EEQEMNK | N6H5F2S0T1 | 01Y41Y41M(31M(21Y41L)41Y)61M(21Y(31F)41L32T)61Y31F |
| Q86V15 | 1254 | TGCYFVTNITTK | N8H7F2S1T1 | 01Y41Y41M(31M(21Y(31F)41L31Y)41Y(31F)41L31Y)61M(21Y41L32T)61Y41L32S |
| Q86V15 | 1254 | TGCYFVTNITTK | N8H7F2S1T1 | 01Y41Y41M(31M(21Y(31F)41L31Y)41Y(31F)41L31Y)61M(21Y41L32S)61Y41L32T |
| Q86V15 | 1254 | TGCYFVTNITTK | N8H7F2S1T1 | 01Y41Y41M(31M(21Y41L31Y31F)41Y(31F)41L31Y)61M(21Y41L32T)61Y41L32S |
| Q86V15 | 1254 | TGCYFVTNITTK | N8H7F2S1T1 | 01Y41Y41M(31M(21Y41L31Y31F)41Y(31F)41L31Y)61M(21Y41L32S)61Y41L32T |
| Q8IUH3 | 238 | NDSRGQEAISK | N4H4F1S0T1 | 01Y41Y41M(31M)61M(21Y(31F)41L32T)61Y |
| Q8IUH3 | 238 | NDSRGQEAISK | N4H4F1S0T1 | 01Y41Y41M(31M41Y)61M21Y(31F)41L32T |
| Q8IV48 | 73 | EIAITNGCINR | N4H4F1S0T1 | 01Y41Y41M(31M)61M(21Y(31F)41L32T)61Y |
| Q8IV48 | 73 | EIAITNGCINR | N4H4F1S0T1 | 01Y41Y41M(31M41Y)61M21Y(31F)41L32T |
| Q8IVL0 | 129 | VEDINGCPR | N4H4F1S0T1 | 01Y41Y41M(31M)61M(21Y(31F)41L32T)61Y |
| Q8IVL0 | 129 | VEDINGCPR | N4H4F1S0T1 | 01Y41Y41M(31M41Y)61M21Y(31F)41L32T |
| Q8IX21 | 211 | QTTVAEADIFNNSSR | N5H5F0S0T1 | 01Y41Y41M(31M(21Y41L)41Y)61M61Y41L32T |
| Q8IX21 | 211 | QTTVAEADIFNNSSR | N5H5F0S0T1 | 01Y41Y41M(31M41Y41L31Y)61M61Y41L32T |
| Q8NFM7 | 171 | NESNYHPFFFRTR | N4H5F1S1T1 | 01Y41Y41M(31M)61M(21Y(31F)41L32T)61Y41L32S |
| Q8NFM7 | 171 | NESNYHPFFFRTR | N4H5F1S1T1 | 01Y41Y41M(31M)61M(21Y(31F)41L32S)61Y41L32T |
| Q8WVD3 | 60 | ESGAHCPLCRGNVTR | N4H5F3S0T1 | 01Y(61F)41Y41M(31M(21Y(31F)41L32T)41Y31F)61M61M |
| Q8WVD3 | 60 | ESGAHCPLCRGNVTR | N4H5F3S0T1 | 01Y(61F)41Y41M(31M)61M(21Y(31F)41L32T)61Y(31F)41L |
| Q8WZ42 | 8834 | NGINVTPSQRCNITTTEK | N5H5F2S1T1 | 01Y(61F)41Y41M(31M41Y)61M(21Y(31F)41L32T)61Y41L32S |
| Q96DT5 | 1342 | SIDNWTKTQWR | N5H5F3S0T1 | 01Y41Y41M(31M)61M(21Y(31F)41L31Y(31F)41L32T)61Y31F |
| Q96F05 | 41 | RNASVETVDNK | N6H6F4S1T1 | 01Y(61F)41Y41M(31M21Y(31F)41L21F)(41Y)61M(21Y(31F)41L32S)61Y41L32T |
| Q96JK2 | 230 | YGGNLSLQSAMSVR | N10H8F2S0T1 | 01Y(61F)41Y41M(31M(21Y41L31Y41L)41Y(31F)41L31Y)(41Y)61M(21Y41L31Y41L32T)61Y |
| Q96JK2 | 230 | YGGNLSLQSAMSVR | N10H8F2S0T1 | 01Y41Y41M(31M(21Y(31F)41L31Y41L)41Y(31F)41L31Y)(41Y)61M(21Y41L31Y41L32T)61Y |
| Q96PY6 | 36 | QYVIKEINISR | N4H7F2S1T1 | 01Y(61F)41Y41M(31M(21Y(31F)41L32S)41Y41L32T)61M(31M)61M |
| Q96RT7 | 1112 | WNIHGHVSNASIR | N5H5F3S0T1 | 01Y41Y41M(31M)61M(21Y(31F)41L31Y(31F)41L32T)61Y31F |
| Q96RT7 | 1112 | WNIHGHVSNASIR | N5H5F3S0T1 | 01Y41Y41M(31M(21Y(31F)41L21F)41Y31F)61M61Y41L32T |
| Q96RT7 | 1112 | WNIHGHVSNASIR | N5H5F3S0T1 | 01Y(61F)41Y41M(31M41Y(31F)41L31Y)61M21Y(31F)41L32T |
| Q96RT7 | 1112 | WNIHGHVSNASIR | N5H5F3S0T1 | 01Y(61F)41Y41M(31M41Y41L)61M(21Y(31F)41L32T)61Y31F |
| Q9H7T3 | 38 | SGSAGAPSKNSSR | N4H4F1S0T1 | 01Y41Y41M(31M41Y)61M21Y(31F)41L32T |
| Q9H9A5 | 190 | NETGNNNNK | N4H4F1S0T1 | 01Y41Y41M(31M)61M(21Y(31F)41L32T)61Y |
| Q9HBM0 | 649 | VSKNDTEEESNK | N5H5F1S1T1 | 01Y(61F)41Y41M(31M41Y41L32S)(41Y)61M61Y41L32T |
| Q9HBM0 | 649 | VSKNDTEEESNK | N5H5F1S1T1 | 01Y(61F)41Y41M(31M41Y41L32T)(41Y)61M61Y41L32S |
| Q9HCS5 | 450 | NPSCGSDNDSVQPVR | N4H4F1S0T1 | 01Y41Y41M(31M)61M(21Y(31F)41L32T)61Y |
| Q9HCS5 | 450 | NPSCGSDNDSVQPVR | N4H4F1S0T1 | 01Y41Y41M(31M41Y)61M21Y(31F)41L32T |
| Q9NS84 | 88 | FPSNLSGAVGEAVSR | N5H5F3S0T1 | 01Y(61F)41Y41M(31M41Y(31F)41L31Y)61M21Y(31F)41L32T |
| Q9NZR2 | 2549 | SDEKLLYCENR | N10H8F2S0T1 | 01Y(61F)41Y41M(31M(21Y41L31Y41L)41Y(31F)41L31Y)(41Y)61M(21Y41L31Y41L32T)61Y |
| Q9NZR2 | 2549 | SDEKLLYCENR | N10H8F2S0T1 | 01Y41Y41M(31M(21Y(31F)41L31Y41L)41Y(31F)41L31Y)(41Y)61M(21Y41L31Y41L32T)61Y |
| Q9UK23 | 420 | TGNCSVSRVK | N3H5F1S0T1 | 01Y41Y41M(31M21Y(31F)41L32T)61M61M |
| Q9UKL3 | 319 | GERINSSWEK | N4H4F1S0T1 | 01Y41Y41M(31M41Y)61M21Y(31F)41L32T |
| Q9UMX1 | 265 | GIETDGSNLSGVSAK | N5H5F3S0T1 | 01Y41Y41M(31M)61M(21Y(31F)41L31Y(31F)41L32T)61Y31F |
| Q9Y227 | 404 | ETIQPFMNK | N3H5F1S0T1 | 01Y41Y41M(31M21Y(31F)41L32T)61M61M |
| Q9Y5Q0 | 396 | MPRHNYSR | N4H4F1S0T1 | 01Y41Y41M(31M)61M(21Y(31F)41L32T)61Y |

**Supplemental Table 2.** The detailed information for the intact N-glycopeptides with Neu5Gc identified from breast MCF-7 cancer stem cells; AC=accession number; in N-glycan composition, N=N-acetylglucosamine, H=Hexose, F=fucose, S= N-acetylneuraminic acid, T=N-glycolylneuraminic acid; in N-glycan linkage, Y=N-acetylglucosamine, M=mannose, F=fucose, L=galactose, S= N-acetylneuraminic acid, T=N-glycolylneuraminic acid.

| AC | Site | Peptide | Composition | N-glycan Linkage |
| --- | --- | --- | --- | --- |
| P11279 | 103 | GHTLTLNFTR | N3H5F1S0T1 | 01Y41Y41M(31M21Y(31F)41L32T)61M61M |
| P15328 | 167 | GWNWTSGFNK | N4H4F1S0T1 | 01Y41Y41M(31M)61M(21Y(31F)41L32T)61Y |
| P15328 | 161 | GWNWTSGFNK | N4H4F1S0T1 | 01Y41Y41M(31M)61M(21Y(31F)41L32T)61Y |
| Q6ZUT9 | 855 | HIQNMSEIK | N3H5F1S0T1 | 01Y41Y41M(31M21Y(31F)41L32T)61M61M |
| Q9NS40 | 369 | DRTHNVTEK | N3H5F1S0T1 | 01Y41Y41M(31M21Y(31F)41L32T)61M61M |
| Q9H061 | 11 | ENITIVDISR | N3H5F1S0T1 | 01Y41Y41M(31M21Y(31F)41L32T)61M61M |
| P35613 | 160 | ILLTCSLNDSATEVTGHR | N4H4F0S0T1 | 01Y41Y41M(31M41Y)61M61Y41L32T |
| P08571 | 151 | LRNVSWATGR | N3H5F1S0T1 | 01Y41Y41M(31M21Y(31F)41L32T)61M61M |
| P35613 | 160 | ILLTCSLNDSATEVTGHR | N4H4F0S0T1 | 01Y41Y41M(31M)61M(21Y41L32T)61Y |
| O15360 | 1140 | GLLNACLRSR | N3H5F1S0T1 | 01Y41Y41M(31M21Y(31F)41L32T)61M61M |
| Q6NXR4 | 444 | DPNLTPESVK | N3H5F1S0T1 | 01Y41Y41M(31M21Y(31F)41L32T)61M61M |
| Q16670 | 425 | CNICQKAFR | N4H4F1S0T1 | 01Y41Y41M(31M)61M(21Y(31F)41L32T)61Y |
| Q9Y3L5 | 165 | QMNYSSLPEK | N4H4F1S0T1 | 01Y41Y41M(31M)61M(21Y(31F)41L32T)61Y |
| P15328 | 167 | GWNWTSGFNK | N4H4F1S0T1 | 01Y41Y41M(31M41Y)61M21Y(31F)41L32T |
| P15328 | 161 | GWNWTSGFNK | N4H4F1S0T1 | 01Y41Y41M(31M41Y)61M21Y(31F)41L32T |
| Q6ZU80 | 296 | RLLNQSEGSR | N3H5F1S0T1 | 01Y41Y41M(31M21Y(31F)41L32T)61M61M |
| P51679 | 194 | YSLNSTTWK | N3H5F1S0T1 | 01Y41Y41M(31M21Y(31F)41L32T)61M61M |
| P16070 | 57 | AFNSTLPTMAQMEK | N5H5F4S1T1 | 01Y(61F)41Y41M(31M41Y31F)61M(21Y(31F)41L32T)61Y(31F)41L32S |
| P16070 | 57 | AFNSTLPTMAQMEK | N5H5F4S1T1 | 01Y(61F)41Y41M(31M41Y31F)61M(21Y(31F)41L32S)61Y(31F)41L32T |
| Q495M3 | 477 | SEDSHPFSNSTTFVR | N4H5F1S1T1 | 01Y41Y41M(31M)61M(21Y(31F)41L32T)61Y41L32S |
| P08174 | 95 | GSQWSDIEEFCNR | N4H4F1S0T1 | 01Y41Y41M(31M)61M(21Y(31F)41L32T)61Y |
| P16070 | 57 | AFNSTLPTMAQMEK | N5H5F4S1T1 | 01Y(61F)41Y41M(31M21Y(31F)41L32T)61M(21Y(31F)41L32S)61Y31F |
| P16070 | 57 | AFNSTLPTMAQMEK | N5H5F4S1T1 | 01Y(61F)41Y41M(31M21Y(31F)41L32S)61M(21Y(31F)41L32T)61Y31F |
| P33527 | 1297 | VEFRNYCLR | N4H4F1S0T1 | 01Y41Y41M(31M)61M(21Y(31F)41L32T)61Y |
| Q99538 | 167 | DLNETIHYMYK | N5H5F3S0T1 | 01Y41Y41M(31M)61M(21Y(31F)41L31Y(31F)41L32T)61Y31F |
| Q8N7G0 | 260 | VWFYNRSK | N3H5F1S0T1 | 01Y41Y41M(31M21Y(31F)41L32T)61M61M |
| Q495M3 | 477 | SEDSHPFSNSTTFVR | N4H5F1S1T1 | 01Y41Y41M(31M)61M(21Y(31F)41L32S)61Y41L32T |
| Q8WXE9 | 344 | SQNSSISSTTGK | N4H4F1S0T1 | 01Y41Y41M(31M)61M(21Y(31F)41L32T)61Y |
| Q96L96 | 1678 | NYDVTIQGCKIQNMSR | N4H4F0S0T1 | 01Y41Y41M(31M41Y)61M61Y41L32T |
| Q99538 | 167 | DLNETIHYMYK | N5H5F3S0T1 | 01Y41Y41M(31M(21Y(31F)41L21F)41Y31F)61M61Y41L32T |
| P33527 | 1297 | VEFRNYCLR | N4H4F1S0T1 | 01Y41Y41M(31M41Y)61M21Y(31F)41L32T |
| Q96L96 | 1678 | NYDVTIQGCKIQNMSR | N4H4F0S0T1 | 01Y41Y41M(31M)61M(21Y41L32T)61Y |
| Q499Y3 | 122 | STQASSGENSTK | N4H4F1S0T1 | 01Y41Y41M(31M)61M(21Y(31F)41L32T)61Y |
| P08174 | 95 | GSQWSDIEEFCNR | N4H4F3S0T1 | 01Y(61F)41Y41M(31M)61M(21Y(31F)41L32T)61Y31F |
| Q92830 | 115 | ANETCKCNGWK | N5H5F1S1T1 | 01Y41Y41M(31M41Y)61M(21Y(31F)41L32T)61Y41L32S |
| Q16670 | 425 | CNICQKAFR | N4H4F1S0T1 | 01Y41Y41M(31M41Y)61M21Y(31F)41L32T |
| P08174 | 95 | GSQWSDIEEFCNR | N4H4F1S0T1 | 01Y41Y41M(31M41Y)61M21Y(31F)41L32T |
| P14625 | 217 | HNNDTQHIWESDSNEFSVIADPR | N4H4F1S0T1 | 01Y41Y41M(31M41Y)61M21Y(31F)41L32T |
| Q9Y3L5 | 165 | QMNYSSLPEK | N4H4F1S0T1 | 01Y41Y41M(31M41Y)61M21Y(31F)41L32T |
| P08174 | 95 | GSQWSDIEEFCNR | N4H4F3S0T1 | 01Y(61F)41Y41M(31M41Y31F)61M21Y(31F)41L32T |
| P11279 | 84 | SSCGKENTSDPSLVIAFGR | N5H5F2S1T1 | 01Y(61F)41Y41M(31M41Y)61M(21Y(31F)41L32T)61Y41L32S |
| P35613 | 160 | ILLTCSLNDSATEVTGHR | N5H5F2S1T1 | 01Y41Y41M(31M41Y)61M(21Y(31F)41L32S)61Y(31F)41L32T |
| P35613 | 160 | ILLTCSLNDSATEVTGHR | N5H5F2S1T1 | 01Y41Y41M(31M41Y)61M(21Y(31F)41L32T)61Y(31F)41L32S |
| Q7Z3H0 | 211 | NRCADLTAVDPVR | N5H5F3S0T1 | 01Y41Y41M(31M)61M(21Y(31F)41L31Y(31F)41L32T)61Y31F |
| Q499Y3 | 122 | STQASSGENSTK | N4H4F1S0T1 | 01Y41Y41M(31M)61M(21Y41L32T)61Y31F |
| Q495M3 | 477 | SEDSHPFSNSTTFVR | N4H5F1S1T1 | 01Y(61F)41Y41M(31M)61M(21Y41L32T)61Y41L32S |
| Q495M3 | 477 | SEDSHPFSNSTTFVR | N4H5F1S1T1 | 01Y(61F)41Y41M(31M)61M(21Y41L32S)61Y41L32T |
| Q7Z6M1 | 108 | IWVFGGANQSGNR | N8H8F2S1T1 | 01Y41Y41M(31M(21Y41L31Y41L)41Y(31F)41L31Y)61M(21Y(31F)41L32T)61Y41L32S |
| P11279 | 84 | SSCGKENTSDPSLVIAFGR | N4H4F2S0T1 | 01Y(61F)41Y41M(31M)61M(21Y(31F)41L32T)61Y |
| Q9UGU0 | 1275 | NSSTEDKGR | N6H5F1S1T1 | 01Y41Y41M(31M41Y)(41Y)61M(21Y(31F)41L32T)61Y41L32S |
| Q06190 | 222 | IDNFSSGTDIK | N4H4F1S0T1 | 01Y41Y41M(31M)61M(21Y(31F)41L32T)61Y |
| Q86VY9 | 348 | NNSIGESLSSQYK | N5H5F3S0T1 | 01Y41Y41M(31M)61M(21Y(31F)41L31Y(31F)41L32T)61Y31F |
| P15328 | 161 | SNWHKGWNWTSGFNK | N4H4F2S0T1 | 01Y(61F)41Y41M(31M41Y)61M21Y(31F)41L32T |
| O14709 | 419 | NHSGEKPYKCNECGK | N4H4F0S0T1 | 01Y41Y41M(31M41Y)61M61Y41L32T |
| P14625 | 217 | HNNDTQHIWESDSNEFSVIADPR | N4H4F1S0T1 | 01Y(61F)41Y41M(31M41Y)61M61Y41L32T |
| Q8WXE9 | 344 | SQNSSISSTTGK | N4H4F1S0T1 | 01Y41Y41M(31M41Y)61M21Y(31F)41L32T |
| Q9Y2G7 | 99 | DIYEMNLSQWK | N5H5F3S0T1 | 01Y41Y41M(31M)61M(21Y(31F)41L31Y(31F)41L32T)61Y31F |
| P15328 | 167 | SNWHKGWNWTSGFNK | N4H4F2S0T1 | 01Y(61F)41Y41M(31M41Y)61M21Y(31F)41L32T |
| Q7Z6M1 | 108 | IWVFGGANQSGNR | N8H8F2S1T1 | 01Y(61F)41Y41M(31M(21Y41L31Y41L)41Y(31F)41L31Y)61M(21Y41L32S)61Y41L32T |
| Q7Z6M1 | 108 | IWVFGGANQSGNR | N8H8F2S1T1 | 01Y(61F)41Y41M(31M(21Y41L31Y41L)41Y(31F)41L31Y)61M(21Y41L32T)61Y41L32S |
| O14709 | 429 | NHSGEKPYKCNECGK | N4H4F0S0T1 | 01Y41Y41M(31M41Y)61M61Y41L32T |
| Q4KMQ2 | 493 | NINGTDPIQK | N3H5F1S0T1 | 01Y41Y41M(31M21Y(31F)41L32T)61M61M |
| P35613 | 160 | ILLTCSLNDSATEVTGHR | N5H5F2S1T1 | 01Y41Y41M(31M41Y31F)61M(21Y(31F)41L32T)61Y41L32S |
| Q92830 | 115 | ANETCKCNGWK | N5H5F1S1T1 | 01Y41Y41M(31M41Y41L32T)61M(21Y41L32S)61Y31F |
| Q5TD97 | 73 | CNHSLVEKPFAAKDER | N10H8F2S1T1 | 01Y(61F)41Y41M(31M(21Y41L31Y)41Y)(41Y)61M(21Y(31F)41L31Y41L32T)61Y41L31Y41L32S |
| Q5TD97 | 73 | CNHSLVEKPFAAKDER | N10H8F2S1T1 | 01Y(61F)41Y41M(31M(21Y41L31Y)41Y)(41Y)61M(21Y(31F)41L31Y41L32S)61Y41L31Y41L32T |
| Q7Z6M1 | 108 | IWVFGGANQSGNR | N8H8F2S1T1 | 01Y(61F)41Y41M(31M(21Y41L31Y41L)41Y41L31Y)61M(21Y(31F)41L32T)61Y41L32S |
| P11279 | 84 | SSCGKENTSDPSLVIAFGR | N6H6F2S1T1 | 01Y(61F)41Y41M(31M(21Y41L)41Y)61M(21Y(31F)41L32T)61Y41L32S |
| Q9P2D7 | 2263 | TSANQTQDFIDSK | N5H4F2S0T1 | 01Y41Y41M(31M(21Y31F)41Y31F)61M61Y41L32T |
| P11279 | 84 | SSCGKENTSDPSLVIAFGR | N6H6F2S1T1 | 01Y(61F)41Y41M(31M41Y41L31Y)61M(21Y(31F)41L32T)61Y41L32S |
| P15328 | 161 | SNWHKGWNWTSGFNK | N4H4F2S0T1 | 01Y41Y41M(31M)61M(21Y(31F)41L32T)61Y31F |
| Q9H3E2 | 23 | DYILSWYGNLSR | N5H5F3S0T1 | 01Y41Y41M(31M)61M(21Y(31F)41L31Y(31F)41L32T)61Y31F |
| P15328 | 167 | SNWHKGWNWTSGFNK | N4H4F2S0T1 | 01Y41Y41M(31M)61M(21Y(31F)41L32T)61Y31F |
| Q9UGU0 | 1275 | NSSTEDKGR | N6H5F1S1T1 | 01Y41Y41M(31M(21Y31F)41Y)61M(21Y41L32S)61Y41L32T |
| Q9UGU0 | 1275 | NSSTEDKGR | N6H5F1S1T1 | 01Y41Y41M(31M(21Y31F)41Y)61M(21Y41L32T)61Y41L32S |
| Q9P219 | 1319 | LDNHCELLSR | N4H4F1S0T1 | 01Y41Y41M(31M41Y)61M21Y(31F)41L32T |
| P11279 | 84 | SSCGKENTSDPSLVIAFGR | N4H4F2S0T1 | 01Y41Y41M(31M)61M(21Y(31F)41L32T)61Y31F |
| O15254 | 548 | SGSSDFEARNK | N4H4F1S0T1 | 01Y41Y41M(31M)61M(21Y(31F)41L32T)61Y |
| Q7Z3H0 | 211 | NRCADLTAVDPVR | N5H5F3S0T1 | 01Y41Y41M(31M(21Y(31F)41L21F)41Y31F)61M61Y41L32T |
| Q9NUB1 | 554 | MDDVINISGHR | N4H4F1S0T1 | 01Y41Y41M(31M41Y)61M21Y(31F)41L32T |
| Q9H3E2 | 23 | DYILSWYGNLSR | N5H5F3S0T1 | 01Y41Y41M(31M(21Y(31F)41L21F)41Y31F)61M61Y41L32T |
| P49792 | 1359 | EGSWWHCNSCSLK | N4H5F1S1T1 | 01Y41Y41M(31M)61M(21Y(31F)41L32T)61Y41L32S |
| Q8WUM0 | 1082 | DNWSSSDGK | N4H4F1S0T1 | 01Y41Y41M(31M)61M(21Y(31F)41L32T)61Y |
| Q5TD97 | 73 | CNHSLVEKPFAAKDER | N10H8F2S1T1 | 01Y(61F)41Y41M(31M(21Y(31F)41L31Y41L32S)41Y41L31Y41L32T)(41Y)61M61Y41L31Y41V |
| Q5TD97 | 73 | CNHSLVEKPFAAKDER | N10H8F2S1T1 | 01Y(61F)41Y41M(31M(21Y(31F)41L31Y41L32T)41Y41L31Y41L32S)(41Y)61M61Y41L31Y41V |
| Q8N9B8 | 371 | TALQGATQRSQMANSSR | N7H7F1S1T1 | 01Y41Y41M(31M(21Y41L31Y41L)41Y31F)61M(21Y41L32T)61Y41L32S |
| Q8N9B8 | 371 | TALQGATQRSQMANSSR | N7H7F1S1T1 | 01Y41Y41M(31M(21Y41L31Y41L)41Y31F)61M(21Y41L32S)61Y41L32T |
| Q86VY9 | 348 | NNSIGESLSSQYK | N5H5F3S0T1 | 01Y41Y41M(31M(21Y(31F)41L21F)41Y31F)61M61Y41L32T |
| Q14123 | 495 | TSGSEGSAPINNSVISVDYK | N4H4F2S0T1 | 01Y(61F)41Y41M(31M)61M(21Y(31F)41L32T)61Y |
| Q8N9V7 | 734 | ENVSMMMLGPQTLSIR | N7H7F1S1T1 | 01Y41Y41M(31M(21Y41L31Y41L)41Y31F)61M(21Y41L32T)61Y41L32S |
| Q8N9V7 | 734 | ENVSMMMLGPQTLSIR | N7H7F1S1T1 | 01Y41Y41M(31M(21Y41L31Y41L)41Y31F)61M(21Y41L32S)61Y41L32T |
| Q9Y2G7 | 99 | DIYEMNLSQWK | N5H5F3S0T1 | 01Y41Y41M(31M(21Y(31F)41L21F)41Y31F)61M61Y41L32T |
| Q6P444 | 257 | TNYSHHSKSQR | N6H6F0S1T1 | 01Y41Y41M(31M41Y41L)(41Y)61M(21Y41L32T)61Y41L32S |
| Q6P444 | 257 | TNYSHHSKSQR | N6H6F0S1T1 | 01Y41Y41M(31M41Y41L)(41Y)61M(21Y41L32S)61Y41L32T |
| Q8NDI1 | 104 | EWTFVIENESPSGRR | N7H7F1S1T1 | 01Y41Y41M(31M(21Y41L31Y41L)41Y31F)61M(21Y41L32T)61Y41L32S |
| Q8NDI1 | 104 | EWTFVIENESPSGRR | N7H7F1S1T1 | 01Y41Y41M(31M(21Y41L31Y41L)41Y31F)61M(21Y41L32S)61Y41L32T |
| Q6P444 | 257 | TNYSHHSKSQR | N6H6F2S1T1 | 01Y(61F)41Y41M(31M41Y41L)(41Y)61M(21Y(31F)41L32T)61Y41L32S |
| Q9NRP7 | 381 | SGTGEVPSAPRENR | N8H7F2S1T1 | 01Y41Y41M(31M(21Y(31F)41L31Y)41Y(31F)41L31Y)61M(21Y41L32T)61Y41L32S |
| Q9NRP7 | 381 | SGTGEVPSAPRENR | N8H7F2S1T1 | 01Y41Y41M(31M(21Y(31F)41L31Y)41Y(31F)41L31Y)61M(21Y41L32S)61Y41L32T |
| Q6P444 | 257 | TNYSHHSKSQR | N6H6F0S1T1 | 01Y41Y41M(31M41Y41L31Y)61M(21Y41L32T)61Y41L32S |
| Q6P444 | 257 | TNYSHHSKSQR | N6H6F0S1T1 | 01Y41Y41M(31M41Y41L31Y)61M(21Y41L32S)61Y41L32T |
| Q6P444 | 257 | TNYSHHSKSQR | N6H6F2S1T1 | 01Y(61F)41Y41M(31M41Y41L31Y)61M(21Y(31F)41L32T)61Y41L32S |
| B1AJZ9 | 1177 | DHQNESFLDLK | N6H6F0S1T1 | 01Y41Y41M(31M41Y41L)(41Y)61M(21Y41L32T)61Y41L32S |
| B1AJZ9 | 1177 | DHQNESFLDLK | N6H6F0S1T1 | 01Y41Y41M(31M41Y41L)(41Y)61M(21Y41L32S)61Y41L32T |
| P11279 | 103 | GHTLTLNFTR | N3H5F1S0T1 | 01Y(61F)41Y41M(31M41Y41L32T)61M61M |
| P08922 | 1738 | TGENSTSLPESFK | N8H7F2S1T1 | 01Y41Y41M(31M(21Y(31F)41L31Y)41Y(31F)41L31Y)61M(21Y41L32T)61Y41L32S |
| P08922 | 1738 | TGENSTSLPESFK | N8H7F2S1T1 | 01Y41Y41M(31M(21Y(31F)41L31Y)41Y(31F)41L31Y)61M(21Y41L32S)61Y41L32T |
| Q9NRP7 | 381 | SGTGEVPSAPRENR | N8H7F2S1T1 | 01Y41Y41M(31M(21Y41L31Y31F)41Y(31F)41L31Y)61M(21Y41L32T)61Y41L32S |
| Q9NRP7 | 381 | SGTGEVPSAPRENR | N8H7F2S1T1 | 01Y41Y41M(31M(21Y41L31Y31F)41Y(31F)41L31Y)61M(21Y41L32S)61Y41L32T |
| Q3C1V9 | 85 | CRHAGWNR | N4H4F1S0T1 | 01Y41Y41M(31M)61M(21Y(31F)41L32T)61Y |
| P48681 | 562 | TLENQSHETLER | N8H7F2S1T1 | 01Y41Y41M(31M(21Y(31F)41L31Y)41Y(31F)41L31Y)61M(21Y41L32T)61Y41L32S |
| P48681 | 562 | TLENQSHETLER | N8H7F2S1T1 | 01Y41Y41M(31M(21Y(31F)41L31Y)41Y(31F)41L31Y)61M(21Y41L32S)61Y41L32T |
| P07306 | 147 | SLSCQMAALQGNGSER | N5H5F3S1T1 | 01Y41Y41M(31M41Y31F)61M(21Y(31F)41L32S)61Y(31F)41L32T |
| P07306 | 147 | SLSCQMAALQGNGSER | N5H5F3S1T1 | 01Y41Y41M(31M41Y31F)61M(21Y(31F)41L32T)61Y(31F)41L32S |
| Q9H2X9 | 53 | ESSPFINSTDTEK | N5H4F2S0T1 | 01Y41Y41M(31M(21Y31F)41Y31F)61M61Y41L32T |
| P08922 | 1738 | TGENSTSLPESFK | N8H7F2S1T1 | 01Y41Y41M(31M(21Y41L31Y31F)41Y(31F)41L31Y)61M(21Y41L32T)61Y41L32S |
| P08922 | 1738 | TGENSTSLPESFK | N8H7F2S1T1 | 01Y41Y41M(31M(21Y41L31Y31F)41Y(31F)41L31Y)61M(21Y41L32S)61Y41L32T |
| B1AJZ9 | 1177 | DHQNESFLDLK | N6H6F0S1T1 | 01Y41Y41M(31M41Y41L31Y)61M(21Y41L32T)61Y41L32S |
| B1AJZ9 | 1177 | DHQNESFLDLK | N6H6F0S1T1 | 01Y41Y41M(31M41Y41L31Y)61M(21Y41L32S)61Y41L32T |
| Q0D2K5 | 95 | SGYQGNRCQK | N4H4F1S0T1 | 01Y41Y41M(31M)61M(21Y(31F)41L32T)61Y |
| P15328 | 161 | SNWHKGWNWTSGFNK | N5H5F2S1T1 | 01Y(61F)41Y41M(31M41Y)61M(21Y(31F)41L32T)61Y41L32S |
| P15328 | 167 | SNWHKGWNWTSGFNK | N5H5F2S1T1 | 01Y(61F)41Y41M(31M41Y)61M(21Y(31F)41L32T)61Y41L32S |
| P48681 | 562 | TLENQSHETLER | N8H7F2S1T1 | 01Y41Y41M(31M(21Y41L31Y31F)41Y(31F)41L31Y)61M(21Y41L32T)61Y41L32S |
| P48681 | 562 | TLENQSHETLER | N8H7F2S1T1 | 01Y41Y41M(31M(21Y41L31Y31F)41Y(31F)41L31Y)61M(21Y41L32S)61Y41L32T |
| O15254 | 548 | SGSSDFEARNK | N4H4F1S0T1 | 01Y41Y41M(31M41Y)61M21Y(31F)41L32T |
| P45973 | 93 | SNFSNSADDIK | N4H4F1S0T1 | 01Y41Y41M(31M)61M(21Y(31F)41L32T)61Y |
| P49792 | 1359 | EGSWWHCNSCSLK | N4H4F0S0T1 | 01Y41Y41M(31M41Y)61M61Y41L32T |
| Q8N9B8 | 371 | TALQGATQRSQMANSSR | N7H7F1S1T1 | 01Y(61F)41Y41M(31M41Y41L31Y41L)(41Y)61M(21Y41L32T)61Y41L32S |
| Q8N9B8 | 371 | TALQGATQRSQMANSSR | N7H7F1S1T1 | 01Y(61F)41Y41M(31M41Y41L31Y41L)(41Y)61M(21Y41L32S)61Y41L32T |
| Q7LG56 | 23 | SSSDTNESEIK | N4H4F1S0T1 | 01Y41Y41M(31M41Y)61M21Y(31F)41L32T |
| Q8N9V7 | 734 | ENVSMMMLGPQTLSIR | N7H7F1S1T1 | 01Y(61F)41Y41M(31M41Y41L31Y41L)(41Y)61M(21Y41L32T)61Y41L32S |
| Q8N9V7 | 734 | ENVSMMMLGPQTLSIR | N7H7F1S1T1 | 01Y(61F)41Y41M(31M41Y41L31Y41L)(41Y)61M(21Y41L32S)61Y41L32T |
| O14513 | 249 | TRVFDLEQQNR | N6H6F0S1T1 | 01Y41Y41M(31M41Y41L)(41Y)61M(21Y41L32T)61Y41L32S |
| O14513 | 249 | TRVFDLEQQNR | N6H6F0S1T1 | 01Y41Y41M(31M41Y41L)(41Y)61M(21Y41L32S)61Y41L32T |
| Q9HD45 | 174 | IVDVNLTSEGK | N4H4F3S0T1 | 01Y(61F)41Y41M(31M)61M(21Y(31F)41L32T)61Y31F |
| Q8NDI1 | 104 | EWTFVIENESPSGRR | N7H7F1S1T1 | 01Y(61F)41Y41M(31M41Y41L31Y41L)(41Y)61M(21Y41L32T)61Y41L32S |
| Q8NDI1 | 104 | EWTFVIENESPSGRR | N7H7F1S1T1 | 01Y(61F)41Y41M(31M41Y41L31Y41L)(41Y)61M(21Y41L32S)61Y41L32T |
| Q8NB90 | 333 | VNFTEIDKNSK | N4H7F2S1T1 | 01Y(61F)41Y41M(31M(21Y(31F)41L32S)41Y41L32T)61M(31M)61M |
| Q06190 | 222 | IDNFSSGTDIK | N4H4F1S0T1 | 01Y41Y41M(31M41Y)61M21Y(31F)41L32T |
| Q9HD45 | 174 | IVDVNLTSEGK | N4H4F3S0T1 | 01Y(61F)41Y41M(31M41Y31F)61M21Y(31F)41L32T |
| Q9BQI5 | 167 | NLSSEEVARPR | N4H4F1S0T1 | 01Y41Y41M(31M41Y)61M21Y(31F)41L32T |
| Q8WUM0 | 1082 | DNWSSSDGK | N4H4F1S0T1 | 01Y41Y41M(31M41Y)61M21Y(31F)41L32T |
| O14513 | 249 | TRVFDLEQQNR | N6H6F0S1T1 | 01Y41Y41M(31M41Y41L31Y)61M(21Y41L32T)61Y41L32S |
| O14513 | 249 | TRVFDLEQQNR | N6H6F0S1T1 | 01Y41Y41M(31M41Y41L31Y)61M(21Y41L32S)61Y41L32T |
| Q8WZ60 | 395 | ETQHDVWKYNSSINK | N4H4F2S0T1 | 01Y(61F)41Y41M(31M)61M(21Y(31F)41L32T)61Y |
| P15328 | 161 | SNWHKGWNWTSGFNK | N5H5F2S1T1 | 01Y(61F)41Y41M(31M)(41Y)61M(21Y(31F)41L32T)61Y41L32S |
| Q14123 | 495 | TSGSEGSAPINNSVISVDYK | N4H4F2S0T1 | 01Y41Y41M(31M)61M(21Y(31F)41L32T)61Y31F |
| P07306 | 147 | SLSCQMAALQGNGSER | N5H5F3S1T1 | 01Y(61F)41Y41M(31M41Y31F)61M(21Y(31F)41L32T)61Y41L32S |
| P15328 | 167 | SNWHKGWNWTSGFNK | N5H5F2S1T1 | 01Y(61F)41Y41M(31M)(41Y)61M(21Y(31F)41L32T)61Y41L32S |
| Q92613 | 484 | NLCYMISR | N4H4F1S0T1 | 01Y41Y41M(31M)61M(21Y(31F)41L32T)61Y |
| Q3C1V9 | 85 | CRHAGWNR | N4H4F1S0T1 | 01Y41Y41M(31M41Y)61M21Y(31F)41L32T |
| A8MYU2 | 666 | VSASTSSISNFTTR | N8H7F2S1T1 | 01Y41Y41M(31M(21Y(31F)41L31Y)41Y(31F)41L31Y)61M(21Y41L32T)61Y41L32S |
| A8MYU2 | 666 | VSASTSSISNFTTR | N8H7F2S1T1 | 01Y41Y41M(31M(21Y(31F)41L31Y)41Y(31F)41L31Y)61M(21Y41L32S)61Y41L32T |
| P49792 | 1359 | EGSWWHCNSCSLK | N4H5F1S1T1 | 01Y41Y41M(31M21Y(31F)41L32S)61M61Y41L32T |
| A8MYU2 | 666 | VSASTSSISNFTTR | N8H7F2S1T1 | 01Y41Y41M(31M(21Y41L31Y31F)41Y(31F)41L31Y)61M(21Y41L32T)61Y41L32S |
| A8MYU2 | 666 | VSASTSSISNFTTR | N8H7F2S1T1 | 01Y41Y41M(31M(21Y41L31Y31F)41Y(31F)41L31Y)61M(21Y41L32S)61Y41L32T |
| Q8NB90 | 333 | VNFTEIDKNSK | N4H7F2S1T1 | 01Y(61F)41Y41M(31M(21Y(31F)41L32T)41Y41L32S)61M(31M)61M |
| P45973 | 93 | SNFSNSADDIK | N4H4F1S0T1 | 01Y41Y41M(31M41Y)61M21Y(31F)41L32T |
| Q8WZ60 | 395 | ETQHDVWKYNSSINK | N4H4F2S0T1 | 01Y(61F)41Y41M(31M41Y)61M21Y(31F)41L32T |
| Q0D2K5 | 95 | SGYQGNRCQK | N4H4F1S0T1 | 01Y41Y41M(31M41Y)61M21Y(31F)41L32T |
| Q9H2X9 | 53 | ESSPFINSTDTEK | N5H4F2S0T1 | 01Y(61F)41Y41M(31M41Y31F)(41Y)61M61Y41L32T |
| Q9P2D7 | 2263 | TSANQTQDFIDSK | N5H4F2S0T1 | 01Y(61F)41Y41M(31M41Y31F)(41Y)61M61Y41L32T |
| P15328 | 167 | SNWHKGWNWTSGFNK | N4H4F3S0T1 | 01Y(61F)41Y41M(31M)61M(21Y(31F)41L32T)61Y31F |
| Q8N5R6 | 274 | STSEEKNNQSSK | N8H7F0S2T1 | 01Y41Y41M(31M(21Y41L31Y41L32T)41Y)(41Y)61M(21Y41L32S)61Y41L32S |
| Q9BQI5 | 167 | NLSSEEVARPR | N4H4F1S0T1 | 01Y41Y41M(31M)(41Y)61M21Y(31F)41L32T |
| Q9P219 | 1319 | LDNHCELLSR | N4H4F1S0T1 | 01Y(61F)41Y41M(31M41Y)61M61Y41L32T |
| P07098 | 271 | NFNTSR | N7H7F2S1T1 | 01Y(61F)41Y41M(31M(21Y(31F)41L31Y)41Y41L)61M(21Y41L32T)61Y41L32S |
| P07098 | 271 | NFNTSR | N7H7F2S1T1 | 01Y(61F)41Y41M(31M(21Y(31F)41L31Y)41Y41L)61M(21Y41L32S)61Y41L32T |
| O95789 | 513 | DFGERWGNYCK | N5H6F3S2T1 | 01Y(61F)41Y41M(31M(21Y(31F)41L32S)41Y(31F)41L32T)61M61Y41L32S |
| O95789 | 513 | DFGERWGNYCK | N5H6F3S2T1 | 01Y(61F)41Y41M(31M(21Y(31F)41L32T)41Y(31F)41L32S)61M61Y41L32S |
| Q7LG56 | 23 | SSSDTNESEIK | N4H4F1S0T1 | 01Y41Y41M(31M)61M(21Y(31F)41L32T)61Y |
| Q2LD37 | 4783 | NVDANNTENSTTVK | N6H5F3S1T1 | 01Y(61F)41Y41M(31M41Y)(41Y)61M(21Y(31F)41L32T)61Y(31F)41L32S |
| Q2LD37 | 4783 | NVDANNTENSTTVK | N6H5F3S1T1 | 01Y(61F)41Y41M(31M41Y)(41Y)61M(21Y(31F)41L32S)61Y(31F)41L32T |
| Q92613 | 484 | NLCYMISR | N4H4F1S0T1 | 01Y41Y41M(31M41Y)61M21Y(31F)41L32T |
| Q96L92 | 213 | REFANFTFPR | N6H6F4S1T1 | 01Y(61F)41Y41M(31M21Y(31F)41L21F)(41Y)61M(21Y(31F)41L32S)61Y41L32T |
| Q9NUB1 | 554 | MDDVINISGHR | N4H4F1S0T1 | 01Y(61F)41Y41M(31M41Y)61M61Y41L32T |
| P15328 | 161 | SNWHKGWNWTSGFNK | N4H4F3S0T1 | 01Y(61F)41Y41M(31M)61M(21Y(31F)41L32T)61Y31F |
| P14653 | 209 | TNFTTR | N7H7F2S1T1 | 01Y(61F)41Y41M(31M(21Y(31F)41L31Y)41Y41L)61M(21Y41L32T)61Y41L32S |
| P14653 | 209 | TNFTTR | N7H7F2S1T1 | 01Y(61F)41Y41M(31M(21Y(31F)41L31Y)41Y41L)61M(21Y41L32S)61Y41L32T |
| Q7LG56 | 23 | SSSDTNESEIK | N4H4F1S0T1 | 01Y(61F)41Y41M(31M41Y)61M61Y41L32T |
| Q8N5R6 | 274 | STSEEKNNQSSK | N8H7F0S2T1 | 01Y41Y41M(31M(21Y41L31Y41L32S)41Y)(41Y)61M(21Y41L32T)61Y41L32S |
| Q8N5R6 | 274 | STSEEKNNQSSK | N8H7F0S2T1 | 01Y41Y41M(31M(21Y41L31Y41L32S)41Y)(41Y)61M(21Y41L32S)61Y41L32T |
| Q9Y644 | 113 | VINTNCSAVRTR | N5H7F3S0T1 | 01Y41Y41M(31M(21Y(31F)41L31Y(31F)41L32T)41Y31F)61M(31M)61M |
| O95789 | 513 | DFGERWGNYCK | N5H6F3S2T1 | 01Y(61F)41Y41M(31M21Y(31F)41L32S)61M(21Y(31F)41L32T)61Y41L32S |
| O95789 | 513 | DFGERWGNYCK | N5H6F3S2T1 | 01Y(61F)41Y41M(31M21Y(31F)41L32T)61M(21Y(31F)41L32S)61Y41L32S |
| Q9UPS6 | 1765 | YLNSSR | N7H7F2S1T1 | 01Y(61F)41Y41M(31M(21Y(31F)41L31Y)41Y41L)61M(21Y41L32T)61Y41L32S |
| Q9UPS6 | 1765 | YLNSSR | N7H7F2S1T1 | 01Y(61F)41Y41M(31M(21Y(31F)41L31Y)41Y41L)61M(21Y41L32S)61Y41L32T |
| Q8IYW2 | 1164 | LGQNFSMEIQK | N6H7F0S1T1 | 01Y41Y41M(31M41Y41L31Y41L)61M(21Y41L32T)61Y41L32S |
| Q8IYW2 | 1164 | LGQNFSMEIQK | N6H7F0S1T1 | 01Y41Y41M(31M41Y41L31Y41L)61M(21Y41L32S)61Y41L32T |
| Q8WV37 | 486 | IHTGERPYKCNECGK | N5H5F2S1T1 | 01Y(61F)41Y41M(31M41Y)61M(21Y(31F)41L32T)61Y41L32S |
| O76064 | 463 | MVNNLSSEVK | N4H6F0S1T1 | 01Y41Y41M(31M(21Y41L32S)41Y41L32T)61M61M |
| O76064 | 463 | MVNNLSSEVK | N4H6F0S1T1 | 01Y41Y41M(31M(21Y41L32T)41Y41L32S)61M61M |
| P84996 | 114 | EAAVNFSYR | N4H4F1S0T1 | 01Y41Y41M(31M)61M(21Y(31F)41L32T)61Y |
| Q7LG56 | 23 | SSSDTNESEIK | N4H4F1S0T1 | 01Y41Y41M(31M)61M(21Y41L32T)61Y31F |
| Q9Y644 | 113 | VINTNCSAVRTR | N5H7F3S0T1 | 01Y(61F)41Y41M(31M(21Y(31F)41L31Y41L32T)41Y31F)61M(31M)61M |
| Q8TD57 | 151 | DRTGQGLPSSGNR | N6H9F3S0T1 | 01Y(61F)41Y41M(31M(21Y(31F)41L31Y41L32T)41Y(31F)41L31Y41L)61M(31M)61M |
| Q13637 | 220 | AENKSQCC | N4H4F1S0T1 | 01Y41Y41M(31M)61M(21Y(31F)41L32T)61Y |
| Q08345 | 777 | NCLVGENFTIK | N6H7F0S1T1 | 01Y41Y41M(31M41Y41L31Y41L)61M(21Y41L32T)61Y41L32S |
| Q08345 | 777 | NCLVGENFTIK | N6H7F0S1T1 | 01Y41Y41M(31M41Y41L31Y41L)61M(21Y41L32S)61Y41L32T |
| Q9NUB1 | 554 | MDDVINISGHR | N4H4F1S0T1 | 01Y41Y41M(31M)61M(21Y(31F)41L32T)61Y |
| Q8TDY4 | 759 | NSSRTLVQGCAR | N6H6F3S0T1 | 01Y41Y41M(31M(21Y41L21F)41Y41L21F)61M(21Y41L32T)61Y31F |
| Q13009 | 907 | AADALNSSMLK | N4H6F0S1T1 | 01Y41Y41M(31M(21Y41L32S)41Y41L32T)61M61M |
| Q13009 | 907 | AADALNSSMLK | N4H6F0S1T1 | 01Y41Y41M(31M(21Y41L32T)41Y41L32S)61M61M |
| P07098 | 271 | NFNTSR | N7H7F2S1T1 | 01Y(61F)41Y41M(31M(21Y41L31Y41L)41Y31F)61M(21Y41L32T)61Y41L32S |
| P07098 | 271 | NFNTSR | N7H7F2S1T1 | 01Y(61F)41Y41M(31M(21Y41L31Y41L)41Y31F)61M(21Y41L32S)61Y41L32T |
| Q8TD57 | 151 | DRTGQGLPSSGNR | N6H9F3S0T1 | 01Y(61F)41Y41M(31M(21Y(31F)41L31Y41L32T)41Y41L31Y(31F)41L)61M(31M)61M |
| P84996 | 114 | EAAVNFSYR | N4H4F1S0T1 | 01Y(61F)41Y41M(31M41Y)61M61Y41L32T |
| P84996 | 114 | EAAVNFSYR | N4H4F1S0T1 | 01Y41Y41M(31M41Y)61M21Y(31F)41L32T |
| Q9NUB1 | 554 | MDDVINISGHR | N4H4F1S0T1 | 01Y41Y41M(31M)61M(21Y41L32T)61Y31F |
| Q9UEF7 | 159 | VLPNGSAGVPNR | N4H6F0S1T1 | 01Y41Y41M(31M(21Y41L32S)41Y41L32T)61M61M |
| Q9UEF7 | 159 | VLPNGSAGVPNR | N4H6F0S1T1 | 01Y41Y41M(31M(21Y41L32T)41Y41L32S)61M61M |
| Q9Y644 | 113 | VINTNCSAVRTR | N5H7F3S0T1 | 01Y(61F)41Y41M(31M(21Y41L31Y(31F)41L32T)41Y31F)61M(31M)61M |
| O14599 | 41 | NLSDVNILHR | N4H6F0S1T1 | 01Y41Y41M(31M(21Y41L32S)41Y41L32T)61M61M |
| O14599 | 41 | NLSDVNILHR | N4H6F0S1T1 | 01Y41Y41M(31M(21Y41L32T)41Y41L32S)61M61M |
| Q13637 | 220 | AENKSQCC | N4H4F1S0T1 | 01Y41Y41M(31M41Y)61M21Y(31F)41L32T |
| Q9H7E2 | 323 | NDTRQPR | N7H4F3S0T1 | 01Y41Y41M(31M(21Y31F)41Y31F)(41Y)61M(21Y41L32T)61Y31F |
| Q5VV16 | 163 | HNLSLNDCFVK | N5H6F2S1T1 | 01Y(61F)41Y41M(31M(21Y(31F)41L32S)41Y41L)61M61Y41L32T |
| P23276 | 191 | WTSLNFNRTLR | N5H4F2S0T1 | 01Y(61F)41Y41M(31M41Y)61M(21Y41L32T)61Y31F |
| Q8N815 | 234 | ASIENSTPSQLQGEK | N7H7F6S1T1 | 01Y(61F)41Y41M(31M(21Y(31F)41L21F)41Y(31F)41L21F)(41Y)61M(21Y(31F)41L32S)61Y41L32T |
| Q6KC79 | 654 | VETQTEELKQNESR | N3H6F1S0T1 | 01Y(61F)41Y41M(31M41Y41L32T)61M(31M)61M |
| Q9Y6W3 | 366 | GELLCSYSNNK | N6H9F3S0T1 | 01Y(61F)41Y41M(31M(21Y(31F)41L31Y41L32T)41Y(31F)41L31Y41L)61M(31M)61M |

**Supplemental Table 3.** The complete KEGG pathways of N-glycoproteins corresponding to intact N-glycopeptides with Neu5Gc identified form MCF-7 cancer cells; PV=P-value, FE=Fold Enrichment.

| Term | Genes | PV | FE |
| --- | --- | --- | --- |
| Herpes simplex virus 1 infection | Q3KNS6, Q6ZMW2, A6NNF4, P05198, P51522, O95780 | 0.0 | 4 |
| Hypertrophic cardiomyopathy | O60359, P02545, Q8WZ42 | 0.0 | 10 |
| Dilated cardiomyopathy | O60359, P02545, Q8WZ42 | 0.0 | 9 |
| Arrhythmogenic right ventricular cardiomyopathy | O60359, P02545 | 0.2 | 8 |
| ErbB signaling pathway | P56975, O43639 | 0.2 | 7 |
| T cell receptor signaling pathway | P06239, O43639 | 0.3 | 6 |
| Amyotrophic lateral sclerosis | P56975, Q96DT5, P05198 | 0.3 | 2 |
| Lysosome | Q9Y227, Q9UK23 | 0.3 | 5 |
| Apoptosis | P02545, P05198 | 0.4 | 4 |
| Measles | O15350, P05198 | 0.4 | 4 |
| Spliceosome | Q6NWY9, P62304 | 0.4 | 4 |
| Hippo signaling pathway | O15350, P82279 | 0.4 | 4 |
| Pathways of neurodegeneration - multiple diseases | Q96DT5, P05198 | 0.8 | 1 |
| Pathogenic Escherichia coli infection | O43639 | 1.0 | 2 |
| Th1 and Th2 cell differentiation | P06239 | 1.0 | 3 |
| Hedgehog signaling pathway | Q9UMX1 | 1.0 | 5 |
| Cell adhesion molecules | P33681 | 1.0 | 2 |
| Calcium signaling pathway | P35367 | 1.0 | 1 |
| Neurotrophin signaling pathway | O15350 | 1.0 | 3 |
| Parkinson disease | P05198 | 1.0 | 1 |
| Adrenergic signaling in cardiomyocytes | O60359 | 1.0 | 2 |
| Non-alcoholic fatty liver disease | P05198 | 1.0 | 2 |
| RNA degradation | Q9H9A5 | 1.0 | 4 |
| Viral myocarditis | P33681 | 1.0 | 5 |
| NF-kappa B signaling pathway | P06239 | 1.0 | 3 |
| Inflammatory mediator regulation of TRP channels | P35367 | 1.0 | 3 |
| Osteoclast differentiation | P06239 | 1.0 | 2 |
| Pyrimidine metabolism | Q9Y227 | 1.0 | 5 |
| Oxytocin signaling pathway | O60359 | 1.0 | 2 |
| Nucleotide metabolism | Q9Y227 | 1.0 | 4 |
| Pathways in cancer | Q9UMX1 | 1.0 | 1 |
| Huntington disease | Q96DT5 | 1.0 | 1 |
| PD-L1 expression and PD-1 checkpoint pathway in cancer | P06239 | 1.0 | 3 |
| Autoimmune thyroid disease | P33681 | 1.0 | 6 |
| Toll-like receptor signaling pathway | P33681 | 1.0 | 3 |
| Basal cell carcinoma | Q9UMX1 | 1.0 | 5 |
| Prion disease | P05198 | 1.0 | 1 |
| Cardiac muscle contraction | O60359 | 1.0 | 3 |
| Human T-cell leukemia virus 1 infection | P06239 | 1.0 | 1 |
| Hepatitis C | P05198 | 1.0 | 2 |
| MAPK signaling pathway | O60359 | 1.0 | 1 |
| p53 signaling pathway | O15350 | 1.0 | 4 |
| Purine metabolism | Q9Y227 | 1.0 | 2 |
| Graft-versus-host disease | P33681 | 1.0 | 7 |
| Glycosaminoglycan biosynthesis - chondroitin sulfate / dermatan sulfate | Q9NS84 | 1.0 | 15 |
| TGF-beta signaling pathway | Q6NW40 | 1.0 | 3 |
| Autophagy - animal | P05198 | 1.0 | 2 |
| Allograft rejection | P33681 | 1.0 | 8 |
| Ras signaling pathway | Q7Z569 | 1.0 | 1 |
| Systemic lupus erythematosus | P33681 | 1.0 | 2 |
| Lipid and atherosclerosis | P05198 | 1.0 | 1 |
| Influenza A | P05198 | 1.0 | 2 |
| Primary immunodeficiency | P06239 | 1.0 | 8 |
| Metabolic pathways | Q9Y227 | 1.0 | 0 |
| Intestinal immune network for IgA production | P33681 | 1.0 | 6 |
| Yersinia infection | P06239 | 1.0 | 2 |
| Neuroactive ligand-receptor interaction | P35367 | 1.0 | 1 |
| Protein processing in endoplasmic reticulum | P05198 | 1.0 | 2 |
| Cytokine-cytokine receptor interaction | Q14005 | 1.0 | 1 |
| Type I diabetes mellitus | P33681 | 1.0 | 7 |
| Alzheimer disease | P05198 | 1.0 | 1 |
| Rheumatoid arthritis | P33681 | 1.0 | 3 |
| Th17 cell differentiation | P06239 | 1.0 | 3 |
| Natural killer cell mediated cytotoxicity | P06239 | 1.0 | 2 |
| Axon guidance | O43639 | 1.0 | 2 |

**Supplemental Table 4.** The complete KEGG pathways of N-glycoproteins corresponding to intact N-glycopeptides with Neu5Gc identified form MCF-7 cancer stem cells; PV=P-value, FE=Fold Enrichment.

| Term | Genes | PV | FE |
| --- | --- | --- | --- |
| Metabolic pathways | Q7LG56, O15254, Q9UPS6, P07098, Q9UEF7, Q14123, Q9NUB1 | 0.7 | 1 |
| Amyotrophic lateral sclerosis | P49792, Q9P2D7, Q8TD57, Q8WUM0 | 0.2 | 2 |
| Thyroid hormone synthesis | P07306, P14625, P84996 | 0.0 | 9 |
| Hematopoietic cell lineage | P08174, P08571, P16070 | 0.1 | 7 |
| cAMP signaling pathway | Q13009, O15254, P84996 | 0.2 | 3 |
| Human papillomavirus infection | Q9Y644, Q06190, P84996 | 0.4 | 2 |
| Antifolate resistance | P33527, P15328 | 0.1 | 15 |
| Propanoate metabolism | O15254, Q9NUB1 | 0.1 | 14 |
| Endocrine and other factor-regulated calcium reabsorption | Q9UEF7, P84996 | 0.2 | 9 |
| Notch signaling pathway | Q9Y644, Q92830 | 0.2 | 8 |
| Viral life cycle - HIV-1 | P49792, Q92830 | 0.2 | 7 |
| Renin secretion | P84996, Q14123 | 0.3 | 7 |
| Insulin secretion | A8MYU2, P84996 | 0.3 | 5 |
| Morphine addiction | P84996, Q14123 | 0.3 | 5 |
| Amoebiasis | P08571, P84996 | 0.4 | 4 |
| Parathyroid hormone synthesis, secretion and action | Q9UEF7, P84996 | 0.4 | 4 |
| Nucleocytoplasmic transport | P49792, Q8WUM0 | 0.4 | 4 |
| Carbon metabolism | O15254, Q9NUB1 | 0.4 | 4 |
| Sphingolipid signaling pathway | P33527, Q06190 | 0.4 | 4 |
| Purine metabolism | Q7LG56, Q14123 | 0.4 | 4 |
| Lysosome | P11279, Q99538 | 0.4 | 3 |
| Dopaminergic synapse | Q06190, P84996 | 0.4 | 3 |
| Vascular smooth muscle contraction | A8MYU2, P84996 | 0.4 | 3 |
| Estrogen signaling pathway | P14625, P84996 | 0.5 | 3 |
| Alcoholic liver disease | O15254, P08571 | 0.5 | 3 |
| Adrenergic signaling in cardiomyocytes | Q06190, P84996 | 0.5 | 3 |
| Phagosome | P08571, P11279 | 0.5 | 3 |
| Tight junction | Q13009, Q9Y3L5 | 0.5 | 3 |
| Tuberculosis | P08571, P11279 | 0.5 | 3 |
| Chemokine signaling pathway | Q13009, P51679 | 0.6 | 2 |
| Viral carcinogenesis | Q92830, P51679 | 0.6 | 2 |
| Proteoglycans in cancer | Q13009, P16070 | 0.6 | 2 |
| Rap1 signaling pathway | Q13009, P84996 | 0.6 | 2 |
| Chemical carcinogenesis - receptor activation | P14625, P84996 | 0.6 | 2 |
| Lipid and atherosclerosis | P08571, P14625 | 0.6 | 2 |
| Calcium signaling pathway | P84996, Q14123 | 0.6 | 2 |
| Shigellosis | P08571, P16070 | 0.7 | 2 |
| Salmonella infection | P08571, P14625 | 0.7 | 2 |
| Endocytosis | Q8TDY4, P15328 | 0.7 | 2 |
| Huntington disease | Q9P2D7, Q8TD57 | 0.7 | 1 |
| MicroRNAs in cancer | P33527, P16070 | 0.7 | 1 |
| PI3K-Akt signaling pathway | Q06190, P14625 | 0.8 | 1 |
| Pathways of neurodegeneration - multiple diseases | Q9P2D7, Q8TD57 | 0.9 | 1 |
| Herpes simplex virus 1 infection | Q9Y2G7, Q8WV37 | 0.9 | 1 |
| Pathways in cancer | P14625, P84996 | 0.9 | 1 |
| Vitamin digestion and absorption | P33527 | 1.0 | 9 |
| Inflammatory mediator regulation of TRP channels | P84996 | 1.0 | 2 |
| Fatty acid metabolism | O15254 | 1.0 | 4 |
| Melanogenesis | P84996 | 1.0 | 2 |
| Salivary secretion | P84996 | 1.0 | 2 |
| Endocrine resistance | P84996 | 1.0 | 2 |
| Cushing syndrome | P84996 | 1.0 | 1 |
| Viral protein interaction with cytokine and cytokine receptor | P51679 | 1.0 | 2 |
| Thyroid hormone signaling pathway | Q92830 | 1.0 | 2 |
| mRNA surveillance pathway | Q06190 | 1.0 | 2 |
| NF-kappa B signaling pathway | P08571 | 1.0 | 2 |
| Nucleotide metabolism | Q7LG56 | 1.0 | 3 |
| Dilated cardiomyopathy | P84996 | 1.0 | 2 |
| beta-Alanine metabolism | O15254 | 1.0 | 7 |
| Fc gamma R-mediated phagocytosis | Q8TDY4 | 1.0 | 2 |
| Platelet activation | P84996 | 1.0 | 2 |
| Pyrimidine metabolism | Q7LG56 | 1.0 | 4 |
| Aldosterone synthesis and secretion | P84996 | 1.0 | 2 |
| Signaling pathways regulating pluripotency of stem cells | P14653 | 1.0 | 2 |
| Other types of O-glycan biosynthesis | Q9Y644 | 1.0 | 5 |
| Gastric acid secretion | P84996 | 1.0 | 3 |
| Cytokine-cytokine receptor interaction | P51679 | 1.0 | 1 |
| AMPK signaling pathway | Q06190 | 1.0 | 2 |
| Pyruvate metabolism | Q9NUB1 | 1.0 | 5 |
| Biosynthesis of unsaturated fatty acids | O15254 | 1.0 | 8 |
| p53 signaling pathway | Q7LG56 | 1.0 | 3 |
| Pertussis | P08571 | 1.0 | 3 |
| Regulation of lipolysis in adipocytes | P84996 | 1.0 | 4 |
| Phospholipase D signaling pathway | P84996 | 1.0 | 2 |
| MAPK signaling pathway | P08571 | 1.0 | 1 |
| Antigen processing and presentation | Q99538 | 1.0 | 3 |
| Cortisol synthesis and secretion | P84996 | 1.0 | 3 |
| Relaxin signaling pathway | P84996 | 1.0 | 2 |
| PPAR signaling pathway | O15254 | 1.0 | 3 |
| Glycerolipid metabolism | P07098 | 1.0 | 4 |
| Viral myocarditis | P08174 | 1.0 | 4 |
| Glucagon signaling pathway | P84996 | 1.0 | 2 |
| Glutamatergic synapse | P84996 | 1.0 | 2 |
| Gap junction | P84996 | 1.0 | 3 |
| Prostate cancer | P14625 | 1.0 | 2 |
| Fluid shear stress and atherosclerosis | P14625 | 1.0 | 2 |
| Pentose and glucuronate interconversions | Q9UEF7 | 1.0 | 7 |
| Toll-like receptor signaling pathway | P08571 | 1.0 | 2 |
| Ras signaling pathway | Q13009 | 1.0 | 1 |
| Longevity regulating pathway | Q9UEF7 | 1.0 | 3 |
| Complement and coagulation cascades | P08174 | 1.0 | 3 |
| Cocaine addiction | P84996 | 1.0 | 5 |
| Transcriptional misregulation in cancer | P08571 | 1.0 | 1 |
| GnRH signaling pathway | P84996 | 1.0 | 2 |
| Peroxisome | O15254 | 1.0 | 3 |
| GABAergic synapse | Q9H2X9 | 1.0 | 3 |
| Ovarian steroidogenesis | P84996 | 1.0 | 4 |
| ECM-receptor interaction | P16070 | 1.0 | 3 |
| Circadian entrainment | P84996 | 1.0 | 2 |
| Alcoholism | P84996 | 1.0 | 1 |
| Ribosome biogenesis in eukaryotes | Q8NB90 | 1.0 | 2 |
| Growth hormone synthesis, secretion and action | P84996 | 1.0 | 2 |
| Ascorbate and aldarate metabolism | Q9UEF7 | 1.0 | 8 |
| Amphetamine addiction | P84996 | 1.0 | 3 |
| Fatty acid degradation | O15254 | 1.0 | 5 |
| Glycolysis / Gluconeogenesis | Q9NUB1 | 1.0 | 3 |
| Regulation of actin cytoskeleton | Q13009 | 1.0 | 1 |
| alpha-Linolenic acid metabolism | O15254 | 1.0 | 9 |
| Human cytomegalovirus infection | P84996 | 1.0 | 1 |
| Protein digestion and absorption | Q495M3 | 1.0 | 2 |
| Serotonergic synapse | P84996 | 1.0 | 2 |
| Fat digestion and absorption | P07098 | 1.0 | 5 |
| Vibrio cholerae infection | P84996 | 1.0 | 5 |
| Parkinson disease | P84996 | 1.0 | 1 |
| Autophagy - animal | P11279 | 1.0 | 2 |
| ABC transporters | P33527 | 1.0 | 5 |
| Olfactory transduction | Q14123 | 1.0 | 1 |
| Long-term depression | P84996 | 1.0 | 4 |
| Vasopressin-regulated water reabsorption | P84996 | 1.0 | 5 |
| Human T-cell leukemia virus 1 infection | Q92830 | 1.0 | 1 |
| Kaposi sarcoma-associated herpesvirus infection | P51679 | 1.0 | 1 |
| Oxytocin signaling pathway | P84996 | 1.0 | 1 |
| Chagas disease | P84996 | 1.0 | 2 |
| Glutathione metabolism | Q7LG56 | 1.0 | 4 |
| Glyoxylate and dicarboxylate metabolism | Q9NUB1 | 1.0 | 8 |
| Fanconi anemia pathway | O15360 | 1.0 | 4 |
| Taste transduction | Q14123 | 1.0 | 3 |
| Acute myeloid leukemia | P08571 | 1.0 | 3 |
| Bile secretion | P84996 | 1.0 | 3 |
| Pancreatic secretion | P84996 | 1.0 | 2 |
| Epstein-Barr virus infection | P16070 | 1.0 | 1 |
| Legionellosis | P08571 | 1.0 | 4 |
| cGMP-PKG signaling pathway | A8MYU2 | 1.0 | 1 |
| Protein processing in endoplasmic reticulum | P14625 | 1.0 | 1 |
| Lysine degradation | Q9UPS6 | 1.0 | 4 |
| Thermogenesis | P84996 | 1.0 | 1 |
| Drug metabolism - other enzymes | Q7LG56 | 1.0 | 3 |
| IL-17 signaling pathway | P14625 | 1.0 | 2 |

**Supplemental Table 5.** The complete INTERPRO domain list of N-glycoproteins corresponding to intact N-glycopeptides with Neu5Gc identified form MCF-7 cancer cells; PV=P-value, FE=Fold Enrichment.

| Term | Genes | PV | FE |
| --- | --- | --- | --- |
| Zinc finger C2H2-type/integrase DNA-binding domain | P28698, Q3KNS6, Q03924, O75626, Q6ZMW2, A6NNF4, Q86V15, P51522, O95780 | 0.0 | 4 |
| P-loop containing nucleoside triphosphate hydrolase | Q3L8U1, Q96DT5, Q5T7B8, A2PYH4, Q8IVL0, Q9NS84 | 0.2 | 2 |
| Krueppel-associated box | Q3KNS6, Q6ZMW2, A6NNF4, P51522, O95780 | 0.1 | 3 |
| Epidermal growth factor-like domain | P56975, Q9NZR2, P82279, Q9UK23 | 0.0 | 5 |
| Protein kinase, catalytic domain | P06239, Q96PY6, Q8WZ42 | 0.5 | 2 |
| Protein kinase-like domain | P06239, Q96PY6, Q8WZ42 | 0.5 | 2 |
| Sterile alpha motif domain | O15350, Q5T7B8 | 0.3 | 6 |
| Tyrosine-protein kinase, active site | P06239, Q8WZ42 | 0.3 | 6 |
| EGF-type aspartate/asparagine hydroxylation site | Q9NZR2, P82279 | 0.3 | 6 |
| EGF-like calcium-binding, conserved site | Q9NZR2, P82279 | 0.3 | 6 |
| SH2 domain | P06239, O43639 | 0.3 | 5 |
| Helicase, C-terminal | Q3L8U1, A2PYH4 | 0.3 | 5 |
| Helicase, superfamily 1/2, ATP-binding domain | Q3L8U1, A2PYH4 | 0.3 | 5 |
| Sterile alpha motif/pointed domain | O15350, Q5T7B8 | 0.3 | 5 |
| EGF-like calcium-binding | Q9NZR2, P82279 | 0.3 | 5 |
| Insulin-like growth factor binding protein, N-terminal | Q9NZR2, P82279 | 0.4 | 4 |
| AAA+ ATPase domain | Q96DT5, Q8IVL0 | 0.4 | 4 |
| PDZ domain | Q14005, A8MT19 | 0.4 | 4 |
| Src homology-3 domain | P06239, O43639 | 0.5 | 3 |
| Ankyrin repeat-containing domain | P0C6C1, O75179 | 0.5 | 3 |
| Ankyrin repeat | P0C6C1, O75179 | 0.6 | 2 |
| Zinc finger, RING-type | Q8WVD3, Q7Z569 | 0.6 | 2 |
| Protein kinase, ATP binding site | P06239, Q96PY6 | 0.7 | 2 |
| Immunoglobulin V-set | P33681, Q8WZ42 | 0.8 | 1 |
| Zinc finger, RING/FYVE/PHD-type | Q8WVD3, Q7Z569 | 0.8 | 1 |
| Immunoglobulin subtype | P33681, Q8WZ42 | 0.8 | 1 |
| G protein-coupled receptor, rhodopsin-like | O14718, P35367 | 0.9 | 1 |
| GPCR, rhodopsin-like, 7TM | O14718, P35367 | 0.9 | 1 |
| Immunoglobulin-like domain | P33681, Q8WZ42 | 0.9 | 1 |
| Immunoglobulin-like fold | P33681, Q8WZ42 | 1.0 | 1 |
| Dynein heavy chain, coiled coil stalk | Q96DT5 | 1.0 | 19 |
| p53 tumour suppressor family | O15350 | 1.0 | 101 |
| FERM, C-terminal PH-like domain | Q9HCS5 | 1.0 | 11 |
| SAP domain | Q8IV48 | 1.0 | 12 |
| Interleukin-16 | Q14005 | 1.0 | 302 |
| Repulsive guidance molecule, C-terminal | Q6NW40 | 1.0 | 101 |
| Myosin-binding domain | Q9HBM0 | 1.0 | 302 |
| Small nuclear ribonucleoprotein E | P62304 | 1.0 | 302 |
| Translation initiation factor 2, alpha subunit, middle domain | P05198 | 1.0 | 302 |
| PPAK motif | Q8WZ42 | 1.0 | 302 |
| Vezatin | Q9HBM0 | 1.0 | 302 |
| Serine/threonine-protein kinase, active site | Q96PY6 | 1.0 | 1 |
| Low-density lipoprotein (LDL) receptor class A repeat | Q9NZR2 | 1.0 | 6 |
| Ezrin/radixin/moesin like | Q9HCS5 | 1.0 | 17 |
| WW domain | Q6NWY9 | 1.0 | 6 |
| Lamin Tail Domain | P02545 | 1.0 | 60 |
| EGF-like, conserved site | P82279 | 1.0 | 9 |
| PR-domain zinc finger protein PRDM1 | O75626 | 1.0 | 302 |
| FF domain | Q6NWY9 | 1.0 | 50 |
| Sec63 domain | A2PYH4 | 1.0 | 76 |
| Fatty acid desaturase, type 1 | Q9Y5Q0 | 1.0 | 38 |
| Ribonuclease H-like domain | Q8IV48 | 1.0 | 4 |
| SH3-binding 5 | O60239 | 1.0 | 151 |
| Tetratricopeptide-like helical | Q9H9A5 | 1.0 | 1 |
| Suppressor of fused protein | Q9UMX1 | 1.0 | 302 |
| Cytochrome b5-like heme/steroid binding domain | Q9Y5Q0 | 1.0 | 20 |
| FAM178 family | Q8IX21 | 1.0 | 151 |
| CD80-like, immunoglobulin C2-set | P33681 | 1.0 | 10 |
| p53, DNA-binding domain | O15350 | 1.0 | 101 |
| Sequence-specific single-strand DNA-binding protein | P81877 | 1.0 | 101 |
| Kinesin, motor domain | Q5T7B8 | 1.0 | 7 |
| FERM adjacent (FA) | Q9HCS5 | 1.0 | 23 |
| p53, tetramerisation domain | O15350 | 1.0 | 101 |
| Cytoplasmic, NCK | O43639 | 1.0 | 151 |
| Chromo domain-like | Q3L8U1 | 1.0 | 9 |
| Voltage-dependent calcium channel, gamma subunit | O60359 | 1.0 | 38 |
| Transcription factor SOX | P41225 | 1.0 | 60 |
| WD40 repeat | Q96JK2 | 1.0 | 1 |
| Laminin G domain | P82279 | 1.0 | 5 |
| LDLR class B repeat | Q9NZR2 | 1.0 | 20 |
| BRO1 domain | A8MT19 | 1.0 | 50 |
| Transcription regulator SCAN | P28698 | 1.0 | 5 |
| Nucleic acid-binding, OB-fold | P05198 | 1.0 | 4 |
| Condensin-2 complex subunit D3 | P42695 | 1.0 | 302 |
| LisH dimerisation motif | P81877 | 1.0 | 11 |
| RNA recognition motif domain | Q8IUH3 | 1.0 | 1 |
| Dynein heavy chain domain | Q96DT5 | 1.0 | 20 |
| Sulfotransferase domain | Q9NS84 | 1.0 | 9 |
| FERM central domain | Q9HCS5 | 1.0 | 6 |
| Kinesin, motor region, conserved site | Q5T7B8 | 1.0 | 7 |
| RNA-binding domain, S1 | P05198 | 1.0 | 34 |
| Serine-threonine/tyrosine-protein kinase catalytic domain | P06239 | 1.0 | 2 |
| Zinc finger, UBP-type | Q7Z569 | 1.0 | 22 |
| Transmembrane protein 132 | Q14DG7 | 1.0 | 60 |
| Saposin B | P28039 | 1.0 | 43 |
| Tetratricopeptide repeat | Q9H9A5 | 1.0 | 2 |
| Fatty acid/sphingolipid desaturase | Q9Y5Q0 | 1.0 | 76 |
| Saposin-like | P28039 | 1.0 | 50 |
| SEFIR | Q8NFM7 | 1.0 | 50 |
| Dynein heavy chain, domain-2 | Q96DT5 | 1.0 | 19 |
| FERM domain | Q9HCS5 | 1.0 | 6 |
| Spc97/Spc98 | Q96RT7 | 1.0 | 60 |
| SNF2-related | Q3L8U1 | 1.0 | 9 |
| Tyrosine-protein kinase, catalytic domain | P06239 | 1.0 | 3 |
| Transthyretin, conserved site | Q8WZ42 | 1.0 | 151 |
| FERM/acyl-CoA-binding protein, 3-helical bundle | Q9HCS5 | 1.0 | 6 |
| Domain of unknown function DUF2233 | Q9UK23 | 1.0 | 302 |
| High mobility group (HMG) box domain | P41225 | 1.0 | 5 |
| Repulsive guidance molecule, N-terminal | Q6NW40 | 1.0 | 101 |
| Armadillo-type fold | P42695 | 1.0 | 1 |
| K Homology domain, type 1 | O75179 | 1.0 | 8 |
| WD40/YVTN repeat-like-containing domain | Q96JK2 | 1.0 | 1 |
| Translation initiation factor 2, alpha subunit, C-terminal | P05198 | 1.0 | 302 |
| Immunoglobulin subtype 2 | Q8WZ42 | 1.0 | 1 |
| BRK domain | Q3L8U1 | 1.0 | 50 |
| FERM conserved site | Q9HCS5 | 1.0 | 13 |
| Chromo domain/shadow | Q3L8U1 | 1.0 | 9 |
| Concanavalin A-like lectin/glucanase, subgroup | P82279 | 1.0 | 1 |
| Like-Sm (LSM) domain | P62304 | 1.0 | 13 |
| Low-density lipoprotein (LDL) receptor class A, conserved site | Q9NZR2 | 1.0 | 7 |
| Condensin subunit 1/Condensin-2 complex subunit D3 | P42695 | 1.0 | 151 |
| p53/RUNT-type transcription factor, DNA-binding domain | O15350 | 1.0 | 43 |
| Nucleoside phosphatase GDA1/CD39 | Q9Y227 | 1.0 | 34 |
| Calponin homology domain | Q8IVL0 | 1.0 | 4 |
| Complement Clr-like EGF domain | Q9NZR2 | 1.0 | 11 |
| Translation initiation factor 2, alpha subunit | P05198 | 1.0 | 302 |
| Pleckstrin homology-like domain | Q9HCS5 | 1.0 | 1 |
| Titin Z | Q8WZ42 | 1.0 | 302 |
| SET domain | O75626 | 1.0 | 6 |
| BRCA1-associated 2 | Q7Z569 | 1.0 | 302 |
| Carbohydrate sulfotransferase | Q9NS84 | 1.0 | 43 |
| Drought induced 19/ RING finger protein 114 | Q8WVD3 | 1.0 | 38 |
| Pericentriolar material 1 protein | Q15154 | 1.0 | 302 |
| Dynein heavy chain, P-loop containing D4 domain | Q96DT5 | 1.0 | 19 |
| Suppressor of fused-like | Q9UMX1 | 1.0 | 302 |
| Ribosomal protein S1, RNA-binding domain | P05198 | 1.0 | 34 |
| Band 4.1 domain | Q9HCS5 | 1.0 | 6 |
| Intermediate filament protein, conserved site | P02545 | 1.0 | 5 |
| Peptidase M28 | Q58DX5 | 1.0 | 25 |
| Six-bladed beta-propeller, TolB-like | Q9NZR2 | 1.0 | 7 |
| Nucleotide-binding, alpha-beta plait | Q8IUH3 | 1.0 | 1 |
| ATPase, AAA-type, core | Q8IVL0 | 1.0 | 6 |
| Homeodomain-like | Q9UKL3 | 1.0 | 1 |
| Ribonucleoprotein LSM domain | P62304 | 1.0 | 14 |
| Histamine H1 receptor | P35367 | 1.0 | 302 |
| Visual pigments (opsins) retinal binding site | O14718 | 1.0 | 27 |
| Lipase, GDSL | P28039 | 1.0 | 151 |
| Peropsin | O14718 | 1.0 | 151 |
| p53-like transcription factor, DNA-binding | O15350 | 1.0 | 7 |
| Armadillo-like helical | P42695 | 1.0 | 1 |
| Dynein heavy chain | Q96DT5 | 1.0 | 30 |
| Immunoglobulin I-set | Q8WZ42 | 1.0 | 2 |
| Chromo domain | Q3L8U1 | 1.0 | 11 |
| Suppressor of fused C-terminal | Q9UMX1 | 1.0 | 302 |
| Dynein heavy chain, domain-1 | Q96DT5 | 1.0 | 34 |
| Saposin-like type B, 2 | P28039 | 1.0 | 60 |
| K Homology domain | O75179 | 1.0 | 8 |
| PMP-22/EMP/MP20/Claudin | O60359 | 1.0 | 7 |
| Fibronectin, type III | Q8WZ42 | 1.0 | 1 |
| Synaptonemal complex 1 | Q15431 | 1.0 | 302 |
| Proline-rich Peptides | Q6ZRG5 | 1.0 | 43 |
| DNA/RNA helicase, DEAD/DEAH box type, N-terminal | A2PYH4 | 1.0 | 4 |
| FERM, N-terminal | Q9HCS5 | 1.0 | 11 |
| Exonuclease, RNase T/DNA polymerase III | Q8IV48 | 1.0 | 22 |
| Suppressor of fused domain | Q9UMX1 | 1.0 | 302 |

**Supplemental Table 6.** The complete INTERPRO domain list of N-glycoproteins corresponding to intact N-glycopeptides with Neu5Gc identified form MCF-7 cancer stem cells; PV=P-value, FE=Fold Enrichment.

| Term | Genes | PV | FE |
| --- | --- | --- | --- |
| Transcription regulator SCAN | O14709, Q16670 | 0.0 | 11 |
| Tyrosine-protein kinase, receptor class II, conserved site | P08922, Q08345 | 0.0 | 43 |
| Dynein heavy chain domain | Q9P2D7, Q8TD57 | 0.1 | 31 |
| Dynein heavy chain, coiled coil stalk | Q9P2D7, Q8TD57 | 0.1 | 29 |
| Dynein heavy chain, P-loop containing D4 domain | Q9P2D7, Q8TD57 | 0.1 | 29 |
| Dynein heavy chain, domain-2 | Q9P2D7, Q8TD57 | 0.1 | 29 |
| AAA+ ATPase domain | Q8NB90, P33527, Q8TD57 | 0.1 | 5 |
| Forkhead-associated (FHA) domain | B1AJZ9, O76064 | 0.1 | 13 |
| P-loop containing nucleoside triphosphate hydrolase | Q8NB90, P33527, Q13637, P84996, Q9P2D7, Q8TD57, Q9Y3L5 | 0.2 | 2 |
| Phox homologous domain | Q96L92, Q9H3E2 | 0.2 | 10 |
| SMAD/FHA domain | B1AJZ9, O76064 | 0.2 | 9 |
| Kelch repeat type 1 | Q8WZ60, Q7Z6M1 | 0.2 | 7 |
| Kelch-type beta propeller | Q8WZ60, Q7Z6M1 | 0.3 | 6 |
| Krueppel-associated box | Q9Y2G7, O14709, Q8WV37 | 0.3 | 2 |
| Calponin homology domain | Q9P219, Q8NDI1 | 0.3 | 6 |
| Tyrosine-protein kinase, catalytic domain | P08922, Q08345 | 0.3 | 5 |
| Zinc finger, PHD-type | Q9UGU0, Q92613 | 0.3 | 5 |
| Tyrosine-protein kinase, active site | P08922, Q08345 | 0.3 | 5 |
| C-type lectin-like | P07306, P16070 | 0.4 | 4 |
| Ion transport domain | A8MYU2, Q9NS40 | 0.4 | 4 |
| C-type lectin fold | P07306, P16070 | 0.4 | 4 |
| Protein kinase-like domain | P08922, Q9NRP7, Q08345, Q96L96 | 0.4 | 2 |
| Armadillo-type fold | Q9NRP7, Q6NXR4, Q6KC79 | 0.4 | 2 |
| Zinc finger C2H2-type/integrase DNA-binding domain | Q9Y2G7, O14709, Q8WV37, Q16670 | 0.4 | 1 |
| Small GTPase superfamily | Q13637, Q9Y3L5 | 0.5 | 3 |
| Serine-threonine/tyrosine-protein kinase catalytic domain | P08922, Q08345 | 0.5 | 3 |
| PDZ domain | Q96L92, Q13009 | 0.5 | 3 |
| Small GTP-binding protein domain | Q13637, Q9Y3L5 | 0.5 | 3 |
| Pleckstrin homology-like domain | P49792, Q13009, Q8TDY4 | 0.6 | 2 |
| Zinc finger, RING/FYVE/PHD-type | Q9UGU0, Q92613, O76064 | 0.6 | 1 |
| Armadillo-like helical | Q9NRP7, Q6KC79 | 0.6 | 2 |
| Protein kinase, catalytic domain | P08922, Q9NRP7, Q08345 | 0.6 | 1 |
| Ankyrin repeat-containing domain | Q8TDY4, Q7Z3H0 | 0.6 | 2 |
| Immunoglobulin subtype 2 | P35613, Q96L96 | 0.7 | 2 |
| Ankyrin repeat | Q8TDY4, Q7Z3H0 | 0.7 | 2 |
| Homeodomain | Q8N7G0, P14653 | 0.7 | 2 |
| Pleckstrin homology domain | Q13009, Q8TDY4 | 0.7 | 2 |
| Homeodomain-like | Q8N7G0, P14653 | 0.7 | 1 |
| Protein kinase, ATP binding site | P08922, Q9NRP7 | 0.8 | 1 |
| Immunoglobulin subtype | P35613, Q96L96 | 0.9 | 1 |
| Immunoglobulin-like fold | P08922, P35613, Q96L96 | 0.9 | 1 |
| Immunoglobulin-like domain | P35613, Q96L96 | 1.0 | 1 |
| Guanine-nucleotide dissociation stimulator, CDC24, conserved site | Q13009 | 1.0 | 10 |
| DENN domain | Q6ZUT9 | 1.0 | 15 |
| Chromo domain/shadow | P45973 | 1.0 | 7 |
| Chromo domain-like | P45973 | 1.0 | 7 |
| Tetratricopeptide repeat | P49792 | 1.0 | 2 |
| Lysosome-associated membrane glycoprotein, conserved site | P11279 | 1.0 | 78 |
| Transcription factor, fork head, conserved site | Q5VV16 | 1.0 | 7 |
| Cyclophilin-type peptidyl-prolyl cis-trans isomerase, conserved site | P49792 | 1.0 | 11 |
| Histone acetylase PCAF | Q92830 | 1.0 | 118 |
| Zinc finger, RING-type, conserved site | O76064 | 1.0 | 1 |
| Potassium channel, calcium-activated, BK, alpha subunit | A8MYU2 | 1.0 | 59 |
| BTB/POZ fold | Q8WZ60 | 1.0 | 1 |
| G protein-coupled receptor, rhodopsin-like | P51679 | 1.0 | 0 |
| Heat shock protein Hsp90 | P14625 | 1.0 | 16 |
| Post-SET domain | Q9UPS6 | 1.0 | 13 |
| RmlC-like jelly roll fold | Q9NS40 | 1.0 | 5 |
| Peptidase M13, N-terminal domain | P23276 | 1.0 | 29 |
| Zinc finger, RING-type | O76064 | 1.0 | 1 |
| Peptidase C2, calpain, catalytic domain | Q9Y6W3 | 1.0 | 16 |
| dDENN domain | Q6ZUT9 | 1.0 | 15 |
| HEAT repeat associated with sister chromatid cohesion protein | Q6KC79 | 1.0 | 118 |
| Glycoside hydrolase, superfamily | Q9UEF7 | 1.0 | 4 |
| C-type lectin | P07306 | 1.0 | 3 |
| Fringe | Q9Y644 | 1.0 | 78 |
| Glycoside hydrolase, family 1 | Q9UEF7 | 1.0 | 47 |
| Regulator of G protein signalling superfamily | Q9H3E2 | 1.0 | 7 |
| WD40/YVTN repeat-like-containing domain | Q8WUM0 | 1.0 | 1 |
| EF-hand domain | Q06190 | 1.0 | 1 |
| Domain of unknown function DUF4009 | Q9NUB1 | 1.0 | 11 |
| Nck-associated protein 5-like | O14513 | 1.0 | 118 |
| Fragile site-associated protein, C-terminal | Q2LD37 | 1.0 | 235 |
| Zinc finger, RanBP2-type | P49792 | 1.0 | 9 |
| Alpha/beta hydrolase fold-1 | P07098 | 1.0 | 8 |
| 3'5'-cyclic nucleotide phosphodiesterase, catalytic domain | Q14123 | 1.0 | 11 |
| Enhancer of polycomb-like, N-terminal | Q92613 | 1.0 | 29 |
| Ribonuclease H-like domain | O95789 | 1.0 | 3 |
| Bromodomain, conserved site | Q92830 | 1.0 | 9 |
| COMPASS complex Set1 subunit, N-SET domain | Q9UPS6 | 1.0 | 118 |
| Kelch-like protein, gigaxonin | Q8WZ60 | 1.0 | 5 |
| SET domain | Q9UPS6 | 1.0 | 4 |
| G protein alpha subunit, helical insertion | P84996 | 1.0 | 15 |
| Peptidase C2, calpain, large subunit, domain III | Q9Y6W3 | 1.0 | 18 |
| C2 calcium-dependent membrane targeting | Q8N5R6 | 1.0 | 2 |
| Acyl-CoA oxidase/dehydrogenase, central domain | O15254 | 1.0 | 16 |
| Kelch repeat type 2 | Q7Z6M1 | 1.0 | 47 |
| Phox-associated domain | Q9H3E2 | 1.0 | 59 |
| Stonin-2, N-terminal | Q8WXE9 | 1.0 | 235 |
| CDC48, N-terminal subdomain | Q8NB90 | 1.0 | 78 |
| Na/K/Cl co-transporter superfamily | Q9H2X9 | 1.0 | 34 |
| Cyclophilin-like peptidyl-prolyl cis-trans isomerase domain | P49792 | 1.0 | 10 |
| Ras-like guanine nucleotide exchange factor, N-terminal | Q8N9B8 | 1.0 | 10 |
| Ran binding domain | P49792 | 1.0 | 20 |
| Leucine-rich repeat | P08571 | 1.0 | 1 |
| TRASH domain | O95789 | 1.0 | 29 |
| Anoctamin/TMEM 16 | Q4KMQ2 | 1.0 | 21 |
| Potassium channel, voltage-dependent, ERG | Q9NS40 | 1.0 | 78 |
| Zinc finger, FYVE/PHD-type | Q92613 | 1.0 | 2 |
| ABC transporter, conserved site | P33527 | 1.0 | 5 |
| Ubiquitin-associated/translation elongation factor EF1B, N-terminal, eukaryote | Q9H7E2 | 1.0 | 5 |
| Arfaptin homology (AH) domain/BAR domain | Q8TDY4 | 1.0 | 3 |
| EF-Hand 1, calcium-binding site | Q06190 | 1.0 | 1 |
| Heat shock protein Hsp90, conserved site | P14625 | 1.0 | 47 |
| Nonaspanin (TM9SF) | Q9HD45 | 1.0 | 39 |
| Raf-like Ras-binding | Q13009 | 1.0 | 29 |
| Centrosomal protein of 128kDa | Q6ZU80 | 1.0 | 235 |
| PCAF, N-terminal | Q92830 | 1.0 | 78 |
| Zinc finger, PHD-finger | Q92613 | 1.0 | 3 |
| BTB/Kelch-associated | Q8WZ60 | 1.0 | 4 |
| G-protein alpha subunit, group S | P84996 | 1.0 | 118 |
| GPCR, rhodopsin-like, 7TM | P51679 | 1.0 | 0 |
| Folate receptor-like | P15328 | 1.0 | 29 |
| FERM domain | Q96L92 | 1.0 | 4 |
| RUN | Q6ZUT9 | 1.0 | 12 |
| Aspartate decarboxylase-like domain | Q8NB90 | 1.0 | 59 |
| Ribosomal protein S5 domain 2-type fold | P14625 | 1.0 | 5 |
| MIT | Q9Y6W3 | 1.0 | 21 |
| Homeobox, conserved site | P14653 | 1.0 | 1 |
| POU-specific | Q8N7G0 | 1.0 | 13 |
| Domain of unknown function DUF4371 | O95789 | 1.0 | 39 |
| Amino acid transporter, transmembrane | Q495M3 | 1.0 | 14 |
| Fanconi anaemia group A protein | O15360 | 1.0 | 235 |
| Mitochondrial fission regulator 1 | Q6P444 | 1.0 | 78 |
| BTB/POZ-like | Q8WZ60 | 1.0 | 1 |
| ATPase, AAA-type, core | Q8NB90 | 1.0 | 4 |
| Chromo domain subgroup | P45973 | 1.0 | 24 |
| Immunoglobulin I-set | Q96L96 | 1.0 | 2 |
| Tetratricopeptide-like helical | P49792 | 1.0 | 1 |
| Domain of unknown function DUF1767 | Q9H7E2 | 1.0 | 118 |
| Potassium channel, voltage-dependent, EAG/ELK/ERG | Q9NS40 | 1.0 | 17 |
| Chemokine receptor family | P51679 | 1.0 | 15 |
| Arf GTPase activating protein | Q8TDY4 | 1.0 | 8 |
| E3 ubiquitin ligase, RNF8 | O76064 | 1.0 | 235 |
| Link | P16070 | 1.0 | 16 |
| Nucleoporin, Nup133/Nup155-like, C-terminal | Q8WUM0 | 1.0 | 118 |
| Sushi/SCR/CCP | P08174 | 1.0 | 4 |
| Ribonucleotide reductase-related | Q7LG56 | 1.0 | 118 |
| AMP-binding, conserved site | Q9NUB1 | 1.0 | 9 |
| Lambda repressor-like, DNA-binding domain | Q8N7G0 | 1.0 | 8 |
| C-type lectin, conserved site | P07306 | 1.0 | 5 |
| Acyl-CoA oxidase | O15254 | 1.0 | 59 |
| Tudor domain | Q9H7E2 | 1.0 | 7 |
| Protein of unknown function DUF2371, TMEM200 | Q86VY9 | 1.0 | 78 |
| Chromo domain | P45973 | 1.0 | 9 |
| UBA-like | Q9H7E2 | 1.0 | 4 |
| Ras-association | Q96L92 | 1.0 | 6 |
| Stonin homology | Q8WXE9 | 1.0 | 78 |
| Metallopeptidase, catalytic domain | P23276 | 1.0 | 3 |
| EEIG1/EHBP1 N-terminal domain | Q8NDI1 | 1.0 | 59 |
| Guanine-nucleotide dissociation stimulator CDC25 | Q8N9B8 | 1.0 | 7 |
| Zinc finger, LIM-type | Q5TD97 | 1.0 | 3 |
| Dynein heavy chain | Q8TD57 | 1.0 | 24 |
| Histidine kinase-like ATPase, ATP-binding domain | P14625 | 1.0 | 14 |
| Galactose-binding domain-like | Q08345 | 1.0 | 3 |
| Lipoxygenase, LH2 | Q6ZUT9 | 1.0 | 11 |
| 3'5'-cyclic nucleotide phosphodiesterase N-terminal | Q14123 | 1.0 | 78 |
| GNAT domain | Q92830 | 1.0 | 9 |
| POU domain | Q8N7G0 | 1.0 | 13 |
| uDENN domain | Q6ZUT9 | 1.0 | 15 |
| Fibronectin, type III | P08922 | 1.0 | 1 |
| Cyclic nucleotide-binding domain | Q9NS40 | 1.0 | 7 |
| Sister chromatid cohesion C-terminal domain | Q6KC79 | 1.0 | 235 |
| CC chemokine receptor 4 | P51679 | 1.0 | 235 |
| Ras guanine nucleotide exchange factor | Q8N9B8 | 1.0 | 9 |
| ABC transporter-like | P33527 | 1.0 | 5 |
| Multi drug resistance-associated protein | P33527 | 1.0 | 59 |
| Neuroendocrine-specific golgi P55 | P84996 | 1.0 | 235 |
| Homeodomain, metazoa | P14653 | 1.0 | 3 |
| Coagulation factor 5/8 C-terminal type domain | Q08345 | 1.0 | 8 |
| Lysosome-associated membrane glycoprotein | P11279 | 1.0 | 47 |
| Cyclic nucleotide-binding-like | Q9NS40 | 1.0 | 6 |
| K/Cl co-transporter, type 1/type 3 | Q9H2X9 | 1.0 | 29 |
| Peptidase M13 | P23276 | 1.0 | 29 |
| Protein of unknown function DUF2454 | Q6NXR4 | 1.0 | 235 |
| 3'5'-cyclic nucleotide phosphodiesterase | Q14123 | 1.0 | 12 |
| Stonin | Q8WXE9 | 1.0 | 118 |
| LDLR class B repeat | P08922 | 1.0 | 16 |
| Acyl-CoA N-acyltransferase | Q92830 | 1.0 | 5 |
| Small GTPase superfamily, Ras type | Q9Y3L5 | 1.0 | 9 |
| Fringe-like | Q9Y644 | 1.0 | 47 |
| Peptidase C13, legumain | Q99538 | 1.0 | 118 |
| HD/PDEase domain | Q14123 | 1.0 | 9 |
| Amino acid permease domain | Q9H2X9 | 1.0 | 26 |
| Muniscin C-terminal mu homology domain | Q9BQI5 | 1.0 | 78 |
| Serine/threonine-protein kinase, active site | Q9NRP7 | 1.0 | 1 |
| Ribonucleotide reductase small subunit | Q7LG56 | 1.0 | 118 |
| Zinc finger, MYM-type | O95789 | 1.0 | 39 |
| Chromo shadow domain | P45973 | 1.0 | 78 |
| Peptidase M13, C-terminal domain | P23276 | 1.0 | 29 |
| Ferritin-like superfamily | Q7LG56 | 1.0 | 26 |
| Transcription factor, fork head | Q5VV16 | 1.0 | 5 |
| ATPase, AAA-type, conserved site | Q8NB90 | 1.0 | 8 |
| Nucleotide-binding, alpha-beta plait | Q9UPS6 | 1.0 | 1 |
| Chromo domain, conserved site | P45973 | 1.0 | 12 |
| Bromodomain | Q92830 | 1.0 | 5 |
| Nup358/RanBP2 E3 ligase domain | P49792 | 1.0 | 235 |
| Sorting nexin, C-terminal | Q9H3E2 | 1.0 | 59 |
| Peptidase C2, calpain, domain III | Q9Y6W3 | 1.0 | 20 |
| MHCK/EF2 kinase | Q96L96 | 1.0 | 39 |
| Peptidase C2, calpain family | Q9Y6W3 | 1.0 | 17 |
| EF-hand-like domain | Q06190 | 1.0 | 1 |
| Protein of unknown function DUF1370, TMEM126 | Q9H061 | 1.0 | 118 |
| ABC transporter, transmembrane domain, type 1 | P33527 | 1.0 | 8 |
| 3'5'-cyclic nucleotide phosphodiesterase, conserved site | Q14123 | 1.0 | 11 |
| Dbl homology (DH) domain | Q13009 | 1.0 | 3 |
| Intermediate filament protein, conserved site | P48681 | 1.0 | 4 |
| POU domain-containing protein, class 5 | Q8N7G0 | 1.0 | 47 |
| Heat shock protein Hsp90, N-terminal | P14625 | 1.0 | 24 |
| Ras guanine nucleotide exchange factor, domain | Q8N9B8 | 1.0 | 7 |
| Six-bladed beta-propeller, TolB-like | P08922 | 1.0 | 6 |
| Zinc finger, PHD-type, conserved site | Q92613 | 1.0 | 3 |
| CD44 antigen | P16070 | 1.0 | 235 |
| PAS domain | Q9NS40 | 1.0 | 6 |
| AMP-dependent synthetase/ligase | Q9NUB1 | 1.0 | 8 |
| Domain of unknown function DUF3585 | Q8NDI1 | 1.0 | 34 |
| RNA recognition motif domain | Q9UPS6 | 1.0 | 1 |
| Acetate-CoA ligase | Q9NUB1 | 1.0 | 118 |
| Folate receptor | P15328 | 1.0 | 47 |
| Acyl-CoA oxidase, C-terminal | O15254 | 1.0 | 59 |
| Acyl-CoA dehydrogenase/oxidase | O15254 | 1.0 | 16 |
| Guanine nucleotide binding protein (G-protein), alpha subunit | P84996 | 1.0 | 15 |
| Monocyte differentiation antigen CD14 | P08571 | 1.0 | 235 |
| K-Cl co-transporter | Q9H2X9 | 1.0 | 59 |
| Lipase, eukaryotic | P07098 | 1.0 | 39 |
